# Exploring Alkyl-O-Alkyl Ether Structures in Softwood Milled Wood Lignins

**DOI:** 10.1021/acs.jafc.2c06375

**Published:** 2022-12-21

**Authors:** Xuhai Zhu, Jussi Sipilä, Antje Potthast, Thomas Rosenau, Mikhail Balakshin

**Affiliations:** † Department of Bioproducts and Biosystems, School of Chemical Engineering, Aalto University, Vuorimiehentie 1, Espoo 02150, Finland; ‡ Laboratory of Organic Material Chemistry, Department of Chemistry, University of Helsinki, P.O. 55 (A. I. Virtasen Aukio 1), Helsinki 00014, Finland; § Department of Chemistry, Institute for Chemistry of Renewable Resources, University of Natural Resources and Life Sciences (BOKU), Muthgasse 18, Vienna 1190, Austria

**Keywords:** alkyl ether structure, biorefinery, lignin, lignin structure, milled wood lignin, NMR

## Abstract

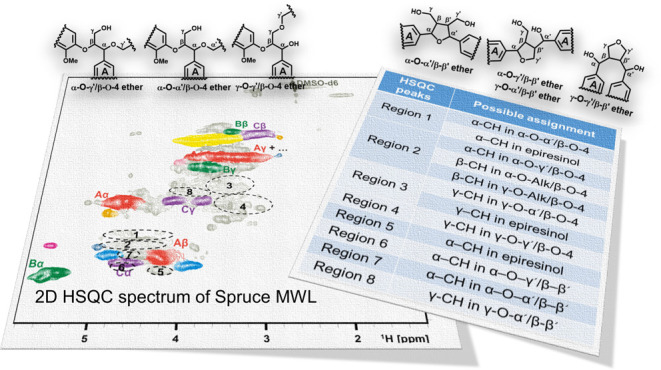

Recent studies have suggested that there are significant amounts of various alkyl ether (Alk-O-Alk; Alk = alkyl) moieties in a spruce native lignin preparation, milled wood lignin (SMWL). However, the comprehensive NMR assignment to these moieties has not been addressed yet. This study focused on investigating different types of Alk-O-Alk structures at the α- and γ-positions of the lignin side chain in an heteronuclear single-quantum coherence (HSQC) spectrum of SMWL using experimental NMR data of lignin and synthesized model compounds. Ambiguous structural features were predicted by computer simulation of ^1^H and ^13^C NMR spectra to complement the experimental NMR data. As a result, specific regions in the HSQC spectrum were attributed to different Alk-O-Alk moieties of Alk-O-Alk/β-O-4 and Alk-O-Alk/β-β′ structures. However, the differences between the specific regions were rather subtle; they were not well separated from each other and some major lignin moieties. Furthermore, SMWL contained a large variety of Alk-O-Alk moieties but in minute individual amounts, resulting in rather broad, superimposing resonances. Thus, evaluation did not allow assigning individual types of Alk-O-Alk moieties from the HSQC spectra; instead, they were quantified as total (α- and γ-linked) Alk-O-Alk based on the balance of structural units in the ^13^C NMR spectra. At last, potential formation mechanisms of various Alk-O-Alk ether structures in lignin biosynthesis, lignin aging, and during ball milling of wood were hypothesized and discussed.

## Introduction

Lignin is a very complex heterogeneous aromatic polymer. Despite the tremendous number of studies, some elements of its chemical structure are still under discussion. For example, the current analytical methodology allows only the description of 80–85% of structural moieties in native lignin preparations, such as milled wood lignins (MWLs).^1−3^ The structures of the remaining 15–20% of lignin units are not well understood. Based on the overall material balance and specific features in the ^13^C NMR spectra along with literature data for comparison, they were tentatively assigned to various types of alkyl ether (Alk-O-Alk) moieties at the α- and γ-positions of the side chain.^1−3^

Alk-O-Alk moieties, that is, aliphatic ether structures, may play an important role in lignin branching—in agreement with our view that the macromolecular structure of lignin is a three-dimensional network rather than a linear chain^1,2^—in addition to already identified structural units, such as etherified biphenyl (5-5′) and diaryl ether (4-O-5′) structures, which are among the main branching points in lignin. It was suggested that about 20–28% of monolignols in softwood lignins were involved in 5-5′ (major) and 4-O-5′ (minor) linkages based on CuO-permanganate oxidation (PO), ^13^C NMR, and thioacidolysis-^31^P NMR methods.^1,2,4,5^ The analysis of the absolute molecular mass and the number of terminal units in spruce MWL (SMWL) showed that it is even more branched/crosslinked than expected solely from the quantification of the known branching points at the aromatic rings.^2^ Only half of the branching points were located there, whereas the other half was expected to be located in the side chains. They were assigned to various Alk-O-Alk structures at the α- or γ-positions of the side chain, with additional β-O-4 or β-β′ linkages (Alk-O-Alk/β-O-4, β-β ether structure).^2^

Although the occurrence of Alk-O-Alk structures in native lignin is still not commonly accepted, their presence has been proposed and discussed earlier.^6−10^ For the noncyclic Alk-O-Alk/β-O-4 ether structures, Leary^6,7^ proposed that 17–21 benzyl noncyclic alkyl ether groups per 100 C9 units might occur in spruce Björkman lignin, based on Adler’s studies.^8^ Glasser *et al.*(9) suggested 25% of noncyclic α-O-γ′ ether linkages based on computer simulation of softwood lignin structure. Another piece of evidence was provided by Sakakibara’s group, who isolated an α-O-γ′ ether dimer from lignin hydrogenolysis products.^10^ Regarding the cyclic Alk-O-Alk/β-β′ ether structure, cyclic α-O-α′ and α-O-γ′ ether linkages were reported by Ralph and Lu,^11^ identified in syringyl lignin from palm, kenaf, and corn cell walls. Zhang and Gellerstedt^12^ even found a guaiacyl analogue of cyclic α-O-α′ether with β-β′ linkages in an SMWL in relatively small amounts but no a γ-O-Alk ether analogue. Bicyclic epiresinol structures have previously been suggested for hardwood kraft lignin by Bruijnincx *et al.*(13)

**Figure 1:**
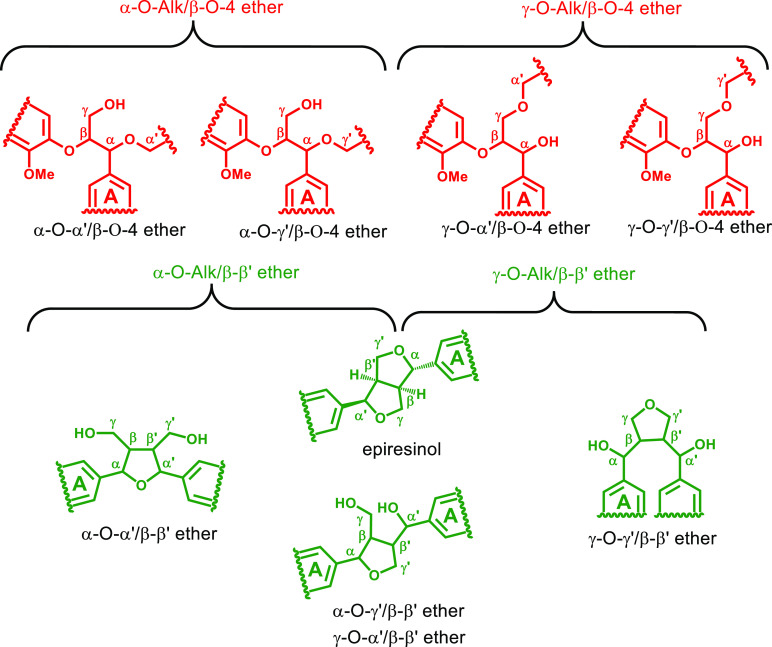
General types of Alk-O-Alk moieties.

Alk-O-Alk moieties are often unstable toward wet chemistry methods used in lignin analysis, which easily transform into aliphatic alcohols. Therefore, they are difficult to detect upon degradative analysis of lignin. Nondegradative techniques, in particular NMR spectroscopic techniques, such as heteronuclear single-quantum coherence (HSQC), are especially valuable for their direct detection. Recent scrutiny of softwood MWL (including 2D NMR) showed some unassigned signals,^2,3^ which so far had been disregarded. We suggested they belong to a variety of Alk-O-Alk ether structures in general.^1−3^ However, their structural information is still ambiguous, and the exact formation during lignin biosynthesis is still unclear. Therefore, the present study is devoted to the elaboration of Alk-O-Alk ether moieties in SMWL (Figure 1) using experimental data of lignins and relevant model compounds complemented by computer modeling. A selection of most important structures under discussion will have to be further corroborated by synthesis of specific model compounds and their NMR analysis in future studies.

## Experimental Section

### General

All chemicals and solvents were purchased from Sigma-Aldrich and used without further purification, except dioxane which was distilled over sodium hydroxide before each use. The information on the selected model compounds is described in the Supporting Information. Computationally unsophisticated simulation of ^1^H and ^13^C NMR spectra was performed with the ChemDraw Professional v19.0 package.^14−18^

### Isolation of MWL

Spruce (*Picea abies*) wood meal (60 mesh pass) was preextracted with ethanol/toluene, 1:2 (v/v), in a Soxhlet apparatus to remove lipophilic extractives. The MWL preparation was isolated from the preextracted wood meal and purified as described earlier.^3,19^ The yield of the purified SMWL was about 25% per Klason lignin content in wood.

### HSQC NMR Experiment

The MWL (80 mg) was fully dissolved in 0.6 ml of DMSO-*d*_6_. A high-resolution HSQC spectrum was acquired with a Bruker AVNEO 600 MHz spectrometer equipped with a 5 mm He-cooled TCI gradient cryoprobe using a Bruker pulse program “hsqcetgpsisp.2” with maximum sensitivity enhancement. 1024 data points were acquired at 298 K, from 11 to 0 ppm in F2 (^1^H), with an acquisition time of 77.8 ms, and from 215 to 0 ppm in F1 (^13^C) with 256 increments, 36 scans, and a 2.0 s interscan delay. The heteronuclear coupling constant value was set at 145 Hz. Processing the final matrix to 2 K by 1 K data points was performed by QSINE window functions in both F2 and F1. The spectral processing was carried out with Bruker’s Topspin 4.0 (Windows) software. The central peak of the residual solvent (δ_H_ 2.49, δ_C_ 39.5 ppm) was used for calibration. All known correlation peaks were assigned based on earlier reports.^2,3,20−26^

## Results and Discussion

### HSQC NMR Spectrum of SMWL

**Figure 2:**
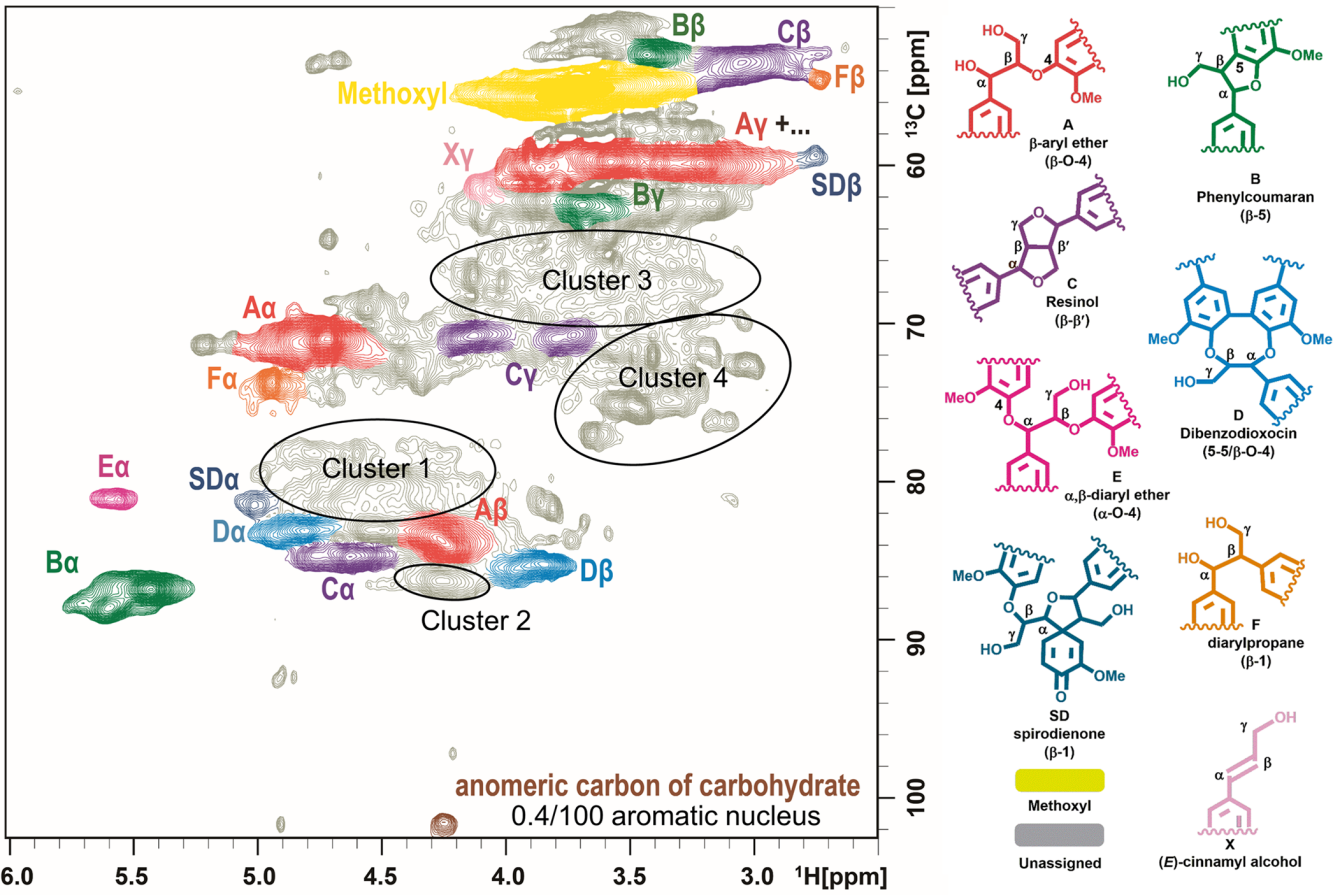
Partial short-range ^1^H–^13^C HSQC NMR spectrum (oxygenated aliphatic region) of SMWL, in DMSO-*d*_6_. The carbohydrate content was calculated by integration shown in Figure S1.

The HSQC spectrum of SMWL (Figure 2) shows common and expectable structural characteristics of this analysis. Different correlation signals in the oxygenated aliphatic region were assigned to β-O-4 (aryl ether), β-5 (phenylcoumaran), β-β′(resinol), DBDO (dibenzodioxocins), β-1 (diarylpropane), SD (spirodienone), and cinnamyl alcohol structures. Only very small cross-peaks of H_1_–C_1_ of carbohydrates (*ca.* 0.4%) were found in the well-resolved anomeric region (δ_H_/δ_C_: 3.9–5.5/90–105 ppm). This agreed with the result of the wet chemistry analysis, which reported the carbohydrate content of about 0.7%. Therefore, the influence of carbohydrates on the analysis of lignin structure was very minor for this sample.

As shown in Figure 2, unassigned signals are mainly found in four regions of the spectrum: cluster 1 (δ_H_/δ_C_: 4.1–4.9/77–83 ppm), cluster 2 (δ_H_/δ_C_: 4.1–4.4/85–87 ppm), cluster 3 (δ_H_/δ_C_: 3.1–4.3/64–70 ppm), and cluster 4 (δ_H_/δ_C_: 3.0–3.7/71–78 ppm). Previous study about lignin–carbohydrate complexes (LCCs) showed that the H_α_–C_α_ and H_β_–C_β_ correlations of benzyl ether LCC linkages were located in cluster 1.^23,27,28^ However, this contribution cannot be significant in the present case due to the negligible amount of carbohydrate contained in the SMWL. Alternatively, it was suggested that the correlations in clusters 1 and 2 may originate from the α-position (CH-α) of benzyl alkyl ether moieties and the β-position (CH-β) of β-aryl ether moieties in an α-O-Alk/β-O-4 ether structure^2,3^ and that clusters 3 and 4 with overlapping resonances contain a variety of γ-ether moieties.^2,3^ Further compelling structural evidence of Alk-O-Alk ether structures in lignin has not yet been presented.

**Figure 3:**
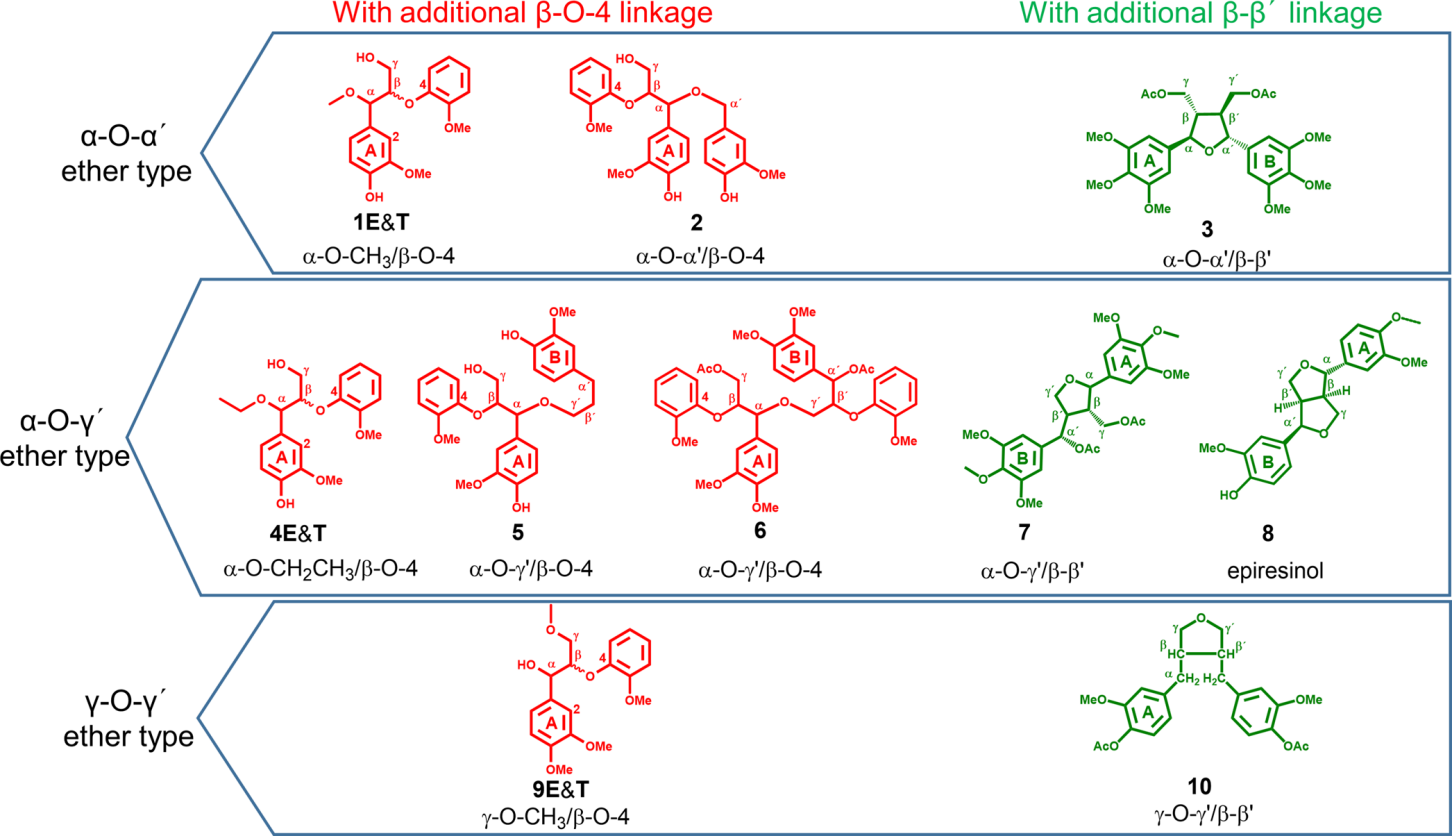
Structures of the synthesized lignin Alk-O-Alk ether model compounds **1–10**.

Two approaches were followed to explore the presence of proposed Alk-O-Alk ether structures in SMWL: model compounds with Alk-O-Alk ether moieties were synthesized (Figure 3), and some of their NMR data were reviewed in light of previous studies (Table 1).^11,28−33^ To address additional structural features, this was complemented by simulated Alk-O-Alk models and their spectra (Figure 5), which were plotted against the experimental HSQC spectrum of SMWL to assist with possible assignments.

**Table 1:** NMR Data for the Side Chain of the Synthesized Alk-O-Alk Ether Model Compounds **1–10** (*cf*. Figure 3)

model information				NMR chemical shift on the side chain, δ_H_/δ_C_, ppm			
ether type	no.	structural description	references	unit	α or α′	β or β′	γ or γ′
α-O-α′	**1E**	α-OCH_3_/β-O-4/γ-OH (*erythro* form)	in DMSO-*d*_6_^a^	A	4.34/81.9	4.42/82.3	3.56, 3.52/59.8
	**1T**	α-OCH_3_/β-O-4/γ-OH (*threo* form)	in DMSO-*d*_6_^a^	A	4.35/82.1	4.31/83.0	3.53, 3.30/60.0
	**2**	α-O-benzyl/β-O-4/γ-OH	28^b^, in CDCl_3_	A	4.70/80.1	4.54/81.4	4.07, 4.27/63.4
	**3**	α-O-α′/β-β′/γ-OAc	11^b^, in CDCl_3_	A,B	5.00/84.1	2.52/51.4	4.26/64.2
α–O−γ′	**4E**	α-OCH_2_CH_3_/β-O-4/γ-OH (*erythro* form)	in DMSO-*d*_6_^a^	A	4.45/79.8	4.38/82.5	3.60, 3.56/59.8
		–OCH_2_CH_3_	in DMSO-*d*_6_^a^	B			3.32/63.6
	**4T**	α-OCH_2_CH_3_/β-O-4/γ-OH (*threo* form)	in DMSO-*d*_6_^a^	A	4.45/80.1	4.28/83.5	3.32, 3.55/60.2
		–OCH_2_CH_3_	in DMSO-*d*_6_^a^	B			3.30/63.9
	**5**	α-O-γ′/β-O-4/γ-OH (mixture)	29^b^, in CDCl_3_	A	4.6/80.9,81.8	4.4–4.6/82.5	4.07, 4.29/63.7, 63.9
		α′-CH_2_/β′-CH_2_/γ′-O-α (mixture)	29^b^, in CDCl_3_	B	2.6–2.8/32.4	1.8–2.1/31.5	3.37–3.58/69.1
	**6**	α-O-γ′/β-O-4/γ-OAc (mixture)	in CDCl_3_^a^	A	4.70/80.9	4.4–4.6/81.8	4.38/64.0
		α′-OAc/β′-O-4/γ′-O-α (mixture)	in CDCl_3_^a^	B	6.05/75.0	4.4–4.6/82.0	3.55/68.2
	**7**	α-O-γ′/β-β′/γ-OAc	11^b^, in CDCl_3_	A	4.81/84.8	2.39/49.5	4.20, 4.45/63.6
		α′-OAc/β′-β/γ′-O-α (α′*S*)	11^b^, in CDCl_3_	B	4.86/72.5	2.92/48.0	4.07, 4.15/69.0
	**8**	α-O-γ′/β–β′/γ-O-α′	13^b^, in DMSO-*d*_6_	A	4.34/87.0	2.84/53.7	3.75, 4.06/70.2
		α′-O-γ/β′-β/γ′-O-α	13^b^, in DMSO-*d*_6_	B	4.77/81.2	3.70/49.2	3.12, 3.77/68.8
γ–O−γ′	**9E**	α-OH/β-O-4/γ-OCH_3_ (*erythro* form)	30^b^, in CDCl_3_	A	4.89/73.1	4.36/85.3	3.45, 3.65/71.6
	**9T**	α-OH/β-O-4/γ-OCH_3_ (*threo* form)	30^b^, in CDCl_3_	A	4.90/74.2	4.11/88.0	3.37, 3.50/71.8
	**10**	α-CH_2_/β–β′/γ-O-γ′	31,32^b^, in acetone-*d*_6_/CDCl_3_	A, B	2.59, 2.69/39.8	2.23/47.1	3.55, 3.93/73.5

a Detailed information available in Figures S3–S7.

b Number in the reference list.

### Structural Identification Based on Experimental NMR Data

**Figure 4:**
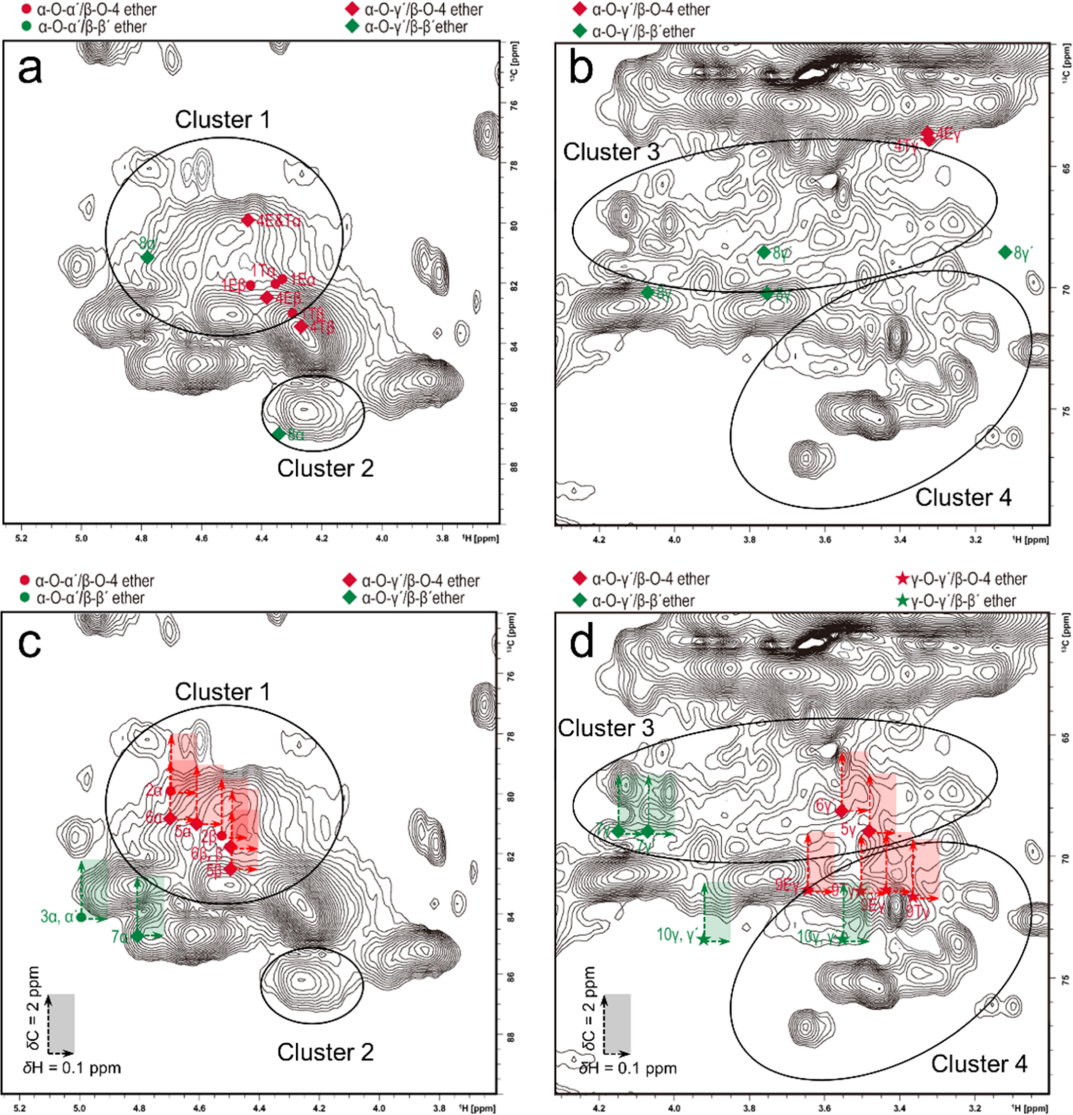
HSQC spectrum of SMWL overlaid with the experimental NMR data of Alk-O-Alk ether models **1–10**: signals of α-O-Alk ether model compounds collected in DMSO-*d*_6_ (a) and CDCl_3_ (c) are located at the region δ_H_/δ_C_, 3.8–5.0/78–88 ppm; signals of γ-O-Alk ether model compounds collected in DMSO-*d*_6_ (b) and CDCl_3_ (d) are located in the region δ_H_/δ_C_, 3.0–4.2/64–78 ppm. The solvent shift effect (Δδ_H_ = ∼0.1 ppm, Δδ_C_ = ∼2 ppm) is indicated by arrows that span the colored rectangular shift regions.

As shown in Figure 3 and Table 1, the synthesized model compounds can be classified into three types, that is, α-O-α′ (compounds **1–3**), α-O-γ′ (compounds **4–8**), and γ-O-γ′ ethers (compounds **9** and **10**). The model compounds in red (**1**, **2**, **4**, **5**, **6**, and **9**) represent noncyclic alkyl ether with an additional β-O-4 linkage (Alk-O-Alk/β-O-4), and model compounds in green (**3**, **7**, **8**, and **10**) have cyclic structures formed by α-O-Alk or γ-O-Alk together with β-β′ linkages (Alk-O-Alk/β-β′). The NMR data of models **1**, **4**, and **8** were recorded in DMSO-*d*_6_, which were the same as those recorded in the solvent for the HSQC spectrum of SMWL. The direct spectral comparison is shown in Figure 4a,b. The other model compounds were analyzed in CDCl_3_, and the chemical shift difference between SMWL and model compounds caused by the solvent effect had to be considered in this study. According to the NMR database of lignin compounds,^20^ the variation between DMSO-*d*_6_ and CDCl_3_ was generally 0.1 ppm for ^1^H and 2 ppm for ^13^C (Δδ_H_ = ∼0.1 ppm, Δδ_C_ = ∼2 ppm) or less. As a rule of thumb, CDCl_3_ gave a higher δ_C_ (downfield shift by up to 2 ppm) and a higher δ_H_ (downfield shift by up to 0.1 ppm) than DMSO-*d*_6_, which need to be considered when comparing the NMR data with SMWL measured in DMSO-*d*_6_. As a consequence, the central points of the cross-peaks in spectra measured in CDCl_3_ should shift toward the upper right quadrant in the circled regions when making the transition to DMSO-*d*_6_, as indicated by the colored squares in Figure 4c,d. In addition, NMR data for LCC model compounds in Table S3 and S. Ralph’s database^20^ indicated that acetylation at the γ-position affected the chemical shifts of γ- and β-CH but not that at the α-position. Therefore, the NMR data of the α-alkyl positions in the γ-acetylated model compounds **3**, **6**, and **7** can be directly used and compared with those of nonacetylated lignin.

When the chemical shift values of α-O-Alk (α-O-α′ and α-O-γ′)/β-O-4 ether-type model compounds collected in DMSO-*d*_6_ (from Table 1) were superimposed with those of the SMWL spectrum (Figure 4a), it was evident that the H_α_–C_α_ and H_β_–C_β_ correlations from models **1** and **4** matched well, suggesting that the H–C correlation cluster 1 (δ_H_/δ_C_, 4.1–4.9/77–83 ppm) in the SMWL spectrum comprises both H_α_–C_α_ and H_β_–C_β_ correlations of α-O-Alk/β-O-4 ether type. For the α-O-Alk/β-O-4 ether models, the H_α_–C_α_ correlations were always in the “upper” region of cluster 1 relative to the H_β_–C_β_ correlations. It was clear that the H_α_–C_α_ or H_β_–C_β_ correlations for *erythro* and *threo* forms of α-O-Alk/β-O-4 were close to each other despite the different benzyl environments. It was also observed in Figure 4b that the H_γ_–C_γ_ correlations of α-O-γ′/β-O-4 ether-type model **4** partly overlapped with the boundary of cluster 3. Apparently, this model compound did not sufficiently closely reflect the real structural environment around the alkyl-etherified γ-position in lignins. This will be addressed in future work by refined model compound selection.

The NMR data of epiresinol structure (compound **8**, α-O-γ′/β-β′ ether type) recorded in DMSO-*d*_6_ overlap well with those of clusters 1, 2, and 3 (Figure 4a,b): one of its H_α_–C_α_ cross peaks at δ_H_/δ_C_ 4.77/81.2 ppm was located at cluster 1, and the other H_α_–C_α_ cross peak at δ_H_/δ_C_ 4.34/87.0 ppm was at cluster 2. Figure 4b shows that one of the H_γ_–C_γ_ correlations at δ_H_/δ_C_ 3.75,4.06/70.2 ppm overlaps with the H_γ_–C_γ_ correlations of the resinol β-β′ structure and that the other H_γ_-C_γ_ correlations at δ_H_/δ_C_ 3.12,3.77/68.8 ppm lie in cluster 3. The epiresinol structure has only been previously reported for hardwood kraft lignin;^13^ its possible formation mechanism in softwood lignin will be discussed below. It is important to note that the correlations for compound **8** were different enough from those of model compounds **1** and **4** and that in lignin the α-O-Alk ether moieties with β-O-4 linkage can be distinguished from that with a β-β′ linkage by HSQC NMR.

Considering the effect of CDCl_3_ on the chemical shift (vs. DMSO-*d*_6_), Figure 4c suggests that the broad cluster 1 encompasses both H_α_–C_α_ and H_β_–C_β_ correlations of model compounds **2**, **5**, and **6** (α-O-Alk/β-O-4 ether). The right section of cluster 3, centered at around δ_H_/δ_C_, 3.5/68 ppm (Figure 4d), contains the chemical shifts of alkyl ethers in the γ-position of models **5** and **6** (α-O-γ′/β-O-4 ether). Earlier, Kilpelainen *et al.*(33) proposed that the HMQC cross-peak at δ_H_/δ_C_: 3.5–3.7/69 ppm in spectra of acetylated hardwood and softwood MWLs belonged to the H_γ_–C_γ_ correlation of compounds like **5**. Nevertheless, they did not exclude the possibility that other types of γ-O-Alk/β-O-4 ether structures also contribute. For the simple γ-O-Me/β-O-4 ether model compound **9**, the H_γ_–C_γ_ correlation lies at the boundary between clusters 3 and 4, centered at around δ_H_/δ_C_, 3.6/70 ppm. Based on Table S3, one can say that there was no difference in the chemical shift of the side-chain CH between the syringyl and guaiacyl β-β′ model compounds. The NMR data of syringyl model compounds **3** and **7** can thus be directly used in lieu of the guaiacyl analogue. As shown in Figure 4a, the H_α_–C_α_ correlations from α-O-Alk/β-β′ether compounds **3** and **7** were close to those of typical DBDO and resinol structures. As seen in Figure 4d, the H_γ_–C_γ_ correlations of model compound **7** (γ-O-α′/β–β′ether) appear on the left side of cluster 3 and those of compound **10** (γ-O-γ′/β-β′ether) in cluster 4. This analysis confirmed that α-O-Alk/β-β′ ether structures can be easily distinguished from the α-O-Alk/β-O-4 ether structures.

### Structural Identification with Simulated NMR Data

Computational models can help to assign Alk-O-Alk ether structures and to extensively explore structure–shift correlations, especially in cases of synthetically hard to access model compounds or when minor structural differences are to be studied. Of course, there is some limitation caused by computational error limits and an uncertainty with regard to consideration of solvent effects, yet the method is still very helpful and widely applied in structural identification. The model compounds VG, GG, and GH in Table S3 from previous studies^20,34^ was employed to evaluate the quality of the computational estimation of the NMR data. The difference between experimental and simulated NMR shifts, as shown in Table S3, was significant both for the ^1^H and ^13^C domains (Δδ_H_ = 0–0.3 ppm; Δδ_C_ = 0–8 ppm), implying that the simulated NMR data of the Alk-O-Alk ether structures cannot directly be utilized. Therefore, we used a relative comparison with the simulated NMR data based on the effect of different alkyl ether substituents on the chemical shifts of the side-chains of well-known β-O-4 (α-OH/β-O-4/γ-OH) structures and β-β′ (resinol) structures, as described in the following.

#### Alk-O-Alk/β-O-4 Ether Structures

**Figure 5:**
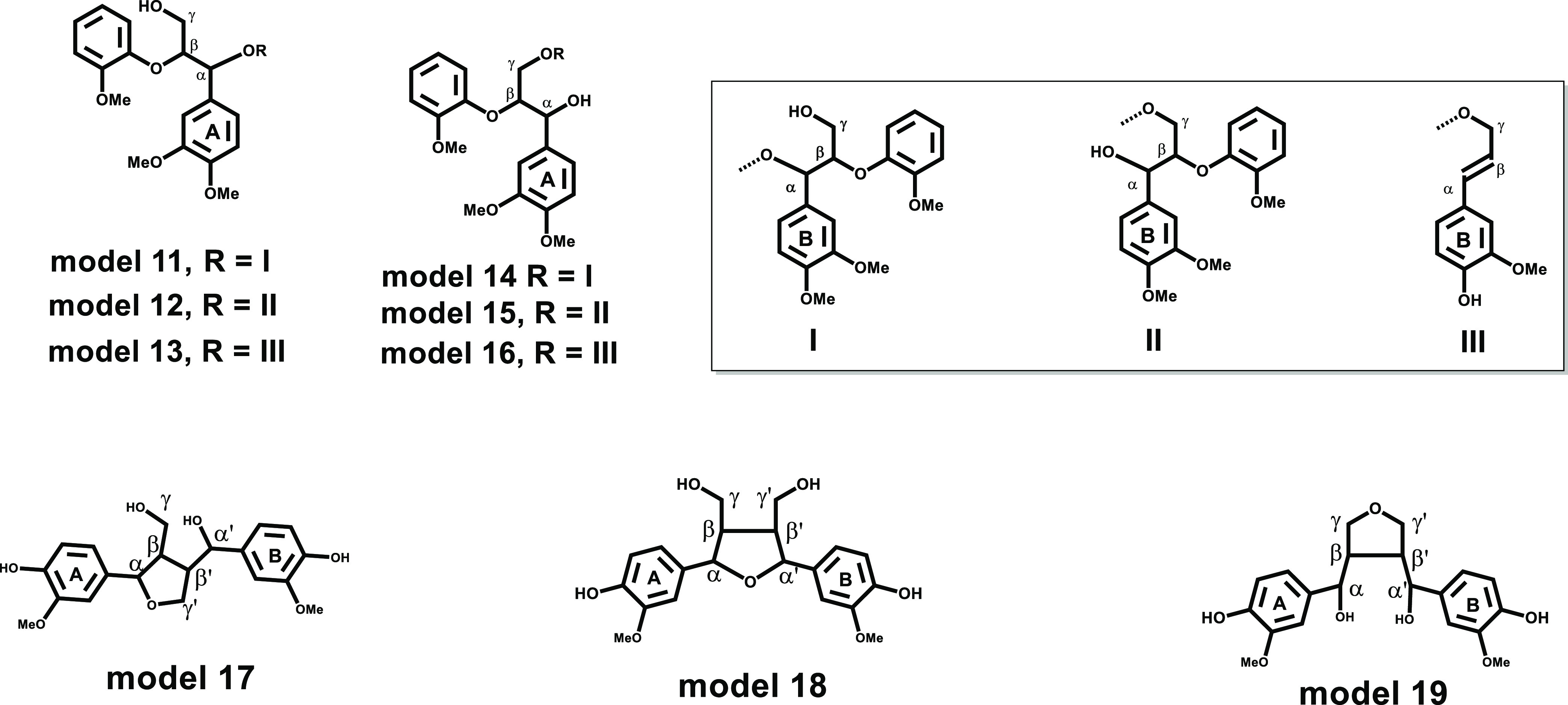
Alk-O-Alk ether models **11–19** used in computer simulation studies.

**Figure 6:**
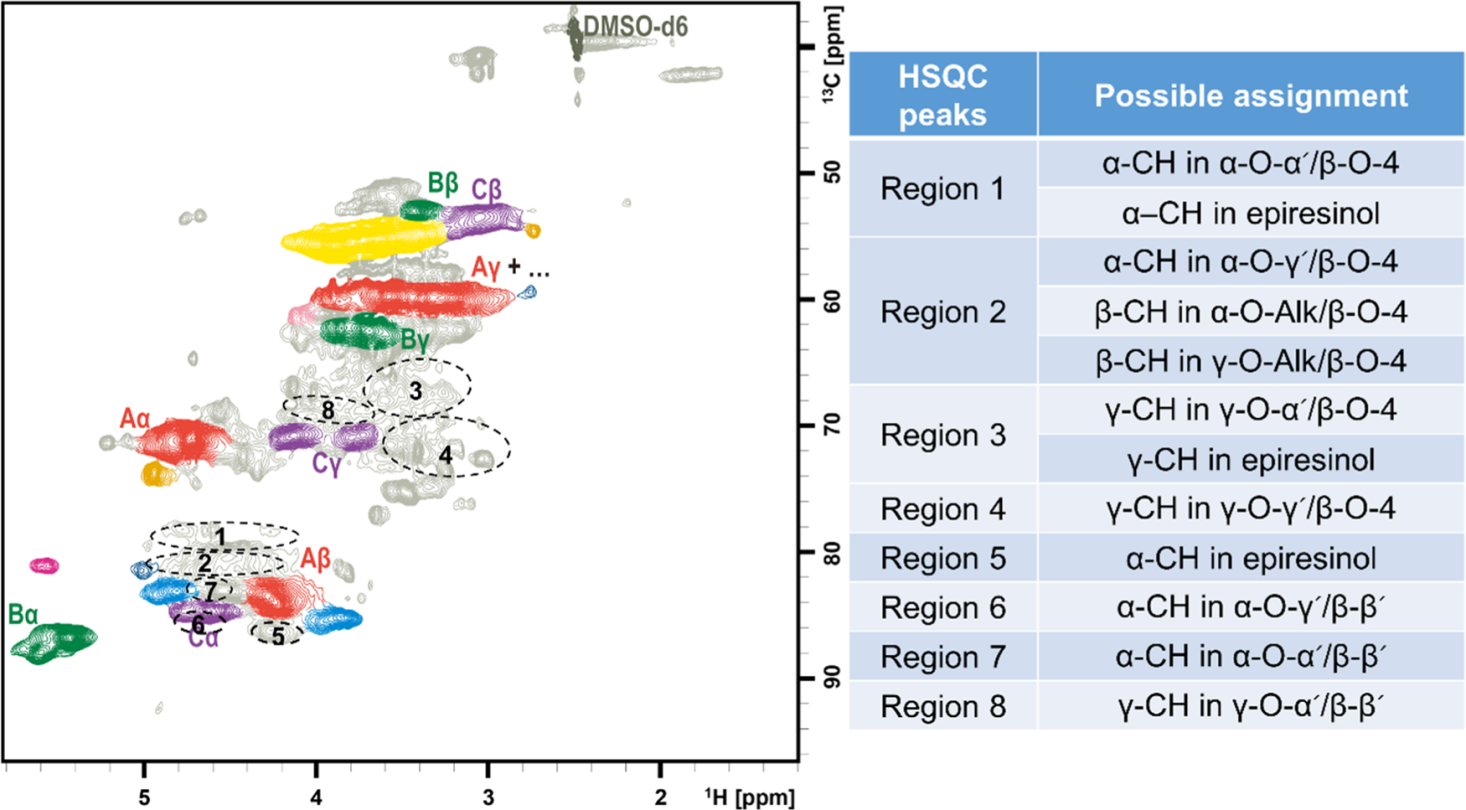
HSQC spectrum of SMWL with new structural assignments proposed using data simulation.

To investigate the effect of alkyl etherification on the side-chain chemical shifts of β-O-4 structures (Figure 5), models of a β-O-4 dimer (VG) etherified at α- or γ-OH positions with three different types of alkyl moieties (I, II, and III) were computationally studied: the α-O-Alk/β-O-4 models **11–13** and γ-O-Alk/β-O-4 models **14–16**. As shown in Table 2, etherification of α-OH in models **11–13** resulted in an upfield shift of δ_H_ (Δδ_H_ = −∼0.3 ppm) and a downfield shift of δ_C_ (Δδ_C_ = +∼11–14 ppm) for α-CH as compared to the nonetherified VG parent model (α-OH/β-O-4/γ-OH). A similar trend was observed in the case of γ-etherification (γ-O-Alk/β-O-4 models **14–16**): Δδ_H_ = ∼−0.2 ppm and Δδ_C_ = ∼+6–8 ppm for γ-CH. Furthermore, comparing the Δδ_C_ values of α-CH of model **11** versus **12** (or **14** vs **15**) showed that the effect of γ′-alkyl etherification of another β-O-4 unit (in structures II or III) was larger than that of α′-alkyl (in structure I), with Δδ_C_ = ∼8–14 ppm for γ′-Alk being larger than Δδ_C_ = ∼6–11 for α′-Alk. This was consistent with a tendency observed for the synthesized model compounds (VG vs models **1E**, **2**, **6**, and **9E** in Table S4). Thus, in Figure 6, the spectral region 1 and the spectral region 2, indicated by circles, were specifically attributed to α-CH of α-O-α′/β-O-4 and α-O-γ′/β-O-4 ether structures, respectively.

**Table 2:** Simulated NMR Data for Side Chains of Alk-O-Alk/β-O-4 Ether Models

			estimated NMR data of the side chain					
	structure description		α, ppm		β, ppm		γ, ppm	
models	A unit	B unit	δ_H_/Δ^e^	δ_C_/Δ	δ_H_/Δ	δ_C_/Δ	δ_H_/Δ	δ_C_/Δ
ref^d^, VG	α-OH/β-O-4/γ-OH		5.28	72.8	4.08	91.1	3.76, 3.82	61.1
11	α-O-α′/β-O-4/γ-OH	I^a^	4.98/–0.3	83.7/+10.9	4.28/+0.2	89.2/–1.9	3.76, 3.82/0	61.4/+0.3
12	α-O-γ′/β-O-4/γ-OH	II^b^	4.98/–0.3	86.3/+13.5	4.28/+0.2	88.9/–2.2	3.76, 3.82/0	61.4/+0.3
13	α-O-γ′/β-O-4/γ-OH	III^c^	4.98/–0.3	86.1/+13.3	4.28/+0.2	89.0/–2.1	3.76, 3.82/0	61.4/+0.3
14	α-OH/β-O-4/γ-O-α′	I^a^	5.28/0	73.1/+0.3	4.28/+0.2	89.2/–1.9	3.61, 3.86/–0.2	66.7/+5.6
15	α-OH/β-O-4/γ-O-γ′	II^b^	5.28/0	73.1/+0.3	4.28/+0.2	88.9/–2.2	3.61, 3.86/–0.2	69.3/+8.2
16	α-OH/β-O-4/γ-O-γ′	III^c^	5.28/0	73.1/+0.3	4.28/+0.2	89.0/–2.1	3.61, 3.86/–0.2	69.1/+8.0

a –α′/β′-O-4/γ′-OH.

b –γ′/β′-O-4/α′-OH.

c –γ′/β′-CH/α′-CH (for structure details, see Figure 5).

d NMR data of reference compound VG.

e Shift difference to reference compound.

Alkylation of γ-OH resulted in a δ_C_ downfield shift of γ-CH of about 5.6–8.2 ppm. Considering the larger downfield shift in the case of etherification through the γ′-position as compared to that through the α′-position, the spectral regions 3 and 4 (Figure 6) were assigned to γ-CH of γ-O-α′/β-O-4 and γ-O-γ′/β-O-4 structures, respectively. These assignments were in line with the above structural identification results based on experimental NMR data.

Of particular interest was the change of the δ_H_/δ_C_ value of β-CH for VG after alkyl etherification (Table 2). Etherification at the α-position or γ-position would cause a downfield shift in the δ_H_ of β-CH (Δδ_H_ = ∼+0.2 ppm) and an upfield shift in δ_C_ (Δδ_C_ = ∼−2 ppm). This was also in agreement with results from experimental NMR data (Table S4). For example, alkyl etherification at the α-CH of VG with an α-alkyl moiety from another side chain would cause a downfield shift in δ_H_ (Δδ_H_ = ∼+0.4 ppm) and an upfield shift in δ_C_ (Δδ_C_ = ∼−6 ppm) for the β-CH. Similarly, alkyl etherification at the α-CH of VG with a γ-alkyl moiety from another side chain would cause a downfield shift (Δδ_H_ = ∼+0.2 ppm) and an upfield shift (Δδ_C_ = ∼−5 ppm) for the β-CH. Accordingly, the effect of alkyl etherification at the α-position or γ-position in the β-O-4 structure on the chemical shifts of the adjacent β-CH was reliably reflected by the simulated NMR data. We can thus deduce that spectral region 2 in Figure 6 will also contain the CH-β resonance of α-O-Alk or γ-O-Alk/β-O-4 ether structures. Spectral region 5 (Figure 6) should be related to the α-CH of epiresinol compounds or similar structures. In addition, etherification of α-OH in VG had very little effect on the chemical shift of its γ-position, and vice versa (Table 2), in agreement with our earlier suggestion.^2^ This is also the reason why the amount of α-OH/β-O-4 structures quantified from the α-CH signal is somewhat higher than that based on the β-CH signal, confirming that some of the α-OH/β-O-4 structures are alkylated at the γ-position.

#### Alk-O-Alk/β-β′Structures

To investigate the chemical shift differences of side-chain positions between bicyclic β-β′ and cyclic β-β′ structures, the cyclic α-O-γ′/β-β′ ether-type model **17**, α-O-α′/β-β′ ether-type model **18**, and γ-O-γ′/β-β′ ether-type model **19** were computationally simulated (Table 3).

**Table 3:** Simulated NMR Data for the Side Chain of Alk-O-Alk/β-β′Ether Models

		α, ppm		β, ppm		γ, ppm	
models	structure description	δ_H_/Δ^b^	δ_C_/Δ	δ_H_/Δ	δ_C_/Δ	δ_H_/Δ	δ_C_/Δ
ref^a^ β–β′	A unit: α-O-γ′/β-β′/γ-O-α′	4.93	86.0	2.33	54.3	3.56, 3.81	71.7
	B unit: α′-O-γ/β′-β/γ′-O-α						
model 17	A unit: α-O-γ′/β-β′/γ-OH	4.93/0	86.3/+0.3	2.13/–0.2	48.1/–6.2	3.33, 3.58/–0.2	60.8/–10.9
	B unit: α′-OH/β′-β/γ′-O-α	4.68/–0.3	83.9/–2.1	2.13/–0.2	51.3/–3.0	3.56, 3.81/0	72.0/+0.3
model 18	A unit: α-O-ά/β-β́/γ-OH	4.93/0	83.4/–2.6	2.13/–0.2	48.1/–6.2	3.33, 3.58/–0.2	60.8/–10.9
	B unit: α′-O-α/β′-β/γ′-OH						
model 19	A unit: α-OH/β-β′/γ-O-γ′	4.68/–0.3	83.9/–2.1	2.13/–0.2	51.3/–2.0	3.56, 3.81/0	62.9/–8.8
	B unit: α′-OH/β′-β/γ′-O-γ						

a Experimental NMR data of reference.

b Shift difference to data from the reference compound.

A comparison of the bicyclic β-β′ resinol structure with the cyclic α-O-Alk/β-β′ ether-type models **17** and **18** (Table 3) showed no change in δ_H_, with a slight downshift of δ_C_ (Δδ_C_ = ∼+0.3 ppm) at the γ′-alkylated α-CH and a more significant upshift for the α′-alkylated structure (Δδ_C_ = ∼−2.6 ppm). Thus, the correlations for α-CH in α-O-α′/β-β′ and α-O-γ′/β-β′ ether structures were attributed to spectral regions 7 and 6, respectively, in Figure 6. Comparison of the cyclic γ-O-Alk/β-β′-type models **17** and **19** with the bicyclic β-β′ resinol structure showed no difference in δ_H_ for their γ-CH, but the corresponding δ_C_ shifted upfield. Combining the above assignment with experimental NMR data in Figure 4, the correlations for γ-CH in cyclic γ-O-α′/β-β′ ether structures were attributed to spectral regions 8 in Figure 6. In addition, the γ-CH correlations in the bicyclic γ-O-α′/β-β′ ether structure (epiresinol) were located in spectral region 3.

The chemical shifts of β-CH in synthesized model compounds **3**, **7**, and **10** could not be evaluated directly for the corresponding nonacetylated moieties because acetylation strongly affects the resonance of β-CH but through simulation studies. There was an upshift in δ_H_/δ_C_ of β-CH in these models relative to the bicyclic β-β′ resinol structure (Table 3). Based on this trend, it was concluded that the region for β-CH of α-O-Alk/β-β′ and γ-O-Alk/β-β′ ether structures should appear around 48–51/2.0–2.2 ppm. However, no significant resonance was observed in this area of the spectrum (Figure 6). This might be due to the much lower response factor of β-CH as compared to that of γ-CH_2_ in the same structures (observed in the lower field, around 65–73/3.5–4.0 ppm) and implied a high structural variety of these moieties with a very minor amount of each specific structure.

In summary, our studies did not only support the earlier tentative assignment of the regions for Alk-O-Alk ethers involved with β-O-4 structures^2^ but also provided information on the chemical shift of various types of moieties involved in β-β′ structures. These substructures were not completely separated in HSQC spectra to be reliably quantified individually. Moreover, some overlapped with the canonical lignin moieties, such as β-O-4/α-OH, pinoresinol, SD, and DBDO. Therefore, Alk-O-Alk moieties can only be quantified as sum (α- and γ-ethers) based on the material balance in ^13^C NMR.^2,3^

### Possible Formation Pathways of Alk-O-Alk Ether Structures in SMWL

#### Radical Coupling Theory

**Scheme 1:**
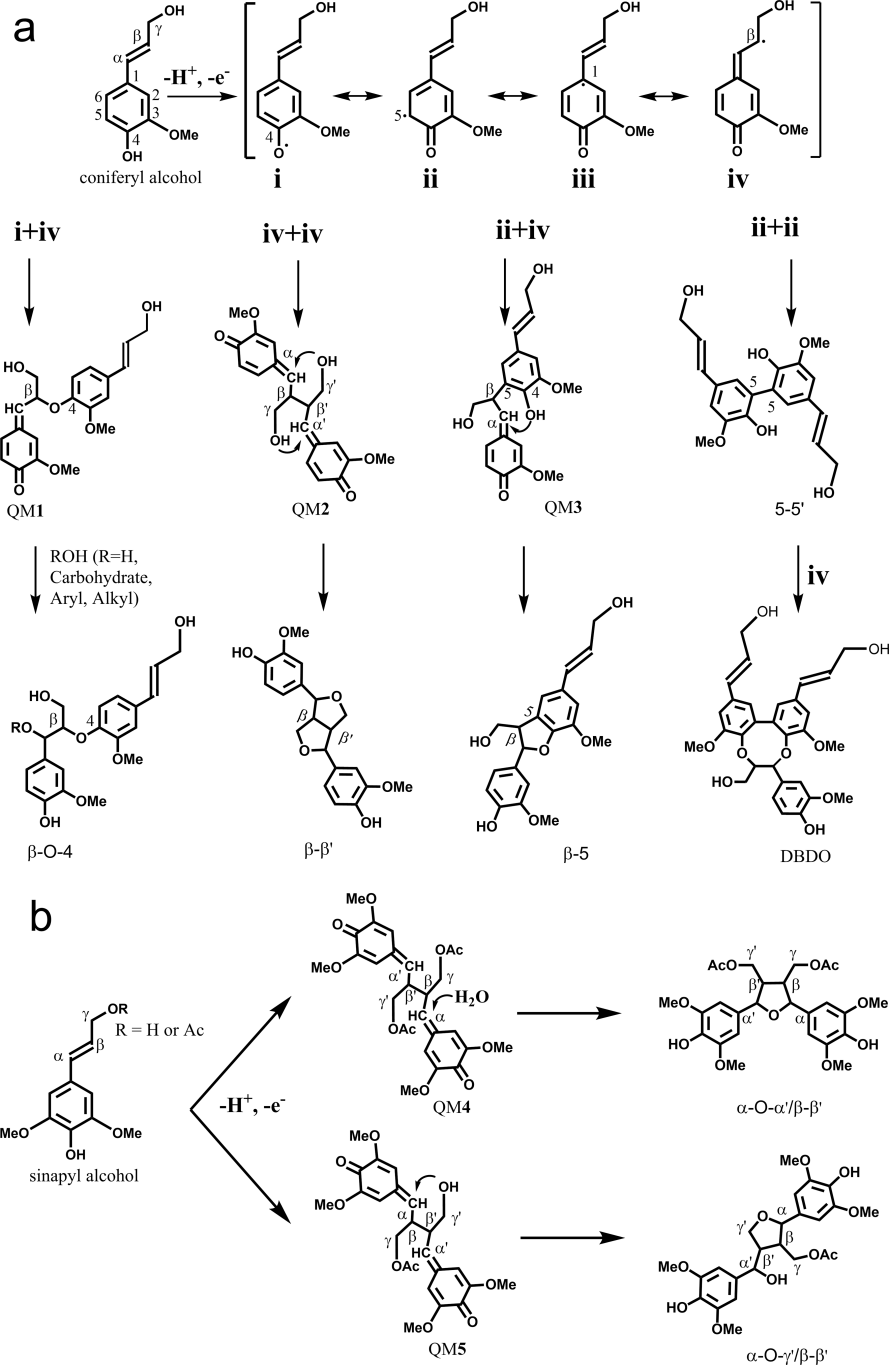
(a) Radical Coupling Theory in Lignin Biosynthesis; (b) Radical β-β Coupling Reactions of γ-Acylated Sinapyl Alcohols or/and Sinapyl Alcohol

According to the generally accepted radical coupling theory^35,36^ in lignin biosynthesis (Scheme 1a), a β-O-4-bonded quinone methide (QM1) could be involved in the formation of a variety of structures, such as α-OH, α-O-carbohydrate, α-O-4, α-O-α′, and α-O-γ′/β-O-4 ethers.^7,27−30^ Brunow and Sipilä *et al.*(28) successfully performed the biomimetic addition of vanillyl alcohol onto a β-O-4-bonded QM dimer. They also synthesized a benzyl alkyl ether (model compound **2**, Figure 3) by the reaction between a QM and dihydroconiferyl alcohol.^29^ Herein, we speculate that QMs generated via the β-O-4 type radical coupling reaction may react with α-OH or γ-OH from another lignin fragment to give rise to a series of Alk-O-Alk/β-O-4 ether structures. However, we could not suggest an appropriate analogous mechanism for the formation of γ-O-γ′/β-O-4 ether structures during lignin biosynthesis so far.

Novel types of β-β′ structures with one opened ring, identified in syringyl lignin from palm, kenaf, and corn cell walls by Ralph and Lu,^11^ also have Alk-O-Alk ether moieties. Unlike the proposed Alk-O-Alk/β-O-4 ether structures, the resinol β-β′structure was produced by internal trapping between the β-β′-bonded QM (QM2 in Scheme 1a) and its two γ-OH. However, if the γ-OH of sinapyl alcohol was acylated, the β-β′ homo-coupling of γ-acylated sinapyl alcohol formed an intermediate bis(quinone methide) (QM4 in Scheme 1b). Since γ-acetylation prevented the internal attack of the γ-OH on QM4, it re-aromatized by water addition, see the typical water addition to QM1. The resultant α-OH intermolecularly added to another QM to form a cyclic α-O-α′/β-β′ether structure with one opened ring.^11^ The β-β′cross-coupling of a γ-acylated monolignol and a typical monolignol produced QM5 (Scheme 1b). There was an internal γ′-OH capable of trapping one QM moiety, forming an α-O-γ′ ether, while the other QM re-aromatized by water addition to form an α-OH, producing an α-O-γ′/β-β′ether structure.^11^ Accordingly, the guaiacyl analogue of Alk-O-Alk/β-β′ ether linkages is proposed to occur similarly to the above formation of the syringyl analogue by the radical coupling mechanism (Scheme 1b).

#### Lignin Aging

**Scheme 2:**
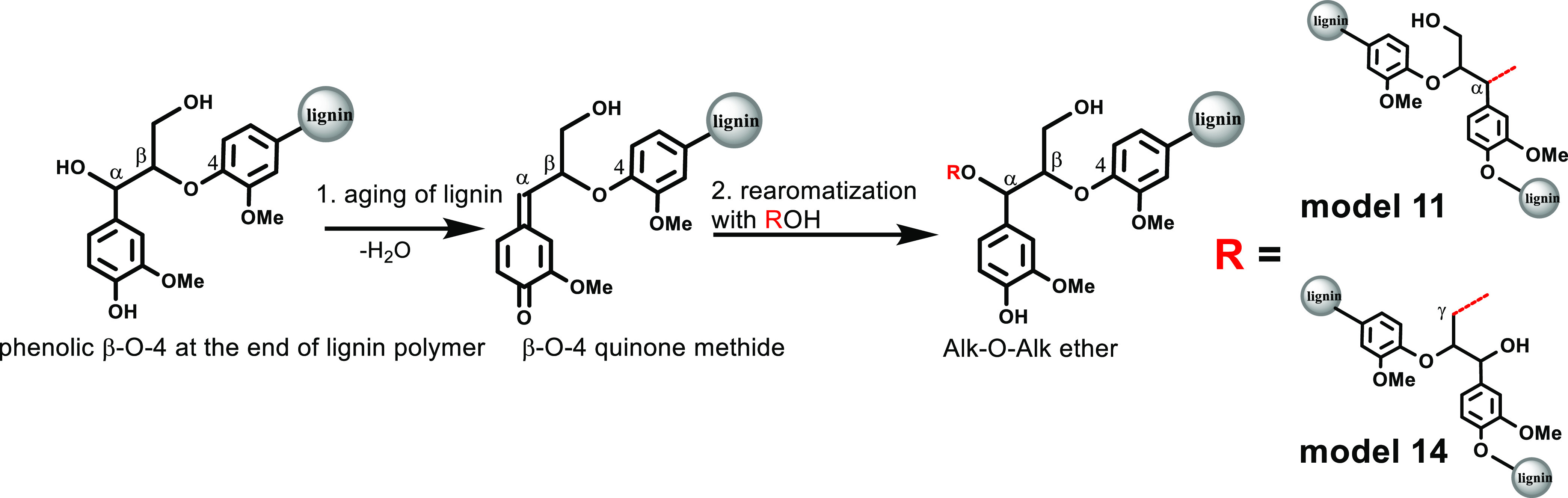
Mechanistic Proposal for the Formation of Alk-O-Alk Ether during Lignin Aging

In addition to the widely accepted dehydrogenation radical coupling mechanism for lignin biosynthesis, there was another proposal that a high number of Alk-O-Alk ether linkages could have been formed during aging of the lignin in plant tissues over the long growing periods. Leary^6^ proposed that there was a potential for QMs to be transiently re-formed throughout the lifetime of the lignin polymer in the plant cell wall based on lignin model compound studies.^37,38^ The reversibility of QM generation was well proven and widely used in biotechniques, such as DNA modification and drug release.^39,40^ This process, therefore, would allow the addition reaction between the regenerated QMs from unstable *p*-hydroxy benzyl alcohol-type structures and lignin aliphatic alcohol nearby, resulting in a considerable amount of structurally variable Alk-O-Alk ether linkages over time (Scheme 2b). It seemed reasonable that the α-O-Alk ethers (see models **11** and **14**) could also be produced during aging of lignin in plants over time.

#### Ball Milling

**Scheme 3:**
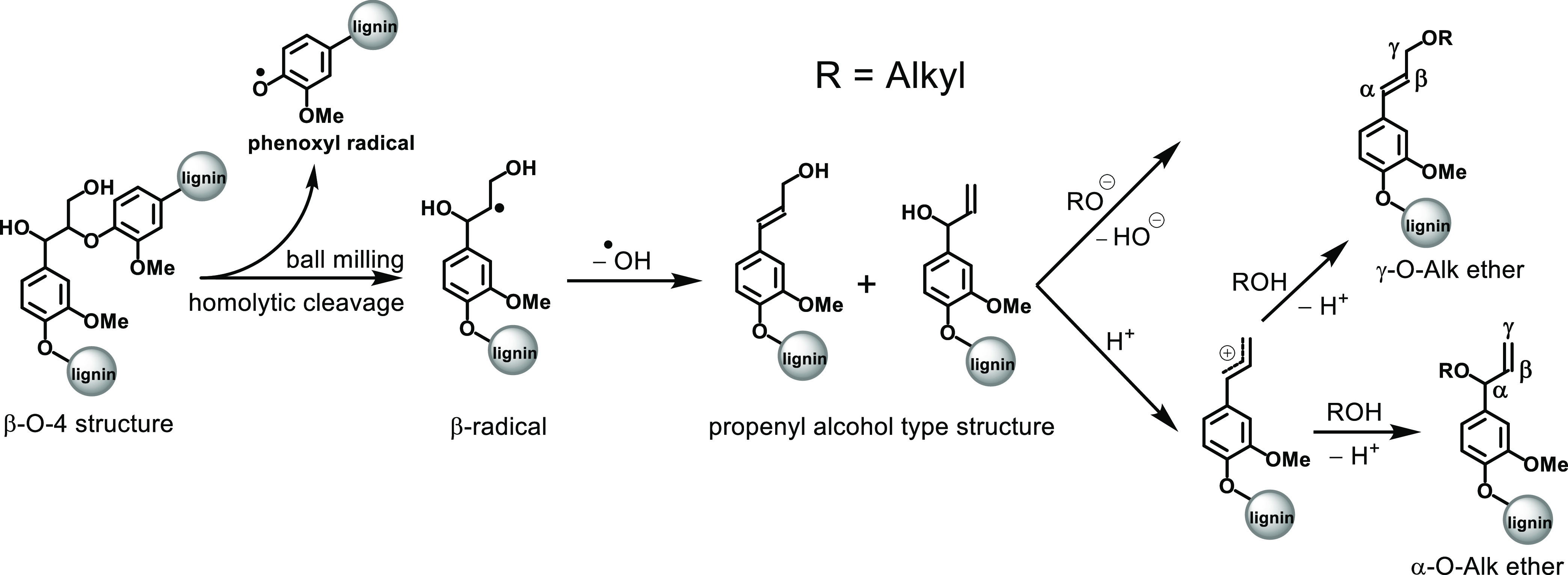
Mechanistic Proposal for the Formation of Alk-O-Alk Ether Structures during Ball Milling

In addition to the possibility of the formation of Alk-O-Alk ether structures during lignin biosynthesis and aging, we also proposed the possibility of their production during ball milling (Scheme 3). According to previous model studies on the mechanochemistry of lignin,^41,42^ structural change of lignin caused by milling proceeded via a radical cleavage of β-O-4 linkages. It is reasonable to assume that recombination and follow-up chemistry of the produced β-radicals can also result in the formation of Alk-O-Alk ether structures during milling. More specifically, the propenyl alcohol-type structures transformed from the β-radicals could generate α- or γ-O-Alk ether structures through a nucleophilic substitution reaction (Scheme 3).

## Summary

The NMR chemical shifts of lignin moieties of Alk-O-Alk ether types were dependent on the substituent type (α-ether or γ-ether) and the type of substituent at the β-position (β-O-4 or β-β′ type). The present study allowed distinguishing between six different types of Alk-O-Alk moieties, specifically those of α-O-α, α-O-γ, and γ-O-γ types with β-O-4 and β-β′ substituents. However, the differences between the specific NMR shift regions were rather subtle, and they were not well separated from each other and from other major lignin moieties. Furthermore, SMWL contained a very high variety of Alk-O-Alk moieties in very minor amounts resulting in superimposed, broad signals. However, although not individually identifiable, the Alk-O-Alk moieties of different types were assigned to different spectral regions in the HSQC spectra and were quantified as the sum parameter (total α- and γ-ethers) based on the material balance in ^13^C NMR spectra. Plausible mechanisms of the formation of various Alk-O-Alk ether structures in lignin biosynthesis, lignin aging, and during ball milling of wood were proposed.

## Supporting Information Available

Carbohydrate content in SMWL, calculated NMR shift data for side chains of Alk-O-Alk/β-O-4 ether model compounds, calculated NMR shift data for side chains of β-β′ and Alk-O-Alk/β-β′ ether model compounds, experimental NMR shift data for the side chain of different reference model compounds, and structural information on synthesized and computational model compounds used in this study (PDF)

## Dedication

In memoriam of Dr. Mikhail Balakshin (1965−2022), who devoted his life to research on lignin.
