# Supplementary material for: Exploring Alkyl-O-Alkyl Ether Structures in Softwood Milled Wood Lignins
Source: J Agric Food Chem. 2022 Dec 21;71(1):580–91. doi: 10.1021/acs.jafc.2c06375 (PMC9837880; doi:10.1021/acs.jafc.2c06375)
Supplement: Supplementary file 1 — jf2c06375_si_001.pdf [file jf2c06375_si_001.pdf]

Supporting Information (SI) for

# Exploring Alkyl-O-Alkyl Ether Structure in Softwood Milled Wood Lignins

Xuhai Zhu[1†*], Jussi Sipilä[2], Antje Potthast[3], Thomas Rosenau[3] and Mikhail Balakshin[1*]

[1]Department of Bioproducts and Biosystems, School of Chemical Engineering, Aalto University, Vuorimiehentie 1, Espoo, 02150, Finland

[2]Laboratory of Organic Material Chemistry, Department of Chemistry, University of Helsinki, P.O 55 (A. I. Virtasen aukio 1), Helsinki, 00014, Finland

[3]Department of Chemistry, Institute for Chemistry of Renewable Resources, University of Natural Resources and Life Sciences (BOKU), Muthgasse 18, 1190, Vienna, Austria

[†]Current address: State Key Laboratory of Catalysis, Dalian National Laboratory for Clean Energy, Dalian Institute of Chemical Physics, Chinese Academy of Sciences, Dalian, Liaoning 110623, P. R. China.

[*]Mikhail Balakshin (1965-2022) -E-mail: mikhail.balakshin@aalto.fi / Mailing address: PO Box 16300, FI-00076 AALTO, Finland / Tel.: +358-(0)50-308-6570

[*]Xuhai Zhu -E-mail: zhuxh@dicp.ac.cn / Mailing address: Dalian Institute of Chemical Physics, CAS, 457 Zhongshan Road Dalian, China 116023 / Tel.: +86-0411-8437-9846

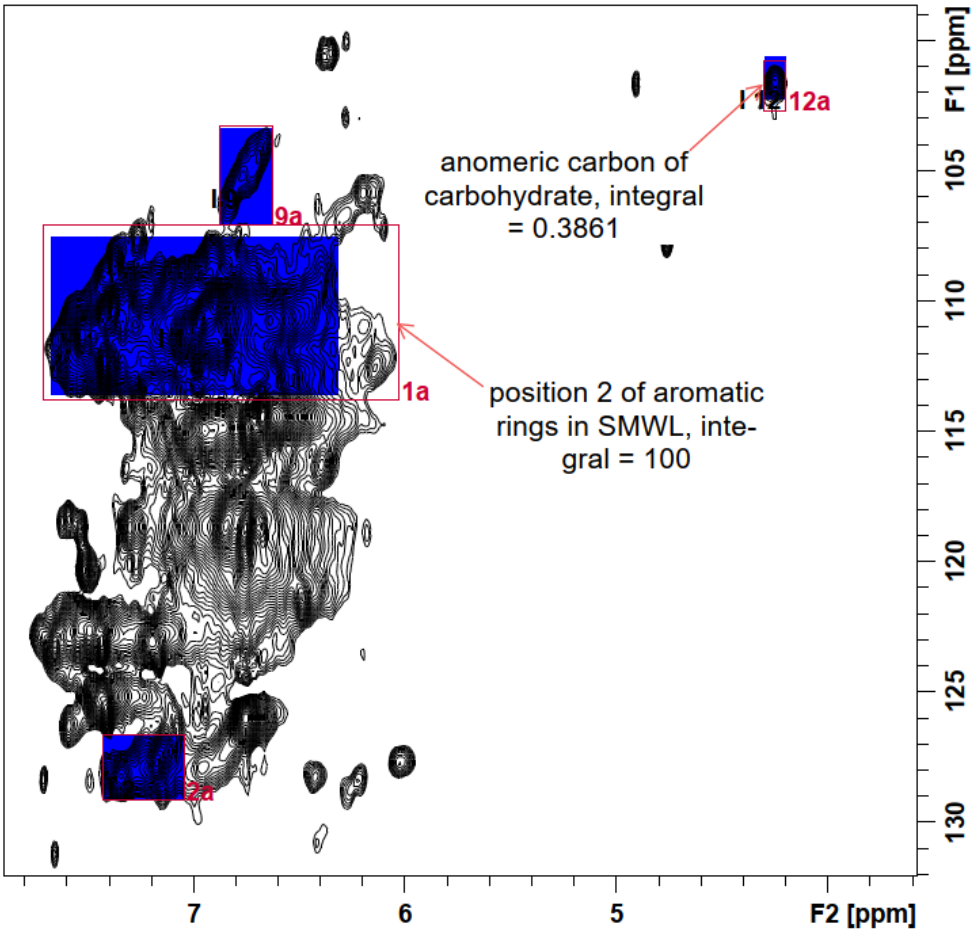


**Figure S1**. Integral of carbohydrate in SMWL (nlev = 50, lev0 = 20, toplev = 100 %)

**Table S1.** Calculated NMR data for the side-chain of Alk-O-Alk/β-O-4 ether model **11–16** (Figure 5)

| Model Information | | | NMR chemical shift on the side chain, $\delta_H/\delta_C$, ppm | | | |
|---|---|---|---|---|---|---|
| Ether type | No. | structure description | $C_9$ unit | α or α′ | β or β′ | γ or γ′ |
| α-O-α′ | **11** | α-O-α′/ β-O-4/γ-OH | A, B | 4.98/83.7 | 4.28/89.2 | 3.76,3.82/61.4 |
| | | α′-O-α/ β′-O-4/γ′-OH | | | | |
| α-O-γ′ | **12** | α-O-γ′/β-O-4/γ-OH | A | 4.98/86.3 | 4.28/88.9 | 3.76,3.82/61.4 |
| | | α′-OH/ β′-O-4/γ′-O-α | B | 5.28/73.1 | 4.28/89.2 | 3.61,3.86/66.7 |
| | **13** | α-O-γ′/β-O-4/γ-OH | A | 4.98/86.1 | 4.28/89.0 | 3.76,3.82/61.4 |
| | | α′-CH/β′-CH/ γ′-O-α | B | 6.65/133.2 | 6.25/126.5 | 4.04/71.7 |
| | **14** | α-OH/ β-O-4/γ-O-α′ | A | 5.28/73.1 | 4.28/89.2 | 3.61,3.86/66.7 |
| | | α′-O-γ/β′-O-4/γ′-OH | B | 4.98/86.3 | 4.28/88.9 | 3.76,3.82/61.4 |
| γ-O-γ′ | **15** | α-OH/β-O-4/γ-O-γ′ | A, B | 5.28/73.1 | 4.28/88.9 | 3.61,3.86/69.3 |
| | | α′-OH/β′-O-4/γ′-O-γ | | | | |
| | **16** | α-OH/β-O-4/γ-O-γ′ | A | 5.28/73.1 | 4.28/89.0 | 3.61,3.86/69.1 |
| | | α′-CH/β′-CH/ γ′-O-γ | B | 6.65/133.2 | 6.25/126.5 | 4.04/74.3 |

**Table S2.** Calculated NMR data for side-chain of β-β′ and Alk-O-Alk/β-β′ ether model **17–19** (Figure 5)

| Model Information | | | NMR chemical shift on the side chain, $\delta_H/\delta_C$, ppm | | | |
|---|---|---|---|---|---|---|
| Ether type | No. | structure description | $C_9$ unit | α or α′ | β or β′ | γ or γ′ |
| bicyclic | β-β′ | α-O-γ′/β-β′/γ-O-α′ | A | 4.93/86.0 | 2.33/54.3 | 3.56,3.81/71.7 |
| | | α′-O-γ/β′-β/γ′-O-α | B | | | |
| α-O-γ′ | **17** | α-O-γ′/β-β′/γ-OH | A | 4.93/86.3 | 2.13/48.1 | 3.33,3.58/60.8 |
| | | α′-OH/β′-β/γ′-O-α | B | 4.68/83.9 | 2.13/51.3 | 3.56,3.81/72.0 |
| α-O-α′ | **18** | α-O-α′/β-β′/γ-OH | A, B | 4.93/83.4 | 2.13/48.1 | 3.33,3.58/60.8 |
| | | α′-O-α/ β′-β/γ′-OH | | | | |
| γ-O-γ′ | **19** | α-OH/β-β′/γ-O-γ′ | A, B | 4.68/83.9 | 2.13/51.3 | 3.56,3.81/62.9 |
| | | α′-OH/β′-β/γ′-O-γ | | | | |

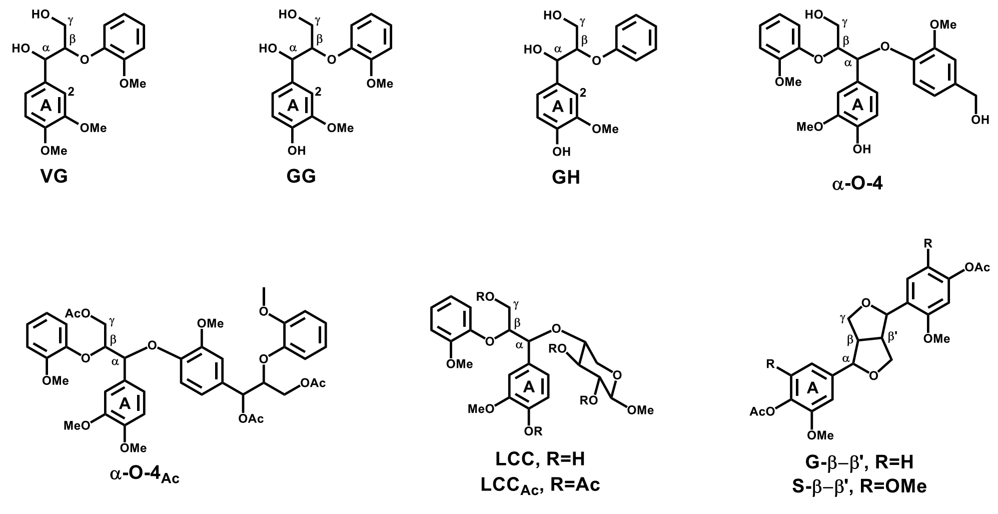


**Figure S2**. Structure of the model compounds corresponding to Table S3

**Table S3.** NMR data for the side chain of different model compounds were used as the reference in this study

| Model Information | | | NMR chemical shift on the side chain, δH/δC, ppm | | |
|---|---|---|---|---|---|
| No. | Structural description | References | α | β | γ |
| VG | veratrolglycerol-β-guaiacyl ether (*erythro* form) or α-OH/β-O-4 /γ-OH | [1][a], in $CDCl_3$ | 4.99/72.8 | 4.17/87.1 | 3.69, 3.92/60.8 |
| | | PC[b] | 5.28/72.8 | 4.08/91.1 | 3.76,3.82/61.1 |
| | | $\Delta\delta_H/\Delta\delta_C$ | Δ0.29/Δ0 | Δ0.09/Δ4.0 | Δ0.07 ~ 0.1/Δ0.3 |
| GG | guaiacylglycerol-β-guaiacyl ether (*threo* form) or α-OH/β-O-4 /γ-OH | [1][a], in $CDCl_3$ | 4.96/74.0 | 4.02/89.5 | 3.65,3.92/61.1 |
| | | PC[b] | 5.28/72.8 | 4.08/91.1 | 3.76,3.82/61.1 |
| | | $\Delta\delta_H/\Delta\delta_C$ | Δ0.32/Δ1.2 | Δ0.06/Δ1.6 | Δ0.11 ~ 0.1/Δ0 |
| GH | guaiacylglycerol-β-*p*-hydroxyphenyl ether (*threo* form) or α-OH/β-O-4 /γ-OH | [2][a], in $CDCl_3$ | 4.95/74.1 | 4.37/83.2 | 3.79.3.57/61.3 |
| | | PC[b] | 5.28/72.8 | 4.08/90.8 | 3.76,3.82/61.1 |
| | | $\Delta\delta_H/\Delta\delta_C$ | Δ0.33/Δ1.3 | Δ0.29/Δ7.6 | Δ0.03 ~ 0.19/Δ0.2 |
| α-O-$4_{Ac}$ | α-O-aryl/β-O-4 /γ-OAc (mixture form) | [1][a], in $CDCl_3$ | 5.35/80.5 | 4.70/82.2 | 4.5-4.7/63.9 |
| α-O-4 | α-O-aryl/β-O-4 /γ-OH (*threo* form) | [1][a], in $CDCl_3$ | 5.36/80.7 | 4.75/81.0 | 4.13, 4.46/65.9 |
| $LCC_{Ac}$ | α-O-glucose/β-O-4 /γ-OAc (*threo* form) | [3][a], in $CDCl_3$ | 4.93/83.4 | 4.58/85.1 | 3.96,4.13/61.4 |
| LCC | α-O-glucose/β-O-4 /γ-OH (*threo* form) | [3][a], in $CDCl_3$ | 4.98/82.6 | 4.36/81.3 | 3.44-3.55,3.74/63.7 |
| G-β-β′ | pinoresinol diacetate | [1][a], in $CDCl_3$ | 4.78/85.5 | 3.08/54.4 | 3.92,4.27/72.0 |
| S-β-β′ | syringylresinol diacetate | [1][a], in $CDCl_3$ | 4.77/85.9 | 3.09/54.4 | 3.95,4.31/72.1 |

[a] from literature; [b] computer simulation by ChemDraw.

**Table S4.** NMR data for side-chain of Alk-O-Alk/β-O-4 ether models

| experimental NMR data | | | | | | | | | |
|---|---|---|---|---|---|---|---|---|---|
| models | references | structure description | | α, ppm | | β, ppm | | γ, ppm | |
| | | A unit | B unit | $\delta_H/\Delta\delta$ | $\delta_C/\Delta\delta$ | $\delta_H/\Delta\delta$ | $\delta_C/\Delta\delta$ | $\delta_H/\Delta\delta$ | $\delta_C/\Delta\delta$ |
| ref.1, **VG** | [1] | **α-OH**/β-O-4/**γ-OH** | - | 4.99 | 72.8 | 4.17 | 87.1 | 3.69, 3.92 | 60.8 |
| **1E** | [4] | **α-O-$CH_3$**/β-O-4/γ-OH | Methyl | 4.44/-0.6 | 83.0/+10.2 | 4.08/-0.1 | 86.9/-0.2 | 3.83,3.92/+0.1 | 61.8/+0.1 |
| **2** | [5] | **α-O-α′**/β-O-4/γ-OH | benzyl | 4.70/-0.3 | 80.1/+7.3 | 4.54/+0.37 | 81.4/-5.7 | 4.07,4.27/+0.3,0.4 | 63.4/+2.6 |
| **6** | [6] | **α-O-γ′**/β-O-4/γ-OAc | II[a] | 4.70/-0.3 | 80.9/+8.1 | 4.5/+0.33 | 81.8/-5.3 | - | - |
| | | α-OAc/β-O-4/**γ-O-α′** | I[b] | - | - | 4.5/+0.33 | 82.0/-5.1 | 3.55/-0.1, 0.4 | 68.2/+7.4 |
| **9E** | [4] | α-OH/β-O-4/**γ-O-$CH_3$** | Methyl | 4.89/-0.1 | 73.1/+0.3 | 4.36/+0.19 | 85.3/-1.8 | 3.45,3.65/-0.2, 0.3 | 71.6+10.8 |

II[a], -γ′/ β′-O-4 / α′-OH; I[b], - α′/ β′-O-4/γ′-OH

## The information on model compounds 1, 4

*Synthesis methods of Alk-O-Alk/β-O-4 ether type model 1, 4*

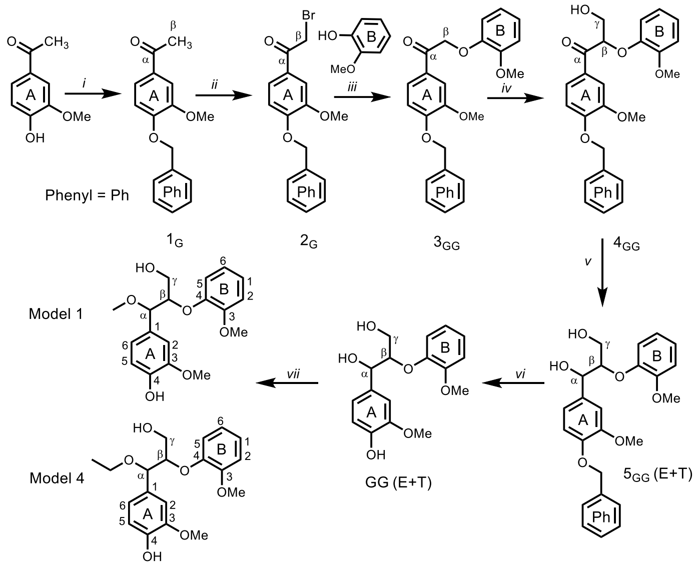


**Scheme S1**. Synthetic pathways of Alk-O-Alk/β-O-4 ether type model 1, 4. Reagents and conditions were as follows: (*i*) BnCl, KI, $K_2CO_3$, anhydrous DMF, 90 °C, (*ii*) $Br_2$, EtOH, rt, (*iii*) $K_2CO_3$, acetone, 40 °C, (*iv*) HCHO, THF, $K_2CO_3$, 40 °C, (v) $NaBH_4$, THF-EtOH (v/v, 1:2), rt (mixture of *erythro* and *threo* isomers), (vi) 10% Pd/C, $H_2$, THF, rt (mixture of *erythro* and *threo* isomers), (vii) TsOH, anhydrous dioxane, MeOH or EtOH, 30 °C (mixture of *erythro* and *threo* isomers).

The synthetic pathways of Alk-O-Alk/β-O-4 ether type models 1, and 4 were shown in Scheme S1. The synthesis of the α-OH/β-O-4 model compound GG from the starting material acetovanillone was according to Adler's method.[7] These experiments were performed in the Wood Chemistry Laboratory of the University of Tokyo during the first author's Ph.D. study. Detailed experimental information can be found in the doctoral dissertation.[8] Subsequently, we synthesized the Alk-O-Alk/β-O-4 ether type model 1, 4 from α-OH/β-O-4 model compound GG according to Adler's method [9] with a small modification. In detail, a stirred solution of anhydrous dioxane (5 mL), compound GG (100 MG, 0.31 mmol, 1 equiv), and *p*-toluenesulfonic acid (TsOH) (570 mg, 3.1 mmol, 10 equiv) charged in a 25 mL of the round bottom flask, anhydrous methanol or ethanol (10 ml) was added and the reaction mixture was kept at 30 $^{o}$C for 24 hours. The progress of the reaction was monitored by TLC [hexane-EtOAc = 1:3]. The reaction was stopped by adding 280 mg of $NaHCO_3$ and stirring for an additional 1 hour. Finally, the product mixture was concentrated under reduced pressure. The resulting residue was then extracted with dichloromethane, washed once with $H_2O$, and once with brine. The organic layer was dried over $Na_2SO_4$ and the solvent was evaporated in vacuo. The target product was purified by the prepared TLC with the developed solvent [hexane-EtOAc = 1:3].

*NMR data for some synthetic or isolated compounds*

The $^{1}H$, $^{13}C$, and DEPT-135 NMR experiments were performed on an NMR sample in DMSO-$d_6$ solvent by a Bruker AVANCE III HD 700MHz spectrometer equipped with a QCI cryogenic probe. All chemical shifts were calibrated by setting the central peak of the residual solvent as $\delta_H$, 2.49, and $\delta_C$, 39.5 ppm. The structural elucidation and the assignment of synthesized compounds were based on the combination of peak integral and previous reports [4, 10].

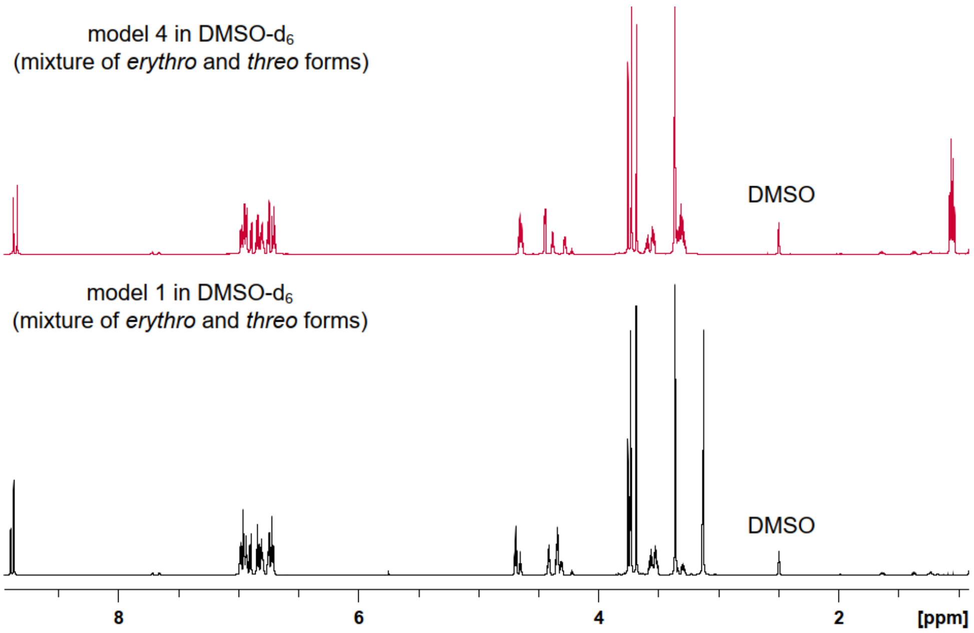


**Figure S3.** $^1$H NMR spectra of Alk-O-Alk/β-O-4 ether type model 1 and 4 in DMSO-d$_6$ (mixture of *erythro* and *threo* isomers)

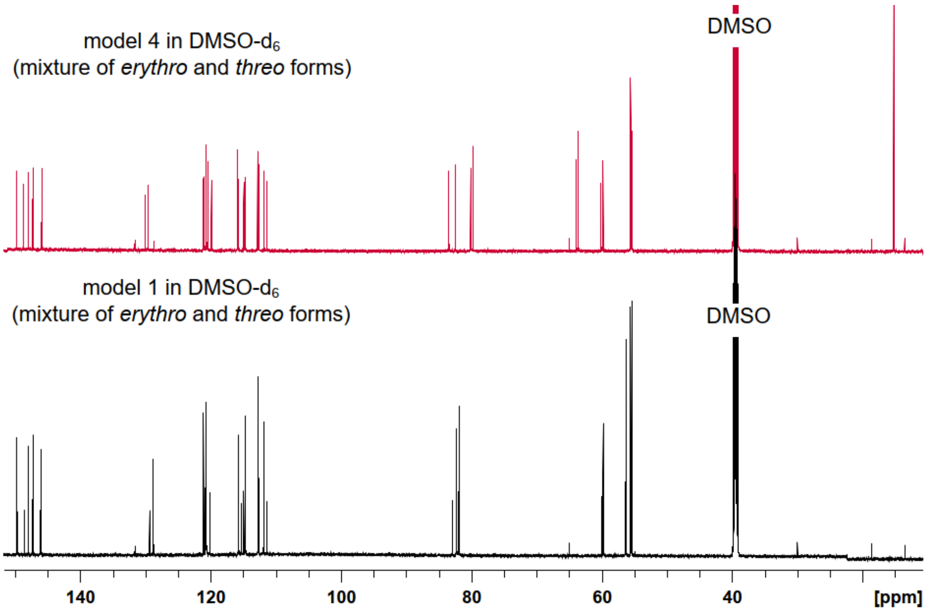


**Figure S4.** $^{13}$C NMR spectra of Alk-O-Alk/β-O-4 ether type model 1 and 4 in DMSO-d$_6$ (mixture of *erythro* and *threo* isomers)

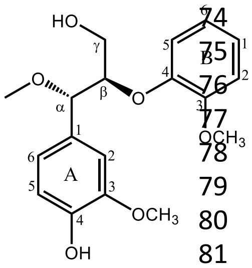


**Model 1** (*erythro* isomer with major amount). $^1$H NMR (DMSO-d$_6$, 700 MHz), $\delta_H$: 3.13 (3H, s, Cα-OC<u>H</u>$_3$), 3.53 (1H, m, $\gamma_1$), 3.56 (1H, m, $\gamma_2$), 3.69 (3H, s, aromatic OC<u>H</u>$_3$), 3.74 (3H, s, aromatic OC<u>H</u>$_3$), 4.34 (1H, broad-d, $J$ = 5 Hz, α), 4.42 (1H, m, β), 4.69 (1H, t, $J$ = 5.6 Hz, γ-O<u>H</u>), 6.70-7.00 (7H, m, aromatic), 8.87 (1H, s, Ph-O<u>H</u>). $^{13}$C NMR (DMSO-d$_6$, 175 MHz), $\delta_C$: 55.4 (aromatic O<u>C</u>H$_3$), 55.6 (aromatic O<u>C</u>H$_3$), 59.8 (Cα-O<u>C</u>H$_3$), 59.8 (<u>C</u>γ), 81.9 (<u>C</u>α), 82.3 (<u>C</u>β), 111.9, 112.7, 114.7, 115.8, 120.7, 120.8 and 121.1 (aromatic, -<u>C</u>H), 128.9, 146.1, 147.3, 148.0 and 149.8 (aromatic, quaternary C).

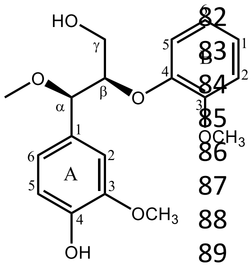


**Model 1** (*threo* isomers with a minor amount). $^1$H NMR (DMSO-d$_6$, 700 MHz), $\delta_H$: 3.14 (3H, s, C$\alpha$-OC$\underline{H}_3$), 3.30 (1H, m, $\gamma_1$), 3.53 (1H, m, $\gamma_2$), 3.73 (3H, s, aromatic OC$\underline{H}_3$), 3.76 (3H, s, aromatic OC$\underline{H}_3$), 4.31 (1H, m, $\beta$), 4.35 (1H, broad-d, $J$ = 5 Hz, $\alpha$), 4.65 (1H, t, $J$ = 5.4 Hz, $\gamma$-O$\underline{H}$), 6.70-7.00 (7H, m, aromatic), 8.90 (1H, s, Ph-O$\underline{H}$). $^{13}$C NMR (DMSO-d$_6$, 175 MHz), $\delta_C$: 55.5 (aromatic O$\underline{C}$H$_3$), 55.6 (aromatic O$\underline{C}$H$_3$), 56.4 (C$\alpha$-O$\underline{C}$H$_3$), 60.1 ($\underline{C}\gamma$), 82.0 ($\underline{C}\alpha$), 83.0 ($\underline{C}\beta$), 111.4, 112.6, 115.0, 115.3, 120.2, 120.7 and 120.9 (aromatic, -$\underline{C}$H), 129.4, 146.1, 147.4, 148.6 and 149.6 (aromatic, quaternary C).

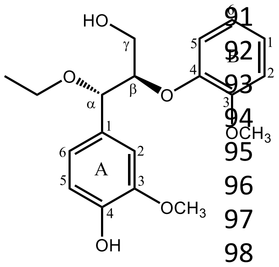


**Model 4** (*erythro* isomer with major amount). $^1$H NMR (DMSO-d$_6$, 700 MHz), $\delta_H$: 1.07 (3H, t, $J$ = 7.1 Hz, C$\alpha$-OCH$_2$C$\underline{H}_3$), 3.32 (2H, m, C$\alpha$-OC$\underline{H}_2$CH$_3$), 3.56 (1H, m, $\gamma_1$), 3.60 (1H, m, $\gamma_2$), 3.69 (3H, s, aromatic OC$\underline{H}_3$), 3.73 (3H, s, aromatic OC$\underline{H}_3$), 4.38 (1H, m, $\beta$), 4.45 (1H, broad-d, $J$ = 5 Hz, $\alpha$), 4.66 (1H, t, $J$ = 5.5 Hz, $\gamma$-O$\underline{H}$), 6.68-7.00 (7H, m, aromatic), 8.85 (1H, s, Ph-O$\underline{H}$). $^{13}$C NMR (DMSO-d$_6$, 175 MHz), $\delta_C$: 15.3 (C$\alpha$-OCH$_2$C$\underline{H}_3$), 55.4 (aromatic O$\underline{C}$H$_3$), 55.7 (aromatic O$\underline{C}$H$_3$), 59.8 ($\underline{C}\gamma$), 63.6 (C$\alpha$-O$\underline{C}$H$_2$CH$_3$), 79.8 ($\underline{C}\alpha$), 82.5 ($\underline{C}\beta$), 111.8, 112.8, 114.7, 115.9, 120.5, 120.7 and 121.1 (aromatic, -$\underline{C}$H), 129.6, 145.9, 147.2, 148.0 and 149.8 (aromatic, quaternary C).

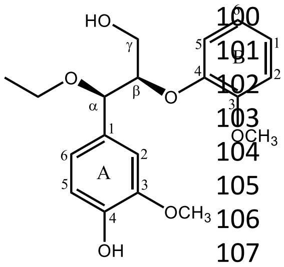


**Model 4** (*threo* isomers with a minor amount). $^1$H NMR (DMSO-d$_6$, 700 MHz), $\delta_H$: 1.05 (3H, t, $J$ = 6.9 Hz, C$\alpha$-OCH$_2$C$\underline{H}_3$), 3.30 (2H, m, C$\alpha$-OC$\underline{H}_2$CH$_3$), 3.32 (1H, m, $\gamma_1$), 3.55 (1H, m, $\gamma_2$), 3.73 (3H, s, aromatic OC$\underline{H}_3$), 3.76 (3H, s, aromatic OC$\underline{H}_3$), 4.28 (1H, m, $\beta$), 4.45 (1H, broad-d, $J$ = 5 Hz, $\alpha$), 4.64 (1H, t, $J$ = 5.4 Hz, $\gamma$-O$\underline{H}$), 6.70-7.00 (7H, m, aromatic), 8.88 (1H, s, Ph-O$\underline{H}$). $^{13}$C NMR (DMSO-d$_6$, 175 MHz), $\delta_C$: 15.3 (C$\alpha$-OCH$_2$C$\underline{H}_3$), 55.5 (aromatic O$\underline{C}$H$_3$), 55.6 (aromatic O$\underline{C}$H$_3$), 60.2 ($\underline{C}\gamma$), 63.9 (C$\alpha$-O$\underline{C}$H$_2$CH$_3$), 80.2 ($\underline{C}\alpha$), 83.5 ($\underline{C}\beta$), 111.4, 112.6, 115.0, 115.8, 120.0, 120.7 and 121.0 (aromatic, -$\underline{C}$H), 130.1, 146.0, 147.3, 148.8 and 149.7 (aromatic, quaternary C).

**The information on model compound 6**

The data for compound 6 was shared by co-author Jussi Sipilä, which was reported as part of a conference paper in 1991.[6]

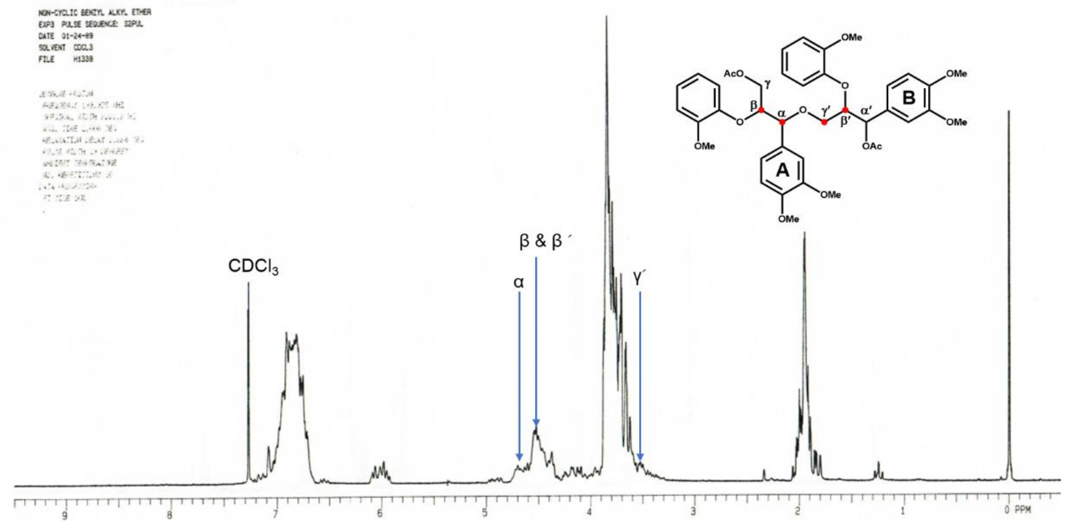


**Figure S5**. $^1$H NMR spectra of model compound 6

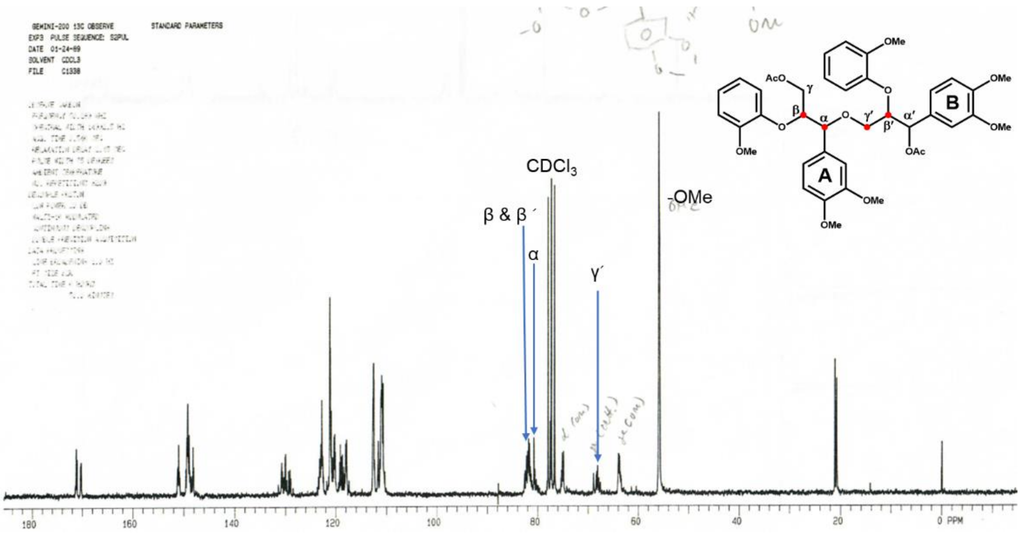


**Figure S6**. $^{13}$C NMR spectra of model compound 6

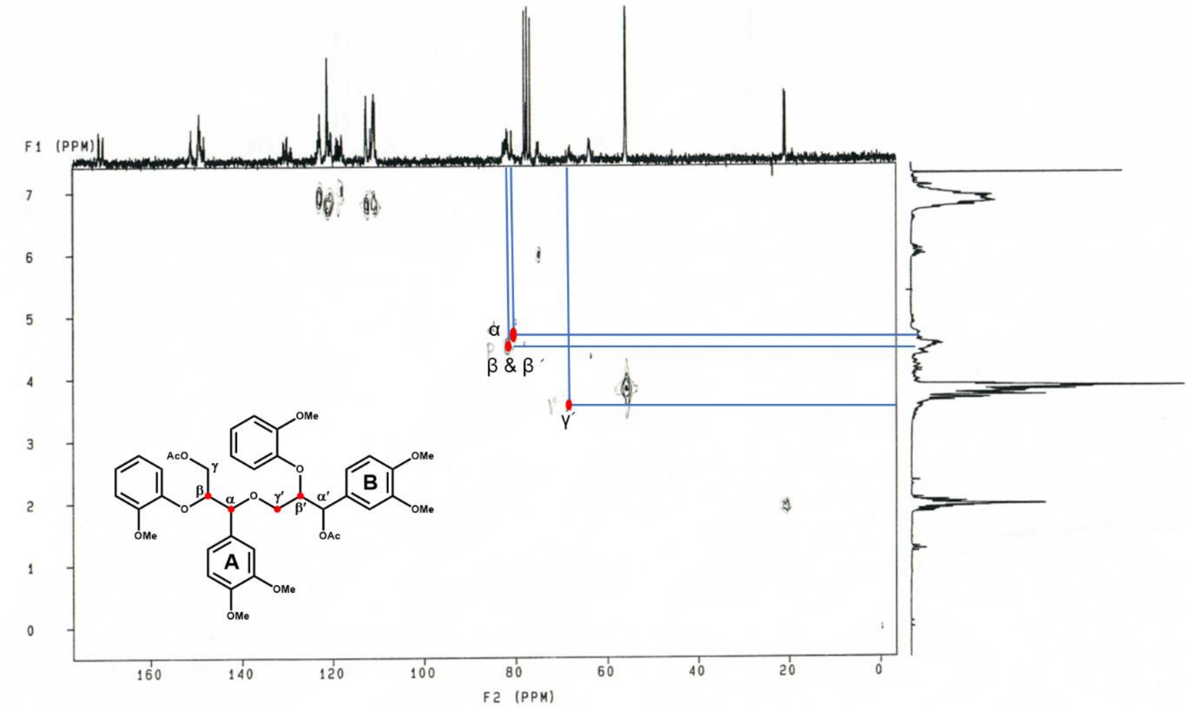


**Figure S7**. HSQC spectra of model compound 6

# The information on NMR calculation of models

ChemNMR $^{1}H$ Estimation

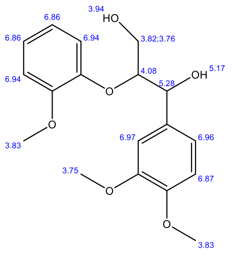


Estimation quality is indicated by color: good, medium, rough

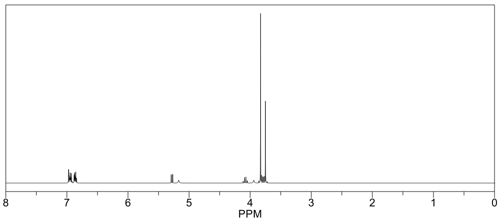


```
Protocol of the H-1 NMR Prediction (Lib=SU Solvent=DMSO 300 MHz):

Node     Shift    Base + Inc.   Comment (ppm rel. to TMS)

OH  5.17              4.20      alcohol
                       1.10        1 -C-C*R
                      -0.13        general corrections
OH  3.94              4.20      alcohol
                       0.20        1 -CC(O)CO
                      -0.46        general corrections
CH  6.94              7.26      1-benzene
                      -0.38        1 -O-C
                       0.00        1 -O-C
                       0.06        general corrections
CH  6.94              7.26      1-benzene
                       0.00        1 -O-C
                      -0.38        1 -O-C
                       0.06        general corrections
CH  6.97              7.26      1-benzene
                      -0.38        1 -O-C
                       0.00        1 -O-C
                       0.00        1 -C-O
                       0.09        general corrections
CH  6.87              7.26      1-benzene
                       0.00        1 -O-C
                      -0.38        1 -O-C
                       0.03        1 -C-O
                      -0.04        general corrections
CH  6.96              7.26      1-benzene
                      -0.32        1 -O-C
                       0.00        1 -O-C
                       0.00        1 -C-O
                       0.02        general corrections
CH  6.86              7.26      1-benzene
                       0.00        1 -O-C
                      -0.32        1 -O-C
                      -0.08        general corrections
CH  6.86              7.26      1-benzene
                      -0.32        1 -O-C
                       0.00        1 -O-C
                      -0.08        general corrections
CH  5.28              1.50      methine
                       1.28        1 alpha -1:C*C*C*C*C*C*1
                       2.10        1 alpha -O
                       0.50        1 beta -O-1:C*C*C*C*C*C*1
                      -0.10        1 beta -C
CH  4.08              1.50      methine
                       2.20        1 alpha -O-1:C*C*C*C*C*C*1
                       0.38        1 beta -1:C*C*C*C*C*C*1
                       0.00        1 beta -O
                       0.00        1 beta -O
CH2 3.82,3.760000      1.37        methylene
                       2.20        1 alpha -O
                       0.38        1 beta -O-1:C*C*C*C*C*C*1
                      -0.06        1 beta -C
                      -0.10        general corrections
CH3 3.75              0.86      methyl
                       2.87        1 alpha -O-1:C*C*C*C*C*C*1
                       0.02        general corrections
CH3 3.83              0.86      methyl
                       2.87        1 alpha -O-1:C*C*C*C*C*C*1
                       0.10        general corrections
CH3 3.83              0.86      methyl
                       2.87        1 alpha -O-1:C*C*C*C*C*C*1
                       0.10        general corrections

1H NMR Coupling Constant Prediction

shift   atom index  coupling partner, constant and vector

5.17        8
3.94       12
6.94       18
                  19   7.5  H-C*C-H
                  20   1.5  H-C*CH*C-H
6.94       21
                  20   7.5  H-C*C-H
                  19   1.5  H-C*CH*C-H
6.97        2
                   6   1.5  H-C*C*C-H
6.87        5
                   6   7.5  H-C*C-H
6.96        6
                   5   7.5  H-C*C-H
                   2   1.5  H-C*C*C-H
6.86       19
                  18   7.5  H-C*C-H
                  20   7.5  H-C*C-H
                  21   1.5  H-C*CH*C-H
6.86       20
                  21   7.5  H-C*C-H
                  19   7.5  H-C*C-H
                  18   1.5  H-C*CH*C-H
5.28        7
                   9   7.0  H-C-C-H
4.08        9
                   7   7.0  H-C-C-H
                  11   7.0  H-C-CH-H
3.79       11 diastereotopic -12.4  H-C-H
                   9   7.0  H-CH-C-H
3.75       16
3.83       17
3.83       24
```

ChemNMR $^{13}C$ Estimation

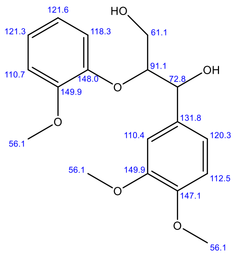


Estimation quality is indicated by color: good, medium, rough

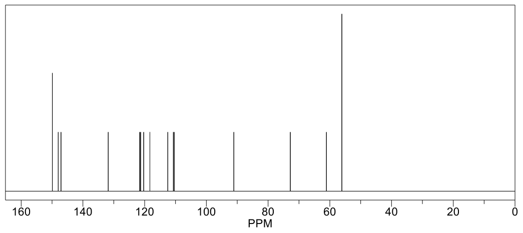


```
Protocol of the C-13 NMR Prediction: (Lib=S)

Node     Shift    Base + Inc.   Comment (ppm rel. to TMS)

C   149.9          128.5      1-benzene
                       33.5      1 -O-C
                      -14.4      1 -O-C
                        0.1      1 -CCC
                        2.2      general corrections
C   147.1          128.5      1-benzene
                      -14.4      1 -O-C
                       33.5      1 -O-C
                       -2.7      1 -CCC
                        2.2      general corrections
C   148.0          128.5      1-benzene
                       30.3      1 -OCC
                      -14.4      1 -O-C
                        3.6      general corrections
C   149.9          128.5      1-benzene
                      -14.3      1 -OCC
                       33.5      1 -O-C
                        2.2      general corrections
C   131.8          128.5      1-benzene
                        1.0      1 -O-C
                       -7.7      1 -O-C
                       10.3      1 -CCC
                       -0.3      general corrections
CH  118.3          128.5      1-benzene
                      -14.3      1 -OCC
                        1.0      1 -O-C
                        3.1      general corrections
CH  110.7          128.5      1-benzene
                        0.6      1 -OCC
                      -14.4      1 -O-C
                       -4.0      general corrections
CH  110.4          128.5      1-benzene
                      -14.4      1 -O-C
                        1.0      1 -O-C
                       -0.2      1 -CCC
                       -4.5      general corrections
CH  112.5          128.5      1-benzene
                        1.0      1 -O-C
                      -14.4      1 -O-C
                        0.1      1 -CCC
                       -2.7      general corrections
CH  120.3          128.5      1-benzene
                       -7.7      1 -O-C
                        1.0      1 -O-C
                       -0.2      1 -CCC
                       -1.3      general corrections
CH  121.6          128.5      1-benzene
                        0.6      1 -OCC
                       -7.7      1 -O-C
                        0.2      general corrections
CH  121.3          128.5      1-benzene
                       -8.4      1 -OCC
                        1.0      1 -O-C
                        0.2      general corrections
CH  72.8            -2.3      aliphatic
                       24.3      1 alpha -1:C*C*C*C*C*C*1
                        9.1      1 alpha -C
                       49.0      1 alpha -O
                        9.4      1 beta -C
                       10.1      1 beta -O
                       -2.6      1 gamma -1:C*C*C*C*C*C*1
                       -6.2      1 gamma -O
                        0.3      1 delta -O
                      -18.3      general corrections
CH  91.1            -2.3      aliphatic
                       18.2      2 alpha -C
                       49.0      1 alpha -O
                       18.6      2 beta -1:C*C*C*C*C*C*1
                       20.2      2 beta -O
                        0.3      1 delta -O
                      -12.9      general corrections
CH2 61.1            -2.3      aliphatic
                        9.1      1 alpha -C
                       49.0      1 alpha -O
                        9.4      1 beta -C
                       10.1      1 beta -O
                       -5.2      2 gamma -1:C*C*C*C*C*C*1
                       -6.2      1 gamma -O
                       -2.8      general corrections
CH3 56.1            -2.3      aliphatic
                       49.0      1 alpha -O
                        9.3      1 beta -1:C*C*C*C*C*C*1
                        0.3      1 delta -O
                       -0.2      general corrections
CH3 56.1            -2.3      aliphatic
                       49.0      1 alpha -O
                        9.3      1 beta -1:C*C*C*C*C*C*1
                        0.3      1 delta -O
                       -0.2      general corrections
CH3 56.1            -2.3      aliphatic
                       49.0      1 alpha -O
                        9.3      1 beta -1:C*C*C*C*C*C*1
                        0.3      1 delta -O
                       -0.2      general corrections
```

**Figure S8**. Calculated NMR spectra of model VG

ChemNMR 1H Estimation

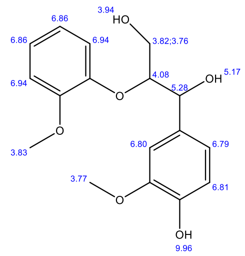


Estimation quality is indicated by color: good, medium, rough

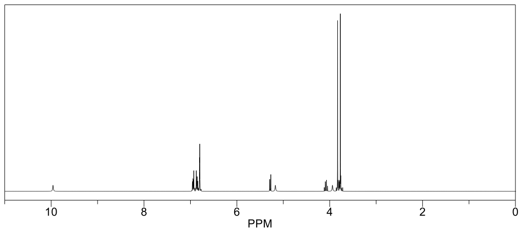


ChemNMR 13C Estimation

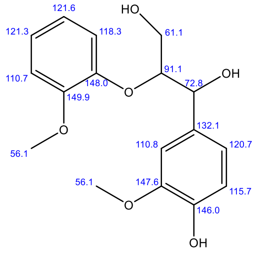


Estimation quality is indicated by color: good, medium, rough

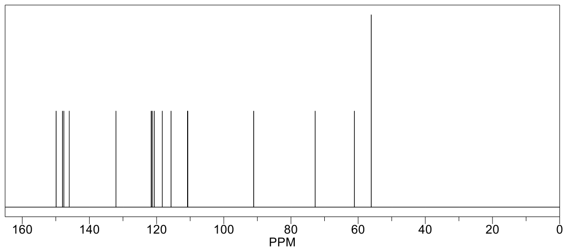


```
Protocol of the H-1 NMR Prediction (Lib=SU Solvent=DMSO 300 MHz):

Node     Shift    Base + Inc.   Comment (ppm rel. to TMS)

OH  9.96              4.20      alcohol
                        4.80       1 -C*R
                        0.96       general corrections
OH  5.17              4.20      alcohol
                        1.10       1 -C-C*R
                       -0.13       general corrections
OH  3.94              4.20      alcohol
                        0.20       1 -CC(O)CO
                       -0.46       general corrections
CH  6.94              7.26      1-benzene
                       -0.38       1 -O-C
                        0.00       1 -O-C
                        0.06       general corrections
CH  6.94              7.26      1-benzene
                        0.00       1 -O-C
                       -0.38       1 -O-C
                        0.06       general corrections
CH  6.80              7.26      1-benzene
                       -0.38       1 -O-C
                       -0.17       1 -O
                        0.00       1 -C-O
                        0.09       general corrections
CH  6.81              7.26      1-benzene
                        0.00       1 -O-C
                       -0.53       1 -O
                        0.03       1 -C-O
                        0.05       general corrections
CH  6.79              7.26      1-benzene
                       -0.32       1 -O-C
                       -0.17       1 -O
                        0.00       1 -C-O
                        0.02       general corrections
CH  6.86              7.26      1-benzene
                        0.00       1 -O-C
                       -0.32       1 -O-C
                       -0.08       general corrections
CH  6.86              7.26      1-benzene
                       -0.32       1 -O-C
                        0.00       1 -O-C
                       -0.08       general corrections
CH  5.28              1.50      methine
                        1.28       1 alpha -1:C*C*C*C*C*C*1
                        2.10       1 alpha -O
                        0.50       1 beta -O-1:C*C*C*C*C*C*1
                       -0.10       1 beta -C
CH  4.08              1.50      methine
                        2.20       1 alpha -O-1:C*C*C*C*C*C*1
                        0.38       1 beta -1:C*C*C*C*C*C*1
                        0.00       1 beta -O
                        0.00       1 beta -O
CH2 3.82,3.760000       1.37       methylene
                        2.20       1 alpha -O
                        0.38       1 beta -O-1:C*C*C*C*C*C*1
                       -0.06       1 beta -C
                       -0.10       general corrections
CH3 3.77              0.86      methyl
                        2.87       1 alpha -O-1:C*C*C*C*C*C*1
                        0.04       general corrections
CH3 3.83              0.86      methyl
                        2.87       1 alpha -O-1:C*C*C*C*C*C*1
                        0.10       general corrections

1H NMR Coupling Constant Prediction

shift   atom index  coupling partner, constant and vector

9.96       14
5.17        8
3.94       12
6.94       17
                 18   7.5  H-C*C-H
                 19   1.5  H-C*CH*C-H
6.94       20
                 19   7.5  H-C*C-H
                 18   1.5  H-C*CH*C-H
6.80        2
                  6   1.5  H-C*C*C-H
6.81        5
                  6   7.5  H-C*C-H
6.79        6
                  5   7.5  H-C*C-H
                  2   1.5  H-C*C*C-H
6.86       18
                 17   7.5  H-C*C-H
                 19   7.5  H-C*C-H
                 20   1.5  H-C*CH*C-H
6.86       19
                 20   7.5  H-C*C-H
                 18   7.5  H-C*C-H
                 17   1.5  H-C*CH*C-H
5.28        7
                  9   7.0  H-C-C-H
4.08        9
                  7   7.0  H-C-C-H
                 11   7.0  H-C-CH-H
3.79       11 diastereotopic -12.4  H-C-H
                  9   7.0  H-CH-C-H
3.77       16
3.83       23
```

```
Protocol of the C-13 NMR Prediction: (Lib=S)

Node     Shift    Base + Inc.   Comment (ppm rel. to TMS)

C   147.6            128.5      1-benzene
                       33.5        1 -O-C
                      -12.8        1 -O
                        0.1        1 -CCC
                       -1.7        general corrections
C   148.0            128.5      1-benzene
                       30.3        1 -OCC
                      -14.4        1 -O-C
                        3.6        general corrections
C   149.9            128.5      1-benzene
                      -14.3        1 -OCC
                       33.5        1 -O-C
                        2.2        general corrections
C   146.0            128.5      1-benzene
                      -14.4        1 -O-C
                       28.8        1 -O
                       -2.7        1 -CCC
                        5.8        general corrections
C   132.1            128.5      1-benzene
                        1.0        1 -O-C
                       -7.4        1 -O
                       10.3        1 -CCC
                       -0.3        general corrections
CH  118.3            128.5      1-benzene
                      -14.3        1 -OCC
                        1.0        1 -O-C
                        3.1        general corrections
CH  110.7            128.5      1-benzene
                        0.6        1 -OCC
                      -14.4        1 -O-C
                       -4.0        general corrections
CH  110.8            128.5      1-benzene
                      -14.4        1 -O-C
                        1.4        1 -O
                       -0.2        1 -CCC
                       -4.5        general corrections
CH  115.7            128.5      1-benzene
                        1.0        1 -O-C
                      -12.8        1 -O
                        0.1        1 -CCC
                       -1.1        general corrections
CH  120.7            128.5      1-benzene
                       -7.7        1 -O-C
                        1.4        1 -O
                       -0.2        1 -CCC
                       -1.3        general corrections
CH  121.6            128.5      1-benzene
                        0.6        1 -OCC
                       -7.7        1 -O-C
                        0.2        general corrections
CH  121.3            128.5      1-benzene
                       -8.4        1 -OCC
                        1.0        1 -O-C
                        0.2        general corrections
CH  72.8              -2.3      aliphatic
                       24.3        1 alpha -1:C*C*C*C*C*C*1
                        9.1        1 alpha -C
                       49.0        1 alpha -O
                        9.4        1 beta -C
                       10.1        1 beta -O
                       -2.6        1 gamma -1:C*C*C*C*C*C*1
                       -6.2        1 gamma -O
                        0.3        1 delta -O
                      -18.3        general corrections
CH  91.1              -2.3      aliphatic
                       18.2        2 alpha -C
                       49.0        1 alpha -O
                       18.6        2 beta -1:C*C*C*C*C*C*1
                       20.2        2 beta -O
                        0.3        1 delta -O
                      -12.9        general corrections
CH2 61.1              -2.3      aliphatic
                        9.1        1 alpha -C
                       49.0        1 alpha -O
                        9.4        1 beta -C
                       10.1        1 beta -O
                       -5.2        2 gamma -1:C*C*C*C*C*C*1
                       -6.2        1 gamma -O
                       -2.8        general corrections
CH3 56.1              -2.3      aliphatic
                       49.0        1 alpha -O
                        9.3        1 beta -1:C*C*C*C*C*C*1
                        0.3        1 delta -O
                       -0.2        general corrections
CH3 56.1              -2.3      aliphatic
                       49.0        1 alpha -O
                        9.3        1 beta -1:C*C*C*C*C*C*1
                        0.3        1 delta -O
                       -0.2        general corrections
```

**Figure S9**. Calculated NMR spectra of model GG

ChemNMR ¹H Estimation

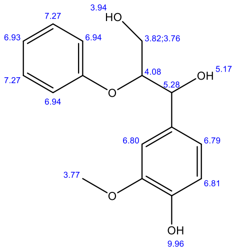


Estimation quality is indicated by color: good, medium, rough

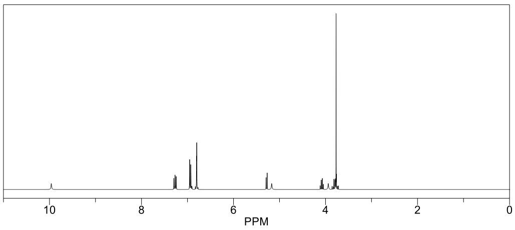


```
Protocol of the H-1 NMR Prediction (Lib=SU Solvent=DMSO 300 MHz):

Node     Shift    Base + Inc.   Comment (ppm rel. to TMS)

OH  9.96               4.20      alcohol
                         4.80       1 -C*R
                         0.96       general corrections
OH  5.17               4.20      alcohol
                         1.10       1 -C-C*R
                        -0.13       general corrections
OH  3.94               4.20      alcohol
                         0.20       1 -CC(O)CO
                        -0.46       general corrections
CH  6.94               7.26      1-benzene
                        -0.38       1 -O-C
                         0.06       general corrections
CH  6.80               7.26      1-benzene
                        -0.38       1 -O-C
                        -0.17       1 -O
                         0.00       1 -C-O
                         0.09       general corrections
CH  6.81               7.26      1-benzene
                         0.00       1 -O-C
                        -0.53       1 -O
                         0.03       1 -C-O
                         0.05       general corrections
CH  6.94               7.26      1-benzene
                        -0.38       1 -O-C
                         0.06       general corrections
CH  6.79               7.26      1-benzene
                        -0.32       1 -O-C
                        -0.17       1 -O
                         0.00       1 -C-O
                         0.02       general corrections
CH  7.27               7.26      1-benzene
                         0.00       1 -O-C
                         0.01       general corrections
CH  7.27               7.26      1-benzene
                         0.00       1 -O-C
                         0.01       general corrections
CH  6.93               7.26      1-benzene
                        -0.32       1 -O-C
                        -0.01       general corrections
CH  5.28               1.50      methine
                         1.28       1 alpha -1:C*C*C*C*C*C*1
                         2.10       1 alpha -O
                         0.50       1 beta -O-1:C*C*C*C*C*C*1
                        -0.10       1 beta -C
CH  4.08               1.50      methine
                         2.20       1 alpha -O-1:C*C*C*C*C*C*1
                         0.38       1 beta -1:C*C*C*C*C*C*1
                         0.00       1 beta -O
                         0.00       1 beta -O
CH2 3.82,3.760000        1.37       methylene
                         2.20       1 alpha -O
                         0.38       1 beta -O-1:C*C*C*C*C*C*1
                        -0.06       1 beta -C
                        -0.10       general corrections
CH3 3.77               0.86      methyl
                         2.87       1 alpha -O-1:C*C*C*C*C*C*1
                         0.04       general corrections

1H NMR Coupling Constant Prediction

shift   atom index  coupling partner, constant and vector

9.96       14
5.17        8
3.94       12
6.94       17
                   18   7.5  H-C*C-H
                   21   1.5  H-C*C*C-H
                   19   1.5  H-C*CH*C-H
6.80        2
                    6   1.5  H-C*C*C-H
6.81        5
                    6   7.5  H-C*C-H
6.94       21
                   20   7.5  H-C*C-H
                   17   1.5  H-C*C*C-H
                   19   1.5  H-C*CH*C-H
6.79        6
                    5   7.5  H-C*C-H
                    2   1.5  H-C*C*C-H
7.27       20
                   21   7.5  H-C*C-H
                   19   7.5  H-C*C-H
                   18   1.5  H-C*CH*C-H
7.27       18
                   17   7.5  H-C*C-H
                   19   7.5  H-C*C-H
                   20   1.5  H-C*CH*C-H
6.93       19
                   20   7.5  H-C*C-H
                   18   7.5  H-C*C-H
                   21   1.5  H-C*CH*C-H
                   17   1.5  H-C*CH*C-H
5.28        7
                    9   7.0  H-C-C-H
4.08        9
                    7   7.0  H-C-C-H
                   11   7.0  H-C-CH-H
3.79       11 diastereotopic -12.4  H-C-H
                    9   7.0  H-CH-C-H
3.77       16
```

ChemNMR ¹³C Estimation

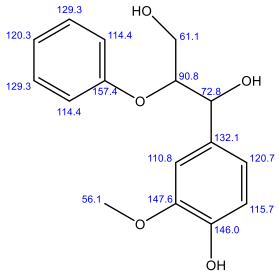


Estimation quality is indicated by color: good, medium, rough

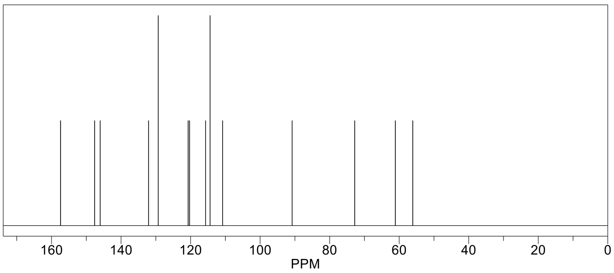


```
Protocol of the C-13 NMR Prediction: (Lib=S)

Node     Shift     Base + Inc.   Comment (ppm rel. to TMS)

C   147.6          128.5      1-benzene
                      33.5       1 -O-C
                     -12.8       1 -O
                       0.1       1 -CCC
                      -1.7       general corrections
C   157.4          128.5      1-benzene
                      30.3       1 -OCC
                      -1.4       general corrections
C   146.0          128.5      1-benzene
                     -14.4       1 -O-C
                      28.8       1 -O
                      -2.7       1 -CCC
                       5.8       general corrections
C   132.1          128.5      1-benzene
                       1.0       1 -O-C
                      -7.4       1 -O
                      10.3       1 -CCC
                      -0.3       general corrections
CH  114.4          128.5      1-benzene
                     -14.3       1 -OCC
                       0.2       general corrections
CH  110.8          128.5      1-benzene
                     -14.4       1 -O-C
                       1.4       1 -O
                      -0.2       1 -CCC
                      -4.5       general corrections
CH  115.7          128.5      1-benzene
                       1.0       1 -O-C
                     -12.8       1 -O
                       0.1       1 -CCC
                      -1.1       general corrections
CH  114.4          128.5      1-benzene
                     -14.3       1 -OCC
                       0.2       general corrections
CH  120.7          128.5      1-benzene
                      -7.7       1 -O-C
                       1.4       1 -O
                      -0.2       1 -CCC
                      -1.3       general corrections
CH  129.3          128.5      1-benzene
                       0.6       1 -OCC
                       0.2       general corrections
CH  129.3          128.5      1-benzene
                       0.6       1 -OCC
                       0.2       general corrections
CH  120.3          128.5      1-benzene
                      -8.4       1 -OCC
                       0.2       general corrections
CH  72.8            -2.3      aliphatic
                      24.3       1 alpha -1:C*C*C*C*C*C*1
                       9.1       1 alpha -C
                      49.0       1 alpha -O
                       9.4       1 beta -C
                      10.1       1 beta -O
                      -2.6       1 gamma -1:C*C*C*C*C*C*1
                      -6.2       1 gamma -O
                       0.3       1 delta -O
                     -18.3       general corrections
CH  90.8            -2.3      aliphatic
                      18.2       2 alpha -C
                      49.0       1 alpha -O
                      18.6       2 beta -1:C*C*C*C*C*C*1
                      20.2       2 beta -O
                     -12.9       general corrections
CH2 61.1            -2.3      aliphatic
                       9.1       1 alpha -C
                      49.0       1 alpha -O
                       9.4       1 beta -C
                      10.1       1 beta -O
                      -5.2       2 gamma -1:C*C*C*C*C*C*1
                      -6.2       1 gamma -O
                      -2.8       general corrections
CH3 56.1            -2.3      aliphatic
                      49.0       1 alpha -O
                       9.3       1 beta -1:C*C*C*C*C*C*1
                       0.3       1 delta -O
                      -0.2       general corrections
```

**Figure S10**. Calculated NMR spectra of model GH

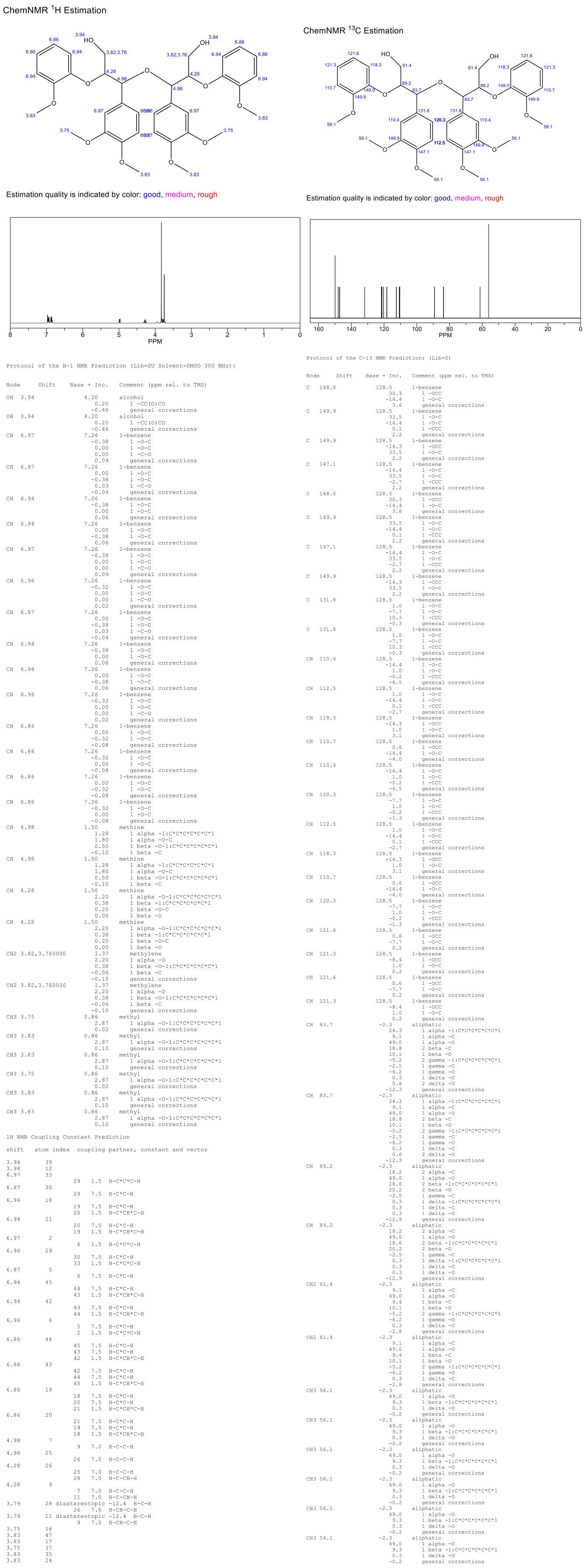


**Figure S11**. Calculated NMR spectra of model 11

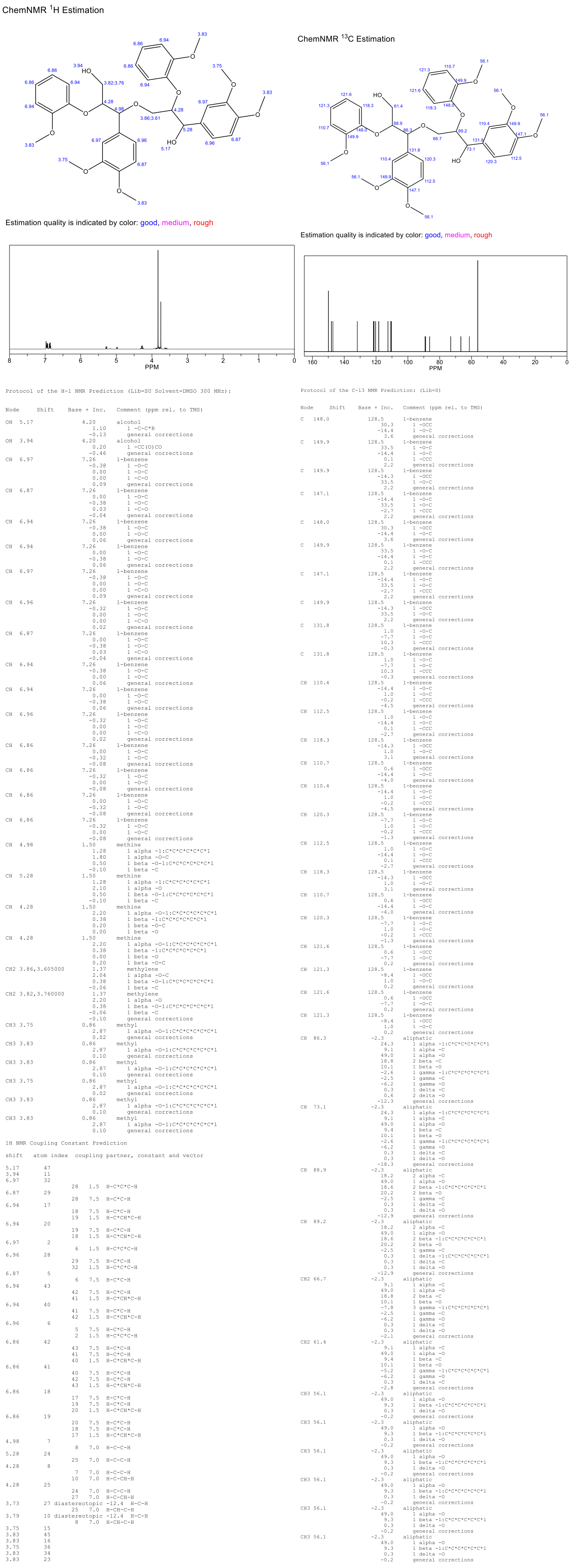


**Figure S12**. Calculated NMR spectra of model 12

ChemNMR $^{1}H$ Estimation

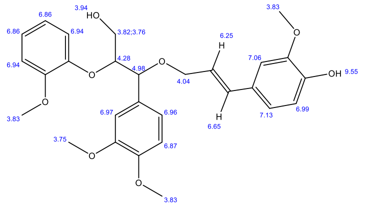

Estimation quality is indicated by color: good, medium, rough

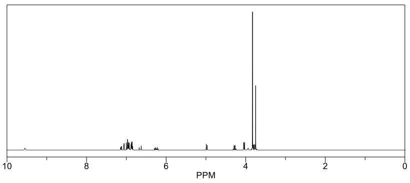


```
Protocol of the H-1 NMR Prediction (Lib=SU Solvent=DMSO 300 MHz):

Node     Shift    Base + Inc.   Comment (ppm rel. to TMS)

OH  9.55              4.20      alcohol
                       4.80        1 -C*R
                       0.55        general corrections
OH  3.94              4.20      alcohol
                       0.20        1 -CC(O)CO
                      -0.46        general corrections
CH  7.06              7.26      1-benzene
                      -0.38        1 -O-C
                      -0.17        1 -O
                       0.04        1 -C=C
                       0.31        general corrections
CH  6.94              7.26      1-benzene
                      -0.38        1 -O-C
                       0.00        1 -O-C
                       0.06        general corrections
CH  6.94              7.26      1-benzene
                       0.00        1 -O-C
                      -0.38        1 -O-C
                       0.06        general corrections
CH  6.99              7.26      1-benzene
                       0.00        1 -O-C
                      -0.53        1 -O
                      -0.05        1 -C=C
                       0.31        general corrections
CH  6.97              7.26      1-benzene
                      -0.38        1 -O-C
                       0.00        1 -O-C
                       0.00        1 -C-O
                       0.09        general corrections
CH  7.13              7.26      1-benzene
                      -0.32        1 -O-C
                      -0.17        1 -O
                       0.04        1 -C=C
                       0.32        general corrections
CH  6.87              7.26      1-benzene
                       0.00        1 -O-C
                      -0.38        1 -O-C
                       0.03        1 -C-O
                      -0.04        general corrections
CH  6.96              7.26      1-benzene
                      -0.32        1 -O-C
                       0.00        1 -O-C
                       0.00        1 -C-O
                       0.02        general corrections
CH  6.86              7.26      1-benzene
                       0.00        1 -O-C
                      -0.32        1 -O-C
                      -0.08        general corrections
CH  6.86              7.26      1-benzene
                      -0.32        1 -O-C
                       0.00        1 -O-C
                      -0.08        general corrections
CH  4.98              1.50      methine
                       1.28        1 alpha -1:C*C*C*C*C*C*1
                       1.80        1 alpha -O-C
                       0.50        1 beta -O-1:C*C*C*C*C*C*1
                      -0.10        1 beta -C
CH  4.28              1.50      methine
                       2.20        1 alpha -O-1:C*C*C*C*C*C*1
                       0.38        1 beta -1:C*C*C*C*C*C*1
                       0.20        1 beta -O-C
                       0.00        1 beta -O
CH2 4.04              1.37      methylene
                       0.63        1 alpha -C=C
                       2.04        1 alpha -O-C
CH2 3.82,3.760000     1.37        methylene
                       2.20        1 alpha -O
                       0.38        1 beta -O-1:C*C*C*C*C*C*1
                      -0.06        1 beta -C
                      -0.10        general corrections
CH3 3.75              0.86      methyl
                       2.87        1 alpha -O-1:C*C*C*C*C*C*1
                       0.02        general corrections
CH3 3.83              0.86      methyl
                       2.87        1 alpha -O-1:C*C*C*C*C*C*1
                       0.10        general corrections
CH3 3.83              0.86      methyl
                       2.87        1 alpha -O-1:C*C*C*C*C*C*1
                       0.10        general corrections
CH3 3.83              0.86      methyl
                       2.87        1 alpha -O-1:C*C*C*C*C*C*1
                       0.10        general corrections
H   6.65              5.25      1-ethylene
                       1.38        1 -1:C*C*C*C*C*C*1 gem
                      -0.01        1 -C-O cis
                       0.03        general corrections
H   6.25              5.25      1-ethylene
                       0.36        1 -1:C*C*C*C*C*C*1 cis
                       0.64        1 -C-O gem

1H NMR Coupling Constant Prediction

shift   atom index  coupling partner, constant and vector

9.55       33
3.94       11
7.06       32
                 28   1.5  H-C*C*C-H
6.94       17
                 18   7.5  H-C*C-H
                 19   1.5  H-C*CH*C-H
6.94       20
                 19   7.5  H-C*C-H
                 18   1.5  H-C*CH*C-H
6.99       29
                 28   7.5  H-C*C-H
6.97        2
                  6   1.5  H-C*C*C-H
7.13       28
                 29   7.5  H-C*C-H
                 32   1.5  H-C*C*C-H
6.87        5
                  6   7.5  H-C*C-H
6.96        6
                  5   7.5  H-C*C-H
                  2   1.5  H-C*C*C-H
6.86       18
                 17   7.5  H-C*C-H
                 19   7.5  H-C*C-H
                 20   1.5  H-C*CH*C-H
6.86       19
                 20   7.5  H-C*C-H
                 18   7.5  H-C*C-H
                 17   1.5  H-C*CH*C-H
4.98        7
                  8   7.0  H-C-C-H
4.28        8
                  7   7.0  H-C-C-H
                 10   7.0  H-C-CH-H
4.04       27
                 38   6.2  H-CH-C(sp2)-H
                 37  -1.0  H-CH>CH=C<H
3.79       10 diastereotopic -12.4  H-C-H
                  8   7.0  H-CH-C-H
3.75       15
3.83       16
3.83       35
3.83       23
6.65       37
                 38  15.1  H>C=C>H
                 27  -1.0  H>C=CH<CH-H
6.25       38
                 27   6.2  H-C(sp2)-CH-H
                 37  15.1  H>C=C>H
```

ChemNMR $^{13}C$ Estimation

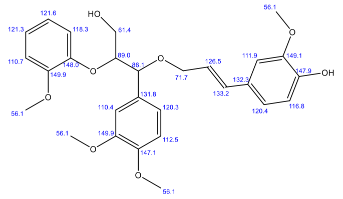

Estimation quality is indicated by color: good, medium, rough

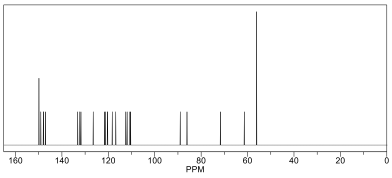


```
Protocol of the C-13 NMR Prediction: (Lib=S)

Node     Shift    Base + Inc.   Comment (ppm rel. to TMS)

C   149.9           128.5      1-benzene
                      33.5        1 -O-C
                     -14.4        1 -O-C
                       0.1        1 -CCC
                       2.2        general corrections
C   147.1           128.5      1-benzene
                     -14.4        1 -O-C
                      33.5        1 -O-C
                      -2.7        1 -CCC
                       2.2        general corrections
C   148.0           128.5      1-benzene
                      30.3        1 -OCC
                     -14.4        1 -O-C
                       3.6        general corrections
C   149.1           128.5      1-benzene
                      33.5        1 -O-C
                     -12.8        1 -O
                      -0.1        1 -C=C
                       0.0        general corrections
C   149.9           128.5      1-benzene
                     -14.3        1 -OCC
                      33.5        1 -O-C
                       2.2        general corrections
C   147.9           128.5      1-benzene
                     -14.4        1 -O-C
                      28.8        1 -O
                      -0.8        1 -C=C
                       5.8        general corrections
C   131.8           128.5      1-benzene
                       1.0        1 -O-C
                      -7.7        1 -O-C
                      10.3        1 -CCC
                      -0.3        general corrections
C   132.3           128.5      1-benzene
                       1.0        1 -O-C
                      -7.4        1 -O
                       6.4        1 -C=C
                       3.8        general corrections
CH  111.9           128.5      1-benzene
                     -14.4        1 -O-C
                       1.4        1 -O
                      -2.3        1 -C=C
                      -1.3        general corrections
CH  118.3           128.5      1-benzene
                     -14.3        1 -OCC
                       1.0        1 -O-C
                       3.1        general corrections
CH  110.7           128.5      1-benzene
                       0.6        1 -OCC
                     -14.4        1 -O-C
                      -4.0        general corrections
CH  116.8           128.5      1-benzene
                       1.0        1 -O-C
                     -12.8        1 -O
                      -0.1        1 -C=C
                       0.2        general corrections
CH  110.4           128.5      1-benzene
                     -14.4        1 -O-C
                       1.0        1 -O-C
                      -0.2        1 -CCC
                      -4.5        general corrections
CH  120.4           128.5      1-benzene
                      -7.7        1 -O-C
                       1.4        1 -O
                      -2.3        1 -C=C
                       0.5        general corrections
CH  112.5           128.5      1-benzene
                       1.0        1 -O-C
                     -14.4        1 -O-C
                       0.1        1 -CCC
                      -2.7        general corrections
CH  120.3           128.5      1-benzene
                      -7.7        1 -O-C
                       1.0        1 -O-C
                      -0.2        1 -CCC
                      -1.3        general corrections
CH  121.6           128.5      1-benzene
                       0.6        1 -OCC
                      -7.7        1 -O-C
                       0.2        general corrections
CH  121.3           128.5      1-benzene
                      -8.4        1 -OCC
                       1.0        1 -O-C
                       0.2        general corrections
CH  86.1             -2.3      aliphatic
                      24.3        1 alpha -1:C*C*C*C*C*C*1
                       9.1        1 alpha -C
                      49.0        1 alpha -O
                      18.8        2 beta -C
                      10.1        1 beta -O
                      -2.6        1 gamma -1:C*C*C*C*C*C*1
                      -2.1        1 gamma -C=C
                      -6.2        1 gamma -O
                       0.3        1 delta -O
                     -12.3        general corrections
CH  89.0             -2.3      aliphatic
                      18.2        2 alpha -C
                      49.0        1 alpha -O
                      18.6        2 beta -1:C*C*C*C*C*C*1
                      20.2        2 beta -O
                      -2.5        1 gamma -C
                       0.4        1 delta -C=C
                       0.3        1 delta -O
                     -12.9        general corrections
CH2 71.7             -2.3      aliphatic
                      19.5        1 alpha -C=C
                      49.0        1 alpha -O
                       9.4        1 beta -C
                      -5.2        2 gamma -1:C*C*C*C*C*C*1
                      -2.5        1 gamma -C
                       0.3        1 delta -C
                       0.3        1 delta -O
                       3.2        general corrections
CH2 61.4             -2.3      aliphatic
                       9.1        1 alpha -C
                      49.0        1 alpha -O
                       9.4        1 beta -C
                      10.1        1 beta -O
                      -5.2        2 gamma -1:C*C*C*C*C*C*1
                      -6.2        1 gamma -O
                       0.3        1 delta -C
                      -2.8        general corrections
CH3 56.1             -2.3      aliphatic
                      49.0        1 alpha -O
                       9.3        1 beta -1:C*C*C*C*C*C*1
                       0.3        1 delta -O
                      -0.2        general corrections
CH3 56.1             -2.3      aliphatic
                      49.0        1 alpha -O
                       9.3        1 beta -1:C*C*C*C*C*C*1
                       0.3        1 delta -O
                      -0.2        general corrections
CH3 56.1             -2.3      aliphatic
                      49.0        1 alpha -O
                       9.3        1 beta -1:C*C*C*C*C*C*1
                       0.3        1 delta -O
                      -0.2        general corrections
CH3 56.1             -2.3      aliphatic
                      49.0        1 alpha -O
                       9.3        1 beta -1:C*C*C*C*C*C*1
                       0.3        1 delta -O
                      -0.2        general corrections
CH  133.2           123.3      1-ethylene
                      12.5        1 -1:C*C*C*C*C*C*1
                      -8.8        1 -C-O-C-C
                       6.2        general corrections
CH  126.5           123.3      1-ethylene
                     -11.0        1 -1:C*C*C*C*C*C*1
                      12.3        1 -C-O-C-C
                       1.9        general corrections
```

**Figure S13**. Calculated NMR spectra of model 13

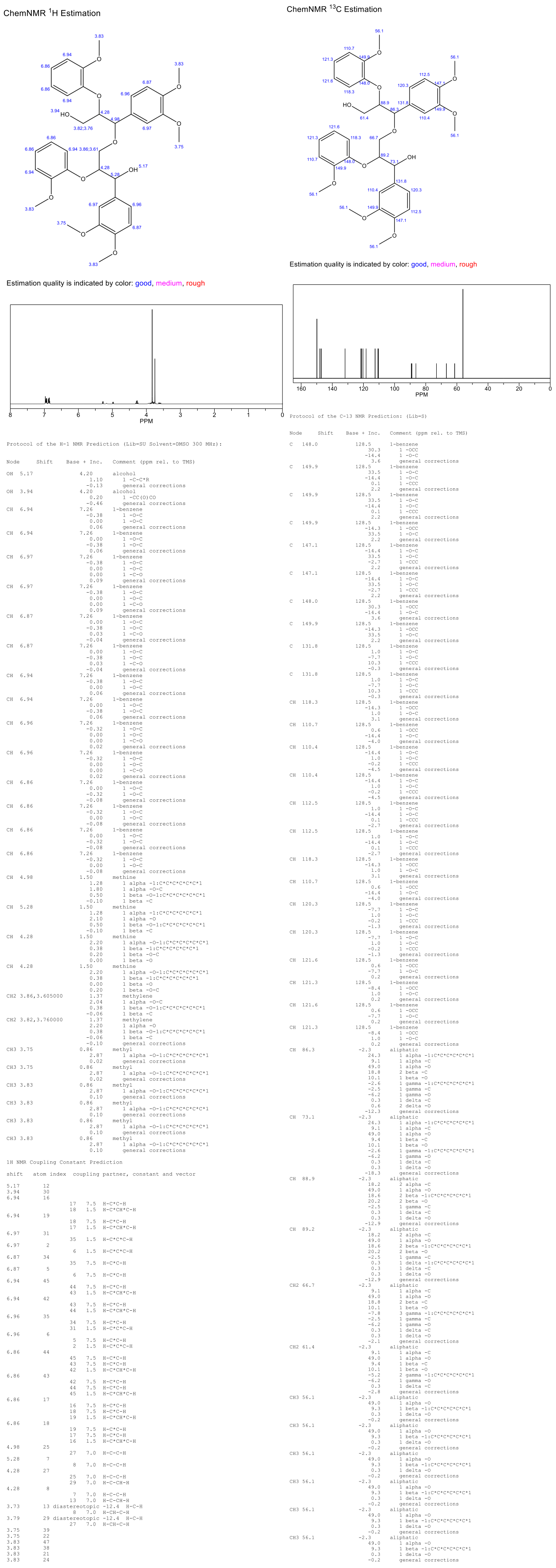


**Figure S14**. Calculated NMR spectra of model 14

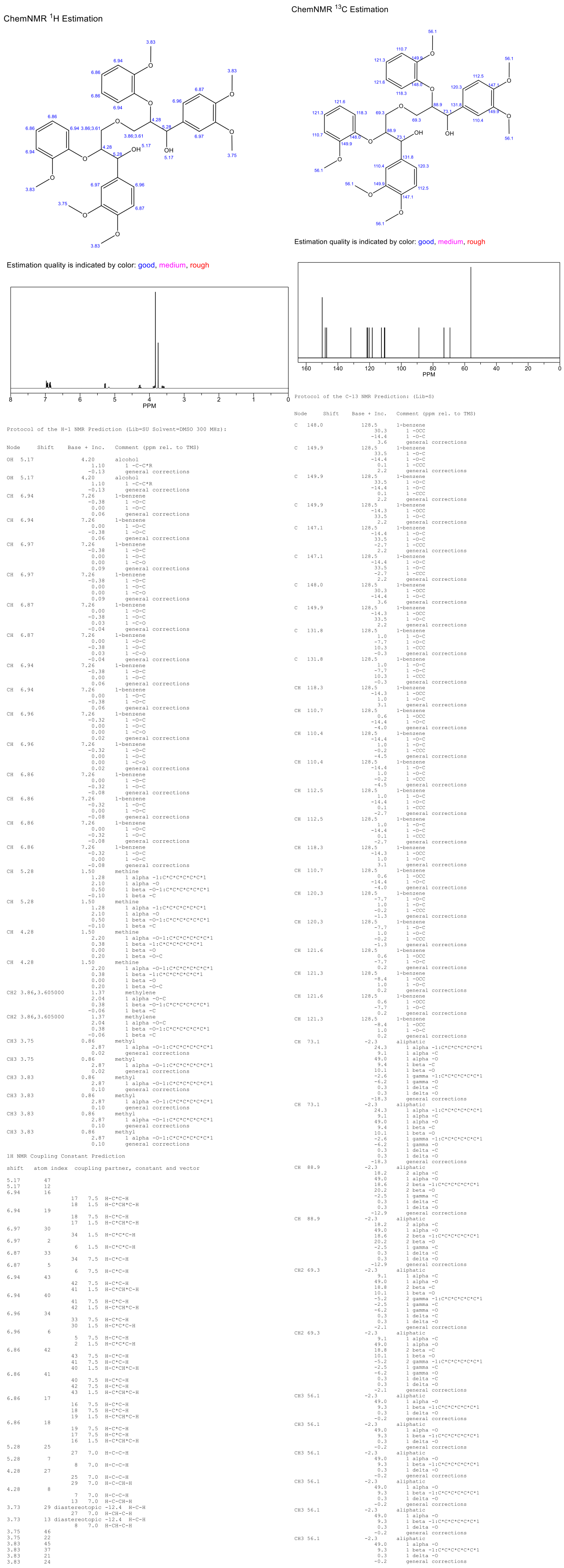


**Figure S15**. Calculated NMR spectra of model 15

ChemNMR $^{1}H$ Estimation

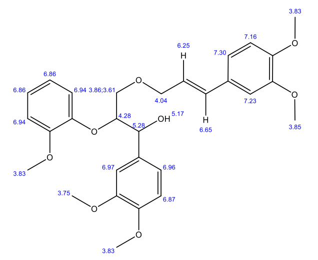


Estimation quality is indicated by color: good, medium, rough

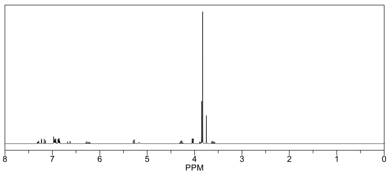


```
Protocol of the H-1 NMR Prediction (Lib=SU Solvent=DMSO 300 MHz):

Node     Shift    Base + Inc.   Comment (ppm rel. to TMS)

OH  5.17               4.20      alcohol
                         1.10       1 -C-C*R
                        -0.13       general corrections
CH  6.94               7.26      1-benzene
                        -0.38       1 -O-C
                         0.00       1 -O-C
                         0.06       general corrections
CH  6.94               7.26      1-benzene
                         0.00       1 -O-C
                        -0.38       1 -O-C
                         0.06       general corrections
CH  6.97               7.26      1-benzene
                        -0.38       1 -O-C
                         0.00       1 -O-C
                         0.00       1 -C-O
                         0.09       general corrections
CH  7.23               7.26      1-benzene
                        -0.38       1 -O-C
                         0.00       1 -O-C
                         0.04       1 -C=C
                         0.31       general corrections
CH  6.87               7.26      1-benzene
                         0.00       1 -O-C
                        -0.38       1 -O-C
                         0.03       1 -C-O
                        -0.04       general corrections
CH  7.16               7.26      1-benzene
                         0.00       1 -O-C
                        -0.38       1 -O-C
                        -0.05       1 -C=C
                         0.33       general corrections
CH  6.96               7.26      1-benzene
                        -0.32       1 -O-C
                         0.00       1 -O-C
                         0.00       1 -C-O
                         0.02       general corrections
CH  7.30               7.26      1-benzene
                        -0.32       1 -O-C
                         0.00       1 -O-C
                         0.04       1 -C=C
                         0.32       general corrections
CH  6.86               7.26      1-benzene
                         0.00       1 -O-C
                        -0.32       1 -O-C
                        -0.08       general corrections
CH  6.86               7.26      1-benzene
                        -0.32       1 -O-C
                         0.00       1 -O-C
                        -0.08       general corrections
CH  5.28               1.50      methine
                         1.28       1 alpha -1:C*C*C*C*C*C*1
                         2.10       1 alpha -O
                         0.50       1 beta -O-1:C*C*C*C*C*C*1
                        -0.10       1 beta -C
CH  4.28               1.50      methine
                         2.20       1 alpha -O-1:C*C*C*C*C*C*1
                         0.38       1 beta -1:C*C*C*C*C*C*1
                         0.00       1 beta -O
                         0.20       1 beta -O-C
CH2 3.86,3.605000      1.37         methylene
                         2.04       1 alpha -O-C
                         0.38       1 beta -O-1:C*C*C*C*C*C*1
                        -0.06       1 beta -C
CH2 4.04               1.37      methylene
                         0.63       1 alpha -C=C
                         2.04       1 alpha -O-C
CH3 3.75               0.86      methyl
                         2.87       1 alpha -O-1:C*C*C*C*C*C*1
                         0.02       general corrections
CH3 3.85               0.86      methyl
                         2.87       1 alpha -O-1:C*C*C*C*C*C*1
                         0.12       general corrections
CH3 3.83               0.86      methyl
                         2.87       1 alpha -O-1:C*C*C*C*C*C*1
                         0.10       general corrections
CH3 3.83               0.86      methyl
                         2.87       1 alpha -O-1:C*C*C*C*C*C*1
                         0.10       general corrections
CH3 3.83               0.86      methyl
                         2.87       1 alpha -O-1:C*C*C*C*C*C*1
                         0.10       general corrections
H   6.65               5.25      1-ethylene
                         1.38       1 -1:C*C*C*C*C*C*1 gem
                        -0.01       1 -C-O cis
                         0.03       general corrections
H   6.25               5.25      1-ethylene
                         0.36       1 -1:C*C*C*C*C*C*1 cis
                         0.64       1 -C-O gem

1H NMR Coupling Constant Prediction

shift   atom index  coupling partner, constant and vector

5.17       12
6.94       16
                 17   7.5  H-C*C-H
                 18   1.5  H-C*CH*C-H
6.94       19
                 18   7.5  H-C*C-H
                 17   1.5  H-C*CH*C-H
6.97        2
                  6   1.5  H-C*C*C-H
7.23       29
                 33   1.5  H-C*C*C-H
6.87        5
                  6   7.5  H-C*C-H
7.16       32
                 33   7.5  H-C*C-H
6.96        6
                  5   7.5  H-C*C-H
                  2   1.5  H-C*C*C-H
7.30       33
                 32   7.5  H-C*C-H
                 29   1.5  H-C*C*C-H
6.86       17
                 16   7.5  H-C*C-H
                 18   7.5  H-C*C-H
                 19   1.5  H-C*CH*C-H
6.86       18
                 19   7.5  H-C*C-H
                 17   7.5  H-C*C-H
                 16   1.5  H-C*CH*C-H
5.28        7
                  8   7.0  H-C-C-H
4.28        8
                  7   7.0  H-C-C-H
                 13   7.0  H-C-CH-H
3.73       13 diastereotopic -12.4  H-C-H
                  8   7.0  H-CH-C-H
4.04       28
                 39   6.2  H-CH-C(sp2)-H
                 38  -1.0  H-CH>CH=C<H
3.75       22
3.85       37
3.83       21
3.83       36
3.83       24
6.65       38
                 39  15.1  H>C=C>H
                 28  -1.0  H>C=CH<CH-H
6.25       39
                 28   6.2  H-C(sp2)-CH-H
                 38  15.1  H>C=C>H
```

ChemNMR $^{13}C$ Estimation

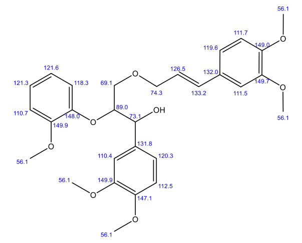


Estimation quality is indicated by color: good, medium, rough

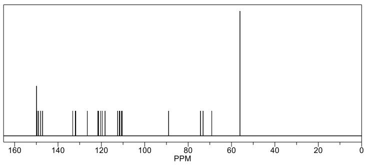


```
Protocol of the C-13 NMR Prediction: (Lib=S)

Node     Shift    Base + Inc.   Comment (ppm rel. to TMS)

C   149.9             128.5      1-benzene
                        33.5        1 -O-C
                       -14.4        1 -O-C
                         0.1        1 -CCC
                         2.2        general corrections
C   149.7             128.5      1-benzene
                        33.5        1 -O-C
                       -14.4        1 -O-C
                        -0.1        1 -C=C
                         2.2        general corrections
C   147.1             128.5      1-benzene
                       -14.4        1 -O-C
                        33.5        1 -O-C
                        -2.7        1 -CCC
                         2.2        general corrections
C   149.0             128.5      1-benzene
                       -14.4        1 -O-C
                        33.5        1 -O-C
                        -0.8        1 -C=C
                         2.2        general corrections
C   148.0             128.5      1-benzene
                        30.3        1 -OCC
                       -14.4        1 -O-C
                         3.6        general corrections
C   149.9             128.5      1-benzene
                       -14.3        1 -OCC
                        33.5        1 -O-C
                         2.2        general corrections
C   131.8             128.5      1-benzene
                         1.0        1 -O-C
                        -7.7        1 -O-C
                        10.3        1 -CCC
                        -0.3        general corrections
C   132.0             128.5      1-benzene
                         1.0        1 -O-C
                        -7.7        1 -O-C
                         6.4        1 -C=C
                         3.8        general corrections
CH  118.3             128.5      1-benzene
                       -14.3        1 -OCC
                         1.0        1 -O-C
                         3.1        general corrections
CH  110.7             128.5      1-benzene
                         0.6        1 -OCC
                       -14.4        1 -O-C
                        -4.0        general corrections
CH  110.4             128.5      1-benzene
                       -14.4        1 -O-C
                         1.0        1 -O-C
                        -0.2        1 -CCC
                        -4.5        general corrections
CH  111.5             128.5      1-benzene
                       -14.4        1 -O-C
                         1.0        1 -O-C
                        -2.3        1 -C=C
                        -1.3        general corrections
CH  112.5             128.5      1-benzene
                         1.0        1 -O-C
                       -14.4        1 -O-C
                         0.1        1 -CCC
                        -2.7        general corrections
CH  111.7             128.5      1-benzene
                         1.0        1 -O-C
                       -14.4        1 -O-C
                        -0.1        1 -C=C
                        -3.3        general corrections
CH  120.3             128.5      1-benzene
                        -7.7        1 -O-C
                         1.0        1 -O-C
                        -0.2        1 -CCC
                        -1.3        general corrections
CH  119.6             128.5      1-benzene
                        -7.7        1 -O-C
                         1.0        1 -O-C
                        -2.3        1 -C=C
                         0.1        general corrections
CH  121.6             128.5      1-benzene
                         0.6        1 -OCC
                        -7.7        1 -O-C
                         0.2        general corrections
CH  121.3             128.5      1-benzene
                        -8.4        1 -OCC
                         1.0        1 -O-C
                         0.2        general corrections
CH  73.1               -2.3      aliphatic
                        24.3        1 alpha -1:C*C*C*C*C*C*1
                         9.1        1 alpha -C
                        49.0        1 alpha -O
                         9.4        1 beta -C
                        10.1        1 beta -O
                        -2.6        1 gamma -1:C*C*C*C*C*C*1
                        -6.2        1 gamma -O
                         0.3        1 delta -C
                         0.3        1 delta -O
                       -18.3        general corrections
CH  89.0               -2.3      aliphatic
                        18.2        2 alpha -C
                        49.0        1 alpha -O
                        18.6        2 beta -1:C*C*C*C*C*C*1
                        20.2        2 beta -O
                        -2.5        1 gamma -C
                         0.4        1 delta -C=C
                         0.3        1 delta -O
                       -12.9        general corrections
CH2 69.1               -2.3      aliphatic
                         9.1        1 alpha -C
                        49.0        1 alpha -O
                        18.8        2 beta -C
                        10.1        1 beta -O
                        -5.2        2 gamma -1:C*C*C*C*C*C*1
                        -2.1        1 gamma -C=C
                        -6.2        1 gamma -O
                        -2.1        general corrections
CH2 74.3               -2.3      aliphatic
                        19.5        1 alpha -C=C
                        49.0        1 alpha -O
                         9.4        1 beta -C
                        -2.6        1 gamma -1:C*C*C*C*C*C*1
                        -2.5        1 gamma -C
                         0.3        1 delta -C
                         0.3        1 delta -O
                         3.2        general corrections
CH3 56.1               -2.3      aliphatic
                        49.0        1 alpha -O
                         9.3        1 beta -1:C*C*C*C*C*C*1
                         0.3        1 delta -O
                        -0.2        general corrections
CH3 56.1               -2.3      aliphatic
                        49.0        1 alpha -O
                         9.3        1 beta -1:C*C*C*C*C*C*1
                         0.3        1 delta -O
                        -0.2        general corrections
CH3 56.1               -2.3      aliphatic
                        49.0        1 alpha -O
                         9.3        1 beta -1:C*C*C*C*C*C*1
                         0.3        1 delta -O
                        -0.2        general corrections
CH3 56.1               -2.3      aliphatic
                        49.0        1 alpha -O
                         9.3        1 beta -1:C*C*C*C*C*C*1
                         0.3        1 delta -O
                        -0.2        general corrections
CH3 56.1               -2.3      aliphatic
                        49.0        1 alpha -O
                         9.3        1 beta -1:C*C*C*C*C*C*1
                         0.3        1 delta -O
                        -0.2        general corrections
CH  133.2             123.3      1-ethylene
                        12.5        1 -1:C*C*C*C*C*C*1
                        -8.8        1 -C-O-C-C
                         6.2        general corrections
CH  126.5             123.3      1-ethylene
                       -11.0        1 -1:C*C*C*C*C*C*1
                        12.3        1 -C-O-C-C
                         1.9        general corrections
```

**Figure S16**. Calculated NMR spectra of model 16

ChemNMR $^{1}$H Estimation

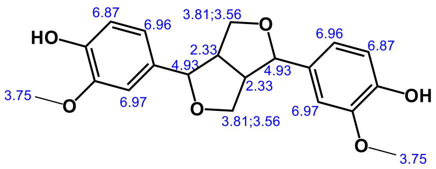


Estimation quality is indicated by color: good, medium, rough

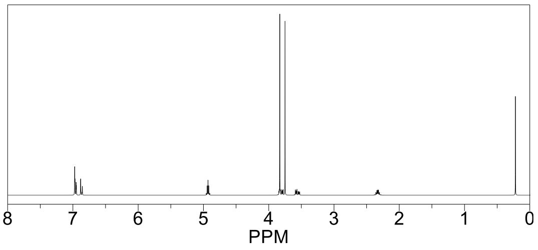


```
Protocol of the H-1 NMR Prediction (Lib=SU Solvent=DMSO 300 MHz):

Node     Shift    Base + Inc.   Comment (ppm rel. to TMS)

CH  4.93              3.75      tetrahydrofuran
                        1.28       1 alpha -1:C*C*C*C*C*C*1 from methine
                       -0.10       1 beta -C from methine
CH  4.93              3.75      tetrahydrofuran
                        1.28       1 alpha -1:C*C*C*C*C*C*1 from methine
                       -0.10       1 beta -C from methine
CH2 3.81,3.565000       3.75       tetrahydrofuran
                       -0.06       1 beta -C from methylene
CH2 3.81,3.565000       3.75       tetrahydrofuran
                       -0.06       1 beta -C from methylene
CH  2.33              1.85      tetrahydrofuran
                        0.38       1 beta -1:C*C*C*C*C*C*1 from methine
                        0.20       1 beta -O-C from methine
                       -0.10       1 beta -C from methine
CH  2.33              1.85      tetrahydrofuran
                        0.38       1 beta -1:C*C*C*C*C*C*1 from methine
                        0.20       1 beta -O-C from methine
                       -0.10       1 beta -C from methine
CH  6.97              7.26      1-benzene
                       -0.38       1 -O-C
                        0.00       1 -O-C
                        0.00       1 -C-O
                        0.09       general corrections
CH  6.97              7.26      1-benzene
                       -0.38       1 -O-C
                        0.00       1 -O-C
                        0.00       1 -C-O
                        0.09       general corrections
CH  6.87              7.26      1-benzene
                        0.00       1 -O-C
                       -0.38       1 -O-C
                        0.03       1 -C-O
                       -0.04       general corrections
CH  6.87              7.26      1-benzene
                        0.00       1 -O-C
                       -0.38       1 -O-C
                        0.03       1 -C-O
                       -0.04       general corrections
CH  6.96              7.26      1-benzene
                       -0.32       1 -O-C
                        0.00       1 -O-C
                        0.00       1 -C-O
                        0.02       general corrections
CH  6.96              7.26      1-benzene
                       -0.32       1 -O-C
                        0.00       1 -O-C
                        0.00       1 -C-O
                        0.02       general corrections
CH3 3.75              0.86      methyl
                        2.87       1 alpha -O-1:C*C*C*C*C*C*1
                        0.02       general corrections
CH3 3.75              0.86      methyl
                        2.87       1 alpha -O-1:C*C*C*C*C*C*1
                        0.02       general corrections
CH3 3.83              0.86      methyl
                        2.87       1 alpha -O-1:C*C*C*C*C*C*1
                        0.10       general corrections
CH3 3.83              0.86      methyl
                        2.87       1 alpha -O-1:C*C*C*C*C*C*1
                        0.10       general corrections
CH4 0.22              0.23      methane
                       -0.01       general corrections

1H NMR Coupling Constant Prediction

shift   atom index  coupling partner, constant and vector

4.93        7
                  11   7.0  H-C-C-H
4.93       12
                  10   7.0  H-C-C-H
3.69        9 diastereotopic -12.4  H-C-H
                  10   7.0  H-CH-C-H
3.69       13 diastereotopic -12.4  H-C-H
                  11   7.0  H-CH-C-H
2.33       10
                  12   7.0  H-C-C-H
                   9   7.0  H-C-CH-H
                  11   7.0  H-C-C-H
2.33       11
                   7   7.0  H-C-C-H
                  13   7.0  H-C-CH-H
                  10   7.0  H-C-C-H
6.97        5
                   1   1.5  H-C*C*C-H
6.97       15
                  19   1.5  H-C*C*C-H
6.87        2
                   1   7.5  H-C*C-H
6.87       18
                  19   7.5  H-C*C-H
6.96        1
                   2   7.5  H-C*C-H
                   5   1.5  H-C*C*C-H
6.96       19
                  18   7.5  H-C*C-H
                  15   1.5  H-C*C*C-H
3.75       28
3.75       29
3.83       25
3.83       26
0.22       27
```

ChemNMR $^{13}$C Estimation

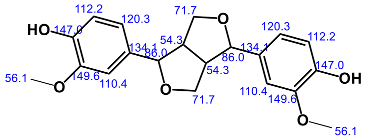


Estimation quality is indicated by color: good, medium, rough

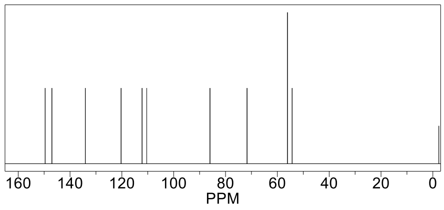


```
Protocol of the C-13 NMR Prediction: (Lib=S)

Node     Shift    Base + Inc.   Comment (ppm rel. to TMS)

CH  86.0             -8.5      tetrahydrofuran
                       24.3       1 alpha -1:C*C*C*C*C*C*1 from aliphatic
                        9.1       1 alpha -C from aliphatic
                       49.0       1 alpha -O from aliphatic
                       28.2       3 beta -C from aliphatic
                       -2.5       1 gamma -C from aliphatic
                       -6.2       1 gamma -O from aliphatic
                        0.3       1 delta -1:C*C*C*C*C*C*1 from aliphatic
                        0.3       1 delta -O from aliphatic
                       -8.0       general corrections
CH  86.0             -8.5      tetrahydrofuran
                       24.3       1 alpha -1:C*C*C*C*C*C*1 from aliphatic
                        9.1       1 alpha -C from aliphatic
                       49.0       1 alpha -O from aliphatic
                       28.2       3 beta -C from aliphatic
                       -2.5       1 gamma -C from aliphatic
                       -6.2       1 gamma -O from aliphatic
                        0.3       1 delta -1:C*C*C*C*C*C*1 from aliphatic
                        0.3       1 delta -O from aliphatic
                       -8.0       general corrections
CH2 71.7             -8.5      tetrahydrofuran
                        9.1       1 alpha -C from aliphatic
                       49.0       1 alpha -O from aliphatic
                       28.2       3 beta -C from aliphatic
                       -5.2       2 gamma -1:C*C*C*C*C*C*1 from aliphatic
                       -2.5       1 gamma -C from aliphatic
                       -6.2       1 gamma -O from aliphatic
                        7.8       general corrections
CH2 71.7             -8.5      tetrahydrofuran
                        9.1       1 alpha -C from aliphatic
                       49.0       1 alpha -O from aliphatic
                       28.2       3 beta -C from aliphatic
                       -5.2       2 gamma -1:C*C*C*C*C*C*1 from aliphatic
                       -2.5       1 gamma -C from aliphatic
                       -6.2       1 gamma -O from aliphatic
                        7.8       general corrections
CH  54.3            -11.2      tetrahydrofuran
                       27.3       3 alpha -C from aliphatic
                        9.3       1 beta -1:C*C*C*C*C*C*1 from aliphatic
                       18.8       2 beta -C from aliphatic
                       20.2       2 beta -O from aliphatic
                       -2.6       1 gamma -1:C*C*C*C*C*C*1 from aliphatic
                       -7.5       general corrections
CH  54.3            -11.2      tetrahydrofuran
                       27.3       3 alpha -C from aliphatic
                        9.3       1 beta -1:C*C*C*C*C*C*1 from aliphatic
                       18.8       2 beta -C from aliphatic
                       20.2       2 beta -O from aliphatic
                       -2.6       1 gamma -1:C*C*C*C*C*C*1 from aliphatic
                       -7.5       general corrections
C   149.6           128.5      1-benzene
                       33.5       1 -O-C
                      -14.4       1 -O-C
                       -0.2       1 -C-C-C-C
                        2.2       general corrections
C   149.6           128.5      1-benzene
                       33.5       1 -O-C
                      -14.4       1 -O-C
                       -0.2       1 -C-C-C-C
                        2.2       general corrections
C   147.0           128.5      1-benzene
                      -14.4       1 -O-C
                       33.5       1 -O-C
                       -2.8       1 -C-C-C-C
                        2.2       general corrections
C   147.0           128.5      1-benzene
                      -14.4       1 -O-C
                       33.5       1 -O-C
                       -2.8       1 -C-C-C-C
                        2.2       general corrections
C   134.1           128.5      1-benzene
                        1.0       1 -O-C
                       -7.7       1 -O-C
                       10.9       1 -C-C-C-C
                        1.4       general corrections
C   134.1           128.5      1-benzene
                        1.0       1 -O-C
                       -7.7       1 -O-C
                       10.9       1 -C-C-C-C
                        1.4       general corrections
CH  110.4           128.5      1-benzene
                      -14.4       1 -O-C
                        1.0       1 -O-C
                       -0.2       1 -C-C-C-C
                       -4.5       general corrections
CH  110.4           128.5      1-benzene
                      -14.4       1 -O-C
                        1.0       1 -O-C
                       -0.2       1 -C-C-C-C
                       -4.5       general corrections
CH  112.2           128.5      1-benzene
                        1.0       1 -O-C
                      -14.4       1 -O-C
                       -0.2       1 -C-C-C-C
                       -2.7       general corrections
CH  112.2           128.5      1-benzene
                        1.0       1 -O-C
                      -14.4       1 -O-C
                       -0.2       1 -C-C-C-C
                       -2.7       general corrections
CH  120.3           128.5      1-benzene
                       -7.7       1 -O-C
                        1.0       1 -O-C
                       -0.2       1 -C-C-C-C
                       -1.3       general corrections
CH  120.3           128.5      1-benzene
                       -7.7       1 -O-C
                        1.0       1 -O-C
                       -0.2       1 -C-C-C-C
                       -1.3       general corrections
CH3 56.1             -2.3      aliphatic
                       49.0       1 alpha -O
                        9.3       1 beta -1:C*C*C*C*C*C*1
                        0.3       1 delta -O
                       -0.2       general corrections
CH3 56.1             -2.3      aliphatic
                       49.0       1 alpha -O
                        9.3       1 beta -1:C*C*C*C*C*C*1
                        0.3       1 delta -O
                       -0.2       general corrections
CH3 56.1             -2.3      aliphatic
                       49.0       1 alpha -O
                        9.3       1 beta -1:C*C*C*C*C*C*1
                        0.3       1 delta -O
                       -0.2       general corrections
CH3 56.1             -2.3      aliphatic
                       49.0       1 alpha -O
                        9.3       1 beta -1:C*C*C*C*C*C*1
                        0.3       1 delta -O
                       -0.2       general corrections
CH4 -2.3             -2.3      methane
                        0.0       general corrections
```

**Figure S17**. Calculated NMR spectra of model bicyclic

ChemNMR 1H Estimation

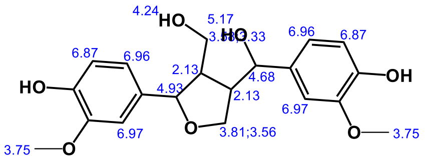


Estimation quality is indicated by color: good, medium, rough

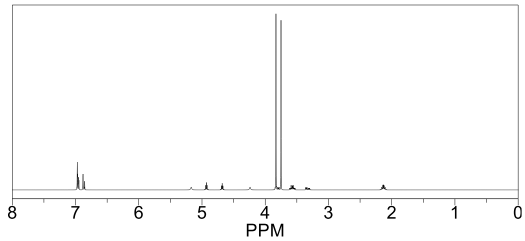


```
Protocol of the H-1 NMR Prediction (Lib=SU Solvent=DMSO 300 MHz):

Node      Shift     Base + Inc.   Comment (ppm rel. to TMS)

OH   5.17              4.20       alcohol
                          1.10       1 -C-C*R
                         -0.13       general corrections
OH   4.24              4.20       alcohol
                          0.50       1 -CC
                         -0.46       general corrections
CH   4.93              3.75       tetrahydrofuran
                          1.28       1 alpha -1:C*C*C*C*C*C*1 from methine
                         -0.10       1 beta -C from methine
CH2 3.81,3.565000         3.75       tetrahydrofuran
                         -0.06       1 beta -C from methylene
CH   2.13              1.85       tetrahydrofuran
                          0.38       1 beta -1:C*C*C*C*C*C*1 from methine
                         -0.10       1 beta -C from methine
                          0.00       1 beta -O from methine
CH   2.13              1.85       tetrahydrofuran
                         -0.10       1 beta -C from methine
                          0.38       1 beta -1:C*C*C*C*C*C*1 from methine
                          0.00       1 beta -O from methine
CH   6.97              7.26       1-benzene
                         -0.38       1 -O-C
                          0.00       1 -O-C
                          0.00       1 -C-O
                          0.09       general corrections
CH   6.97              7.26       1-benzene
                         -0.38       1 -O-C
                          0.00       1 -O-C
                          0.00       1 -C-O
                          0.09       general corrections
CH   6.87              7.26       1-benzene
                          0.00       1 -O-C
                         -0.38       1 -O-C
                          0.03       1 -C-O
                         -0.04       general corrections
CH   6.87              7.26       1-benzene
                          0.00       1 -O-C
                         -0.38       1 -O-C
                          0.03       1 -C-O
                         -0.04       general corrections
CH   6.96              7.26       1-benzene
                         -0.32       1 -O-C
                          0.00       1 -O-C
                          0.00       1 -C-O
                          0.02       general corrections
CH   6.96              7.26       1-benzene
                         -0.32       1 -O-C
                          0.00       1 -O-C
                          0.00       1 -C-O
                          0.02       general corrections
CH   4.68              1.50       methine
                          1.28       1 alpha -1:C*C*C*C*C*C*1
                          2.10       1 alpha -O
                         -0.20       2 beta -C
CH2 3.58,3.325000         1.37       methylene
                          2.20       1 alpha -O
                         -0.12       2 beta -C
CH3 3.75               0.86       methyl
                          2.87       1 alpha -O-1:C*C*C*C*C*C*1
                          0.02       general corrections
CH3 3.75               0.86       methyl
                          2.87       1 alpha -O-1:C*C*C*C*C*C*1
                          0.02       general corrections
CH3 3.83               0.86       methyl
                          2.87       1 alpha -O-1:C*C*C*C*C*C*1
                          0.10       general corrections
CH3 3.83               0.86       methyl
                          2.87       1 alpha -O-1:C*C*C*C*C*C*1
                          0.10       general corrections

1H NMR Coupling Constant Prediction

shift   atom index  coupling partner, constant and vector

5.17        21
4.24        20
4.93         7
                   11   7.0  H-C-C-H
3.69         9 diastereotopic -12.4  H-C-H
                   10   7.0  H-CH-C-H
2.13        11
                    7   7.0  H-C-C-H
                   10   7.0  H-C-C-H
                   13   7.0  H-C-CH-H
2.13        10
                    9   7.0  H-C-CH-H
                   11   7.0  H-C-C-H
                   12   7.0  H-C-C-H
6.97         5
                    1   1.5  H-C*C*C-H
6.97        15
                   19   1.5  H-C*C*C-H
6.87         2
                    1   7.5  H-C*C-H
6.87        18
                   19   7.5  H-C*C-H
6.96         1
                    2   7.5  H-C*C-H
                    5   1.5  H-C*C*C-H
6.96        19
                   18   7.5  H-C*C-H
                   15   1.5  H-C*C*C-H
4.68        12
                   10   7.0  H-C-C-H
3.45        13 diastereotopic -12.4  H-C-H
                   11   7.0  H-CH-C-H
3.75        28
3.75        29
3.83        26
3.83        27
```

ChemNMR 13C Estimation

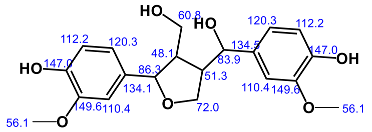


Estimation quality is indicated by color: good, medium, rough

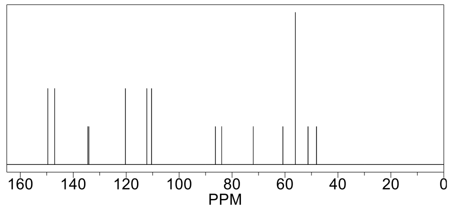


```
Protocol of the C-13 NMR Prediction: (Lib=S)

Node     Shift    Base + Inc.   Comment (ppm rel. to TMS)

CH  86.3           -8.5      tetrahydrofuran
                   24.3         1 alpha -1:C*C*C*C*C*C*1 from aliphatic
                    9.1         1 alpha -C from aliphatic
                   49.0         1 alpha -O from aliphatic
                   28.2         3 beta -C from aliphatic
                   -2.5         1 gamma -C from aliphatic
                   -6.2         1 gamma -O from aliphatic
                    0.3         1 delta -1:C*C*C*C*C*C*1 from aliphatic
                    0.6         2 delta -O from aliphatic
                   -8.0         general corrections
CH2 72.0           -8.5      tetrahydrofuran
                    9.1         1 alpha -C from aliphatic
                   49.0         1 alpha -O from aliphatic
                   28.2         3 beta -C from aliphatic
                   -5.2         2 gamma -1:C*C*C*C*C*C*1 from aliphatic
                   -2.5         1 gamma -C from aliphatic
                   -6.2         1 gamma -O from aliphatic
                    0.3         1 delta -O from aliphatic
                    7.8         general corrections
CH  48.1          -11.2      tetrahydrofuran
                   27.3         3 alpha -C from aliphatic
                    9.3         1 beta -1:C*C*C*C*C*C*1 from aliphatic
                   18.8         2 beta -C from aliphatic
                   20.2         2 beta -O from aliphatic
                   -2.6         1 gamma -1:C*C*C*C*C*C*1 from aliphatic
                   -6.2         1 gamma -O from aliphatic
                   -7.5         general corrections
CH  51.3          -11.2      tetrahydrofuran
                   27.3         3 alpha -C from aliphatic
                    9.3         1 beta -1:C*C*C*C*C*C*1 from aliphatic
                   18.8         2 beta -C from aliphatic
                   20.2         2 beta -O from aliphatic
                   -2.6         1 gamma -1:C*C*C*C*C*C*1 from aliphatic
                   -6.2         1 gamma -O from aliphatic
                   -4.3         general corrections
C   149.6         128.5      1-benzene
                   33.5         1 -O-C
                  -14.4         1 -O-C
                   -0.2         1 -C-C-C-C
                    2.2         general corrections
C   149.6         128.5      1-benzene
                   33.5         1 -O-C
                  -14.4         1 -O-C
                   -0.2         1 -C-C-C-C
                    2.2         general corrections
C   147.0         128.5      1-benzene
                  -14.4         1 -O-C
                   33.5         1 -O-C
                   -2.8         1 -C-C-C-C
                    2.2         general corrections
C   147.0         128.5      1-benzene
                  -14.4         1 -O-C
                   33.5         1 -O-C
                   -2.8         1 -C-C-C-C
                    2.2         general corrections
C   134.1         128.5      1-benzene
                    1.0         1 -O-C
                   -7.7         1 -O-C
                   10.9         1 -C-C-C-C
                    1.4         general corrections
C   134.5         128.5      1-benzene
                    1.0         1 -O-C
                   -7.7         1 -O-C
                   10.9         1 -C-C-C-C
                    1.8         general corrections
CH  110.4         128.5      1-benzene
                  -14.4         1 -O-C
                    1.0         1 -O-C
                   -0.2         1 -C-C-C-C
                   -4.5         general corrections
CH  110.4         128.5      1-benzene
                  -14.4         1 -O-C
                    1.0         1 -O-C
                   -0.2         1 -C-C-C-C
                   -4.5         general corrections
CH  112.2         128.5      1-benzene
                    1.0         1 -O-C
                  -14.4         1 -O-C
                   -0.2         1 -C-C-C-C
                   -2.7         general corrections
CH  112.2         128.5      1-benzene
                    1.0         1 -O-C
                  -14.4         1 -O-C
                   -0.2         1 -C-C-C-C
                   -2.7         general corrections
CH  120.3         128.5      1-benzene
                   -7.7         1 -O-C
                    1.0         1 -O-C
                   -0.2         1 -C-C-C-C
                   -1.3         general corrections
CH  120.3         128.5      1-benzene
                   -7.7         1 -O-C
                    1.0         1 -O-C
                   -0.2         1 -C-C-C-C
                   -1.3         general corrections
CH  83.9           -2.3      aliphatic
                   24.3         1 alpha -1:C*C*C*C*C*C*1
                    9.1         1 alpha -C
                   49.0         1 alpha -O
                   18.8         2 beta -C
                   -5.0         2 gamma -C
                   -6.2         1 gamma -O
                    0.3         1 delta -1:C*C*C*C*C*C*1
                    0.6         2 delta -O
                   -4.7         general corrections
CH2 60.8           -2.3      aliphatic
                    9.1         1 alpha -C
                   49.0         1 alpha -O
                   18.8         2 beta -C
                   -2.6         1 gamma -1:C*C*C*C*C*C*1
                   -5.0         2 gamma -C
                   -6.2         1 gamma -O
                    0.3         1 delta -1:C*C*C*C*C*C*1
                    0.3         1 delta -O
                   -0.6         general corrections
CH3 56.1           -2.3      aliphatic
                   49.0         1 alpha -O
                    9.3         1 beta -1:C*C*C*C*C*C*1
                    0.3         1 delta -O
                   -0.2         general corrections
CH3 56.1           -2.3      aliphatic
                   49.0         1 alpha -O
                    9.3         1 beta -1:C*C*C*C*C*C*1
                    0.3         1 delta -O
                   -0.2         general corrections
CH3 56.1           -2.3      aliphatic
                   49.0         1 alpha -O
                    9.3         1 beta -1:C*C*C*C*C*C*1
                    0.3         1 delta -O
                   -0.2         general corrections
CH3 56.1           -2.3      aliphatic
                   49.0         1 alpha -O
                    9.3         1 beta -1:C*C*C*C*C*C*1
                    0.3         1 delta -O
                   -0.2         general corrections
```

**Figure S18**. Calculated NMR spectra of model 17

ChemNMR $^{1}H$ Estimation

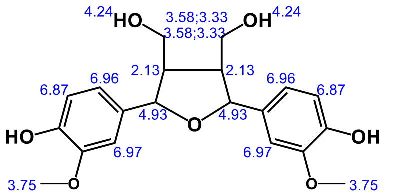


Estimation quality is indicated by color: good, medium, rough

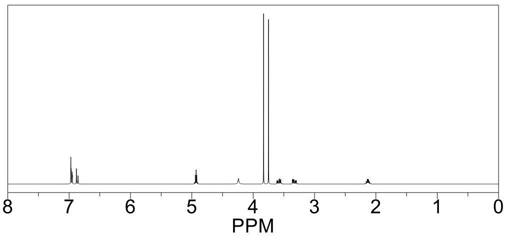


```
Protocol of the H-1 NMR Prediction (Lib=SU Solvent=DMSO 300 MHz):

Node     Shift     Base + Inc.   Comment (ppm rel. to TMS)

OH  4.24              4.20      alcohol
                        0.50       1 -CC
                       -0.46       general corrections
OH  4.24              4.20      alcohol
                        0.50       1 -CC
                       -0.46       general corrections
CH  4.93              3.75      tetrahydrofuran
                        1.28       1 alpha -1:C*C*C*C*C*C*1 from methine
                       -0.10       1 beta -C from methine
CH  4.93              3.75      tetrahydrofuran
                        1.28       1 alpha -1:C*C*C*C*C*C*1 from methine
                       -0.10       1 beta -C from methine
CH  2.13              1.85      tetrahydrofuran
                        0.38       1 beta -1:C*C*C*C*C*C*1 from methine
                       -0.10       1 beta -C from methine
                        0.00       1 beta -O from methine
CH  2.13              1.85      tetrahydrofuran
                        0.38       1 beta -1:C*C*C*C*C*C*1 from methine
                       -0.10       1 beta -C from methine
                        0.00       1 beta -O from methine
CH  6.97              7.26      1-benzene
                       -0.38       1 -O-C
                        0.00       1 -O-C
                        0.00       1 -C-O
                        0.09       general corrections
CH  6.97              7.26      1-benzene
                       -0.38       1 -O-C
                        0.00       1 -O-C
                        0.00       1 -C-O
                        0.09       general corrections
CH  6.87              7.26      1-benzene
                        0.00       1 -O-C
                       -0.38       1 -O-C
                        0.03       1 -C-O
                       -0.04       general corrections
CH  6.87              7.26      1-benzene
                        0.00       1 -O-C
                       -0.38       1 -O-C
                        0.03       1 -C-O
                       -0.04       general corrections
CH  6.96              7.26      1-benzene
                       -0.32       1 -O-C
                        0.00       1 -O-C
                        0.00       1 -C-O
                        0.02       general corrections
CH  6.96              7.26      1-benzene
                       -0.32       1 -O-C
                        0.00       1 -O-C
                        0.00       1 -C-O
                        0.02       general corrections
CH2 3.58,3.325000      1.37       methylene
                        2.20       1 alpha -O
                       -0.12       2 beta -C
CH2 3.58,3.325000      1.37       methylene
                        2.20       1 alpha -O
                       -0.12       2 beta -C
CH3 3.75              0.86      methyl
                        2.87       1 alpha -O-1:C*C*C*C*C*C*1
                        0.02       general corrections
CH3 3.75              0.86      methyl
                        2.87       1 alpha -O-1:C*C*C*C*C*C*1
                        0.02       general corrections
CH3 3.83              0.86      methyl
                        2.87       1 alpha -O-1:C*C*C*C*C*C*1
                        0.10       general corrections
CH3 3.83              0.86      methyl
                        2.87       1 alpha -O-1:C*C*C*C*C*C*1
                        0.10       general corrections

1H NMR Coupling Constant Prediction

shift   atom index  coupling partner, constant and vector

4.24        24
4.24        25
4.93         5
                    3   7.0  H-C-C-H
4.93         6
                    4   7.0  H-C-C-H
2.13         3
                    5   7.0  H-C-C-H
                    4   7.0  H-C-C-H
                    1   7.0  H-C-CH-H
2.13         4
                    6   7.0  H-C-C-H
                    3   7.0  H-C-C-H
                    2   7.0  H-C-CH-H
6.97        13
                    9   1.5  H-C*C*C-H
6.97        14
                   18   1.5  H-C*C*C-H
6.87        10
                    9   7.5  H-C*C-H
6.87        17
                   18   7.5  H-C*C-H
6.96         9
                   10   7.5  H-C*C-H
                   13   1.5  H-C*C*C-H
6.96        18
                   17   7.5  H-C*C-H
                   14   1.5  H-C*C*C-H
3.45         1 diastereotopic -12.4  H-C-H
                    3   7.0  H-CH-C-H
3.45         2 diastereotopic -12.4  H-C-H
                    4   7.0  H-CH-C-H
3.75        28
3.75        29
3.83        26
3.83        27
```

ChemNMR $^{13}C$ Estimation

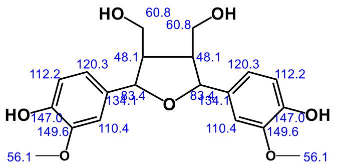


Estimation quality is indicated by color: good, medium, rough

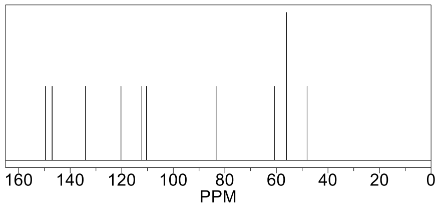


```
Protocol of the C-13 NMR Prediction: (Lib=S)

Node     Shift    Base + Inc.   Comment (ppm rel. to TMS)

CH  83.4           -8.5      tetrahydrofuran
                    24.3        1 alpha -1:C*C*C*C*C*C*1 from aliphatic
                     9.1        1 alpha -C from aliphatic
                    49.0        1 alpha -O from aliphatic
                    28.2        3 beta -C from aliphatic
                    -2.6        1 gamma -1:C*C*C*C*C*C*1 from aliphatic
                    -2.5        1 gamma -C from aliphatic
                    -6.2        1 gamma -O from aliphatic
                     0.6        2 delta -O from aliphatic
                    -8.0        general corrections
CH  83.4           -8.5      tetrahydrofuran
                    24.3        1 alpha -1:C*C*C*C*C*C*1 from aliphatic
                     9.1        1 alpha -C from aliphatic
                    49.0        1 alpha -O from aliphatic
                    28.2        3 beta -C from aliphatic
                    -2.6        1 gamma -1:C*C*C*C*C*C*1 from aliphatic
                    -2.5        1 gamma -C from aliphatic
                    -6.2        1 gamma -O from aliphatic
                     0.6        2 delta -O from aliphatic
                    -8.0        general corrections
CH  48.1          -11.2      tetrahydrofuran
                    27.3        3 alpha -C from aliphatic
                     9.3        1 beta -1:C*C*C*C*C*C*1 from aliphatic
                    18.8        2 beta -C from aliphatic
                    20.2        2 beta -O from aliphatic
                    -2.6        1 gamma -1:C*C*C*C*C*C*1 from aliphatic
                    -6.2        1 gamma -O from aliphatic
                    -7.5        general corrections
CH  48.1          -11.2      tetrahydrofuran
                    27.3        3 alpha -C from aliphatic
                     9.3        1 beta -1:C*C*C*C*C*C*1 from aliphatic
                    18.8        2 beta -C from aliphatic
                    20.2        2 beta -O from aliphatic
                    -2.6        1 gamma -1:C*C*C*C*C*C*1 from aliphatic
                    -6.2        1 gamma -O from aliphatic
                    -7.5        general corrections
C   149.6         128.5      1-benzene
                    33.5        1 -O-C
                   -14.4        1 -O-C
                    -0.2        1 -C-C-C-C
                     2.2        general corrections
C   149.6         128.5      1-benzene
                    33.5        1 -O-C
                   -14.4        1 -O-C
                    -0.2        1 -C-C-C-C
                     2.2        general corrections
C   147.0         128.5      1-benzene
                   -14.4        1 -O-C
                    33.5        1 -O-C
                    -2.8        1 -C-C-C-C
                     2.2        general corrections
C   147.0         128.5      1-benzene
                   -14.4        1 -O-C
                    33.5        1 -O-C
                    -2.8        1 -C-C-C-C
                     2.2        general corrections
C   134.1         128.5      1-benzene
                     1.0        1 -O-C
                    -7.7        1 -O-C
                    10.9        1 -C-C-C-C
                     1.4        general corrections
C   134.1         128.5      1-benzene
                     1.0        1 -O-C
                    -7.7        1 -O-C
                    10.9        1 -C-C-C-C
                     1.4        general corrections
CH  110.4         128.5      1-benzene
                   -14.4        1 -O-C
                     1.0        1 -O-C
                    -0.2        1 -C-C-C-C
                    -4.5        general corrections
CH  110.4         128.5      1-benzene
                   -14.4        1 -O-C
                     1.0        1 -O-C
                    -0.2        1 -C-C-C-C
                    -4.5        general corrections
CH  112.2         128.5      1-benzene
                     1.0        1 -O-C
                   -14.4        1 -O-C
                    -0.2        1 -C-C-C-C
                    -2.7        general corrections
CH  112.2         128.5      1-benzene
                     1.0        1 -O-C
                   -14.4        1 -O-C
                    -0.2        1 -C-C-C-C
                    -2.7        general corrections
CH  120.3         128.5      1-benzene
                    -7.7        1 -O-C
                     1.0        1 -O-C
                    -0.2        1 -C-C-C-C
                    -1.3        general corrections
CH  120.3         128.5      1-benzene
                    -7.7        1 -O-C
                     1.0        1 -O-C
                    -0.2        1 -C-C-C-C
                    -1.3        general corrections
CH2 60.8           -2.3      aliphatic
                     9.1        1 alpha -C
                    49.0        1 alpha -O
                    18.8        2 beta -C
                    -2.6        1 gamma -1:C*C*C*C*C*C*1
                    -5.0        2 gamma -C
                    -6.2        1 gamma -O
                     0.3        1 delta -1:C*C*C*C*C*C*1
                     0.3        1 delta -O
                    -0.6        general corrections
CH2 60.8           -2.3      aliphatic
                     9.1        1 alpha -C
                    49.0        1 alpha -O
                    18.8        2 beta -C
                    -2.6        1 gamma -1:C*C*C*C*C*C*1
                    -5.0        2 gamma -C
                    -6.2        1 gamma -O
                     0.3        1 delta -1:C*C*C*C*C*C*1
                     0.3        1 delta -O
                    -0.6        general corrections
CH3 56.1           -2.3      aliphatic
                    49.0        1 alpha -O
                     9.3        1 beta -1:C*C*C*C*C*C*1
                     0.3        1 delta -O
                    -0.2        general corrections
CH3 56.1           -2.3      aliphatic
                    49.0        1 alpha -O
                     9.3        1 beta -1:C*C*C*C*C*C*1
                     0.3        1 delta -O
                    -0.2        general corrections
CH3 56.1           -2.3      aliphatic
                    49.0        1 alpha -O
                     9.3        1 beta -1:C*C*C*C*C*C*1
                     0.3        1 delta -O
                    -0.2        general corrections
CH3 56.1           -2.3      aliphatic
                    49.0        1 alpha -O
                     9.3        1 beta -1:C*C*C*C*C*C*1
                     0.3        1 delta -O
                    -0.2        general corrections
```

**Figure S19**. Calculated NMR spectra of model 18

ChemNMR $^{1}H$ Estimation

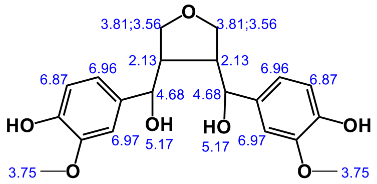


Estimation quality is indicated by color: good, medium, rough

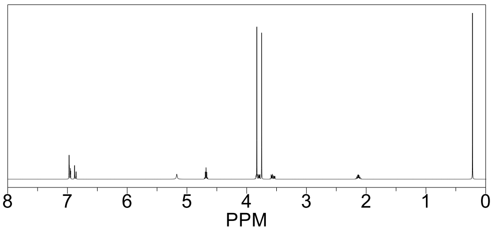


```
Protocol of the H-1 NMR Prediction (Lib=SU Solvent=DMSO 300 MHz):

Node     Shift    Base + Inc.   Comment (ppm rel. to TMS)

OH  5.17              4.20      alcohol
                         1.10      1 -C-C*R
                        -0.13      general corrections
OH  5.17              4.20      alcohol
                         1.10      1 -C-C*R
                        -0.13      general corrections
CH2 3.81,3.565000        3.75      tetrahydrofuran
                        -0.06      1 beta -C from methylene
CH2 3.81,3.565000        3.75      tetrahydrofuran
                        -0.06      1 beta -C from methylene
CH  2.13              1.85      tetrahydrofuran
                        -0.10      1 beta -C from methine
                         0.38      1 beta -1:C*C*C*C*C*C*1 from methine
                         0.00      1 beta -O from methine
CH  2.13              1.85      tetrahydrofuran
                        -0.10      1 beta -C from methine
                         0.38      1 beta -1:C*C*C*C*C*C*1 from methine
                         0.00      1 beta -O from methine
CH  6.97              7.26      1-benzene
                        -0.38      1 -O-C
                         0.00      1 -O-C
                         0.00      1 -C-O
                         0.09      general corrections
CH  6.97              7.26      1-benzene
                        -0.38      1 -O-C
                         0.00      1 -O-C
                         0.00      1 -C-O
                         0.09      general corrections
CH  6.87              7.26      1-benzene
                         0.00      1 -O-C
                        -0.38      1 -O-C
                         0.03      1 -C-O
                        -0.04      general corrections
CH  6.87              7.26      1-benzene
                         0.00      1 -O-C
                        -0.38      1 -O-C
                         0.03      1 -C-O
                        -0.04      general corrections
CH  6.96              7.26      1-benzene
                        -0.32      1 -O-C
                         0.00      1 -O-C
                         0.00      1 -C-O
                         0.02      general corrections
CH  6.96              7.26      1-benzene
                        -0.32      1 -O-C
                         0.00      1 -O-C
                         0.00      1 -C-O
                         0.02      general corrections
CH  4.68              1.50      methine
                         1.28      1 alpha -1:C*C*C*C*C*C*1
                         2.10      1 alpha -O
                        -0.20      2 beta -C
CH  4.68              1.50      methine
                         1.28      1 alpha -1:C*C*C*C*C*C*1
                         2.10      1 alpha -O
                        -0.20      2 beta -C
CH3 3.75              0.86      methyl
                         2.87      1 alpha -O-1:C*C*C*C*C*C*1
                         0.02      general corrections
CH3 3.75              0.86      methyl
                         2.87      1 alpha -O-1:C*C*C*C*C*C*1
                         0.02      general corrections
CH3 3.83              0.86      methyl
                         2.87      1 alpha -O-1:C*C*C*C*C*C*1
                         0.10      general corrections
CH3 3.83              0.86      methyl
                         2.87      1 alpha -O-1:C*C*C*C*C*C*1
                         0.10      general corrections
CH4 0.22              0.23      methane
                        -0.01      general corrections
CH4 0.22              0.23      methane
                        -0.01      general corrections

1H NMR Coupling Constant Prediction

shift   atom index  coupling partner, constant and vector

5.17       10
5.17       21
3.69        2 diastereotopic -12.4  H-C-H
                  4   7.0  H-CH-C-H
3.69        3 diastereotopic -12.4  H-C-H
                  5   7.0  H-CH-C-H
2.13        4
                  2   7.0  H-C-CH-H
                  5   7.0  H-C-C-H
                  6   7.0  H-C-C-H
2.13        5
                  3   7.0  H-C-CH-H
                  4   7.0  H-C-C-H
                  7   7.0  H-C-C-H
6.97       15
                 11   1.5  H-C*C*C-H
6.97       16
                 20   1.5  H-C*C*C-H
6.87       12
                 11   7.5  H-C*C-H
6.87       19
                 20   7.5  H-C*C-H
6.96       11
                 12   7.5  H-C*C-H
                 15   1.5  H-C*C*C-H
6.96       20
                 19   7.5  H-C*C-H
                 16   1.5  H-C*C*C-H
4.68        6
                  4   7.0  H-C-C-H
4.68        7
                  5   7.0  H-C-C-H
3.75       30
3.75       31
3.83       26
3.83       27
0.22       28
0.22       29
```

ChemNMR $^{13}C$ Estimation

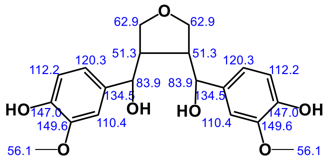


Estimation quality is indicated by color: good, medium, rough

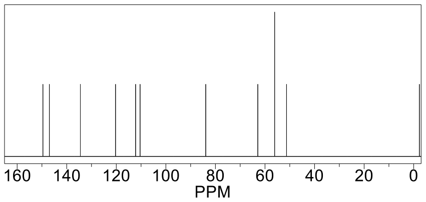


```
Protocol of the C-13 NMR Prediction: (Lib=S)

Node     Shift    Base + Inc.   Comment (ppm rel. to TMS)

CH2 62.9              -8.5      tetrahydrofuran
                        9.1       1 alpha -C from aliphatic
                       49.0       1 alpha -O from aliphatic
                       28.2       3 beta -C from aliphatic
                       -2.6       1 gamma -1:C*C*C*C*C*C*1 from aliphatic
                       -2.5       1 gamma -C from aliphatic
                       -6.2       1 gamma -O from aliphatic
                        0.3       1 delta -1:C*C*C*C*C*C*1 from aliphatic
                        0.3       1 delta -O from aliphatic
                       -4.2       general corrections
CH2 62.9              -8.5      tetrahydrofuran
                        9.1       1 alpha -C from aliphatic
                       49.0       1 alpha -O from aliphatic
                       28.2       3 beta -C from aliphatic
                       -2.6       1 gamma -1:C*C*C*C*C*C*1 from aliphatic
                       -2.5       1 gamma -C from aliphatic
                       -6.2       1 gamma -O from aliphatic
                        0.3       1 delta -1:C*C*C*C*C*C*1 from aliphatic
                        0.3       1 delta -O from aliphatic
                       -4.2       general corrections
CH  51.3             -11.2      tetrahydrofuran
                       27.3       3 alpha -C from aliphatic
                        9.3       1 beta -1:C*C*C*C*C*C*1 from aliphatic
                       18.8       2 beta -C from aliphatic
                       20.2       2 beta -O from aliphatic
                       -2.6       1 gamma -1:C*C*C*C*C*C*1 from aliphatic
                       -6.2       1 gamma -O from aliphatic
                       -4.3       general corrections
CH  51.3             -11.2      tetrahydrofuran
                       27.3       3 alpha -C from aliphatic
                        9.3       1 beta -1:C*C*C*C*C*C*1 from aliphatic
                       18.8       2 beta -C from aliphatic
                       20.2       2 beta -O from aliphatic
                       -2.6       1 gamma -1:C*C*C*C*C*C*1 from aliphatic
                       -6.2       1 gamma -O from aliphatic
                       -4.3       general corrections
C   149.6             128.5     1-benzene
                       33.5       1 -O-C
                      -14.4       1 -O-C
                       -0.2       1 -C-C-C-C
                        2.2       general corrections
C   149.6             128.5     1-benzene
                       33.5       1 -O-C
                      -14.4       1 -O-C
                       -0.2       1 -C-C-C-C
                        2.2       general corrections
C   147.0             128.5     1-benzene
                      -14.4       1 -O-C
                       33.5       1 -O-C
                       -2.8       1 -C-C-C-C
                        2.2       general corrections
C   147.0             128.5     1-benzene
                      -14.4       1 -O-C
                       33.5       1 -O-C
                       -2.8       1 -C-C-C-C
                        2.2       general corrections
C   134.5             128.5     1-benzene
                        1.0       1 -O-C
                       -7.7       1 -O-C
                       10.9       1 -C-C-C-C
                        1.8       general corrections
C   134.5             128.5     1-benzene
                        1.0       1 -O-C
                       -7.7       1 -O-C
                       10.9       1 -C-C-C-C
                        1.8       general corrections
CH  110.4             128.5     1-benzene
                      -14.4       1 -O-C
                        1.0       1 -O-C
                       -0.2       1 -C-C-C-C
                       -4.5       general corrections
CH  110.4             128.5     1-benzene
                      -14.4       1 -O-C
                        1.0       1 -O-C
                       -0.2       1 -C-C-C-C
                       -4.5       general corrections
CH  112.2             128.5     1-benzene
                        1.0       1 -O-C
                      -14.4       1 -O-C
                       -0.2       1 -C-C-C-C
                       -2.7       general corrections
CH  112.2             128.5     1-benzene
                        1.0       1 -O-C
                      -14.4       1 -O-C
                       -0.2       1 -C-C-C-C
                       -2.7       general corrections
CH  120.3             128.5     1-benzene
                       -7.7       1 -O-C
                        1.0       1 -O-C
                       -0.2       1 -C-C-C-C
                       -1.3       general corrections
CH  120.3             128.5     1-benzene
                       -7.7       1 -O-C
                        1.0       1 -O-C
                       -0.2       1 -C-C-C-C
                       -1.3       general corrections
CH  83.9               -2.3     aliphatic
                       24.3       1 alpha -1:C*C*C*C*C*C*1
                        9.1       1 alpha -C
                       49.0       1 alpha -O
                       18.8       2 beta -C
                       -5.0       2 gamma -C
                       -6.2       1 gamma -O
                        0.3       1 delta -1:C*C*C*C*C*C*1
                        0.6       2 delta -O
                       -4.7       general corrections
CH  83.9               -2.3     aliphatic
                       24.3       1 alpha -1:C*C*C*C*C*C*1
                        9.1       1 alpha -C
                       49.0       1 alpha -O
                       18.8       2 beta -C
                       -5.0       2 gamma -C
                       -6.2       1 gamma -O
                        0.3       1 delta -1:C*C*C*C*C*C*1
                        0.6       2 delta -O
                       -4.7       general corrections
CH3 56.1               -2.3     aliphatic
                       49.0       1 alpha -O
                        9.3       1 beta -1:C*C*C*C*C*C*1
                        0.3       1 delta -O
                       -0.2       general corrections
CH3 56.1               -2.3     aliphatic
                       49.0       1 alpha -O
                        9.3       1 beta -1:C*C*C*C*C*C*1
                        0.3       1 delta -O
                       -0.2       general corrections
CH3 56.1               -2.3     aliphatic
                       49.0       1 alpha -O
                        9.3       1 beta -1:C*C*C*C*C*C*1
                        0.3       1 delta -O
                       -0.2       general corrections
CH3 56.1               -2.3     aliphatic
                       49.0       1 alpha -O
                        9.3       1 beta -1:C*C*C*C*C*C*1
                        0.3       1 delta -O
                       -0.2       general corrections
CH4 -2.3               -2.3     methane
                        0.0       general corrections
CH4 -2.3               -2.3     methane
                        0.0       general corrections
```

**Figure S20**. Calculated NMR spectra of model 19

## References


1. Ralph, S.A., Ralph, J. & Landucci, L.L. (2009) NMR database of lignin and cell wall model compounds, https://www.glbrc.org/databases_and_software/nmrdatabase/.
2. Zhu, X., Akiyama, T., Yokoyama, T., & Matsumoto, Y. (2019). Lignin-biosynthetic study: reactivity of quinone methides in the diastereopreferential formation of *p*-hydroxyphenyl-and guaiacyl-type β-O-4 structures. Journal of Agricultural and Food Chemistry, 67(8), 2139-2147.
3. Toikka, M., & Brunow, G. (1999) Lignin-carbohydrate model compounds. Reactivity of methyl 3-O-(α-L-arabinofuranosyl)-β-D-xylopyranoside and methyl β-D-xylopyranoside towards a β-O-4-quinone methide. Journal of the Chemical Society, Perkin Transactions 1, 1877-1883.
4. Zeng, X., Akiyama, T., Yokoyama, T., Matsumoto, Y. (2020) Contribution of the γ-hydroxy group to the β-O-4 bond cleavage of lignin model compounds in a basic system using tert-butoxide, Journal of Wood Chemistry and Technology, 40(5), 348-360.
5. Brunow, G., Sipilä, J., & Mäkelä, T. (1989). On the Mechanism of Formation of Non-cyclic Benzyl Ethers During Lignin Biosynthesis. Part 1. The Reactivity of β-O-4 Quinone Methides with Phenols and Alcohols. Holzforschung, 43(1), 55-59.
6. Sipilä, J., & Brunow, G. (1991). The synthesis and NMR-spectroscopical characterization of tetrameric lignin model compound with non-cyclic benzyl aryl and aryl ether bonds. Proceedings of the 6th International Symposium on Wood, Fiber and Pulping Chemistry, Helbouvne, Australia, vol. 2, pp. 1-3.
7. Adler, E. (1952). The beta-guaiacyl ether of alpha-veratrylglycerol as a lignin model. Svensk Pepperstidn, 55, 245-254.
8. Zhu, X. Model Study on the Water Addition Reaction to Quinone Methide Intermediates during Lignin Biosynthesis. Doctoral dissertation, The University of Tokyo, 2019. Available online: https://repository.dl.itc.u-tokyo.ac.jp/records/2004230#.Yunb8HZByUk.
9. Adler, E., Brunow, G., & Lundquist, K. (1987). Investigation of the acid-catalysed alkylation of lignins using NMR spectroscopic methods. Holzforschung, 41(4), 199-207.
10. Zhu, X., Akiyama, T., Yokoyama, T., & Matsumoto, Y. (2019). Lignin-biosynthetic study: reactivity of quinone methides in the diastereopreferential formation of p-hydroxyphenyl-and guaiacyl-type β-O-4 structures. Journal of agricultural and food chemistry, 67(8), 2139-2147.
